# Integration of Epigenetic Mechanisms into Non-Genotoxic Carcinogenicity Hazard Assessment: Focus on DNA Methylation and Histone Modifications

**DOI:** 10.3390/ijms222010969

**Published:** 2021-10-11

**Authors:** Daniel Desaulniers, Paule Vasseur, Abigail Jacobs, M. Cecilia Aguila, Norman Ertych, Miriam N. Jacobs

**Affiliations:** 1 Environmental Health Sciences and Research Bureau, Hazard Identification Division, Health Canada, AL:2203B, Ottawa, ON K1A 0K9, Canada; 2 CNRS, LIEC, Université de Lorraine, 57070 Metz, France; paule.vasseur@univ-lorraine.fr; 3 Independent at the Time of Publication, Previously US Food and Drug Administration, Rockville, MD 20852, USA; Abbyj96210@gmail.com; 4 Toxicology Team, Division of Human Food Safety, Center for Veterinary Medicine, US Food and Drug Administration, Department of Health and Human Services, Rockville, MD 20852, USA; Cecilia.Aguila@fda.hhs.gov; 5 German Centre for the Protection of Laboratory Animals (Bf3R), German Federal Institute for Risk Assessment, Diedersdorfer Weg 1, 12277 Berlin, Germany; Norman.Ertych@bfr.bund.de; 6 Centre for Radiation, Chemical and Environmental Hazards, Public Health England, Chilton OX11 0RQ, UK; miriam.jacobs@phe.gov.uk

**Keywords:** epigenetics, DNA methylation, histone modifications, carcinogens, non-genotoxic, risk assessment, mode of action, IATA, AOP

## Abstract

Epigenetics involves a series of mechanisms that entail histone and DNA covalent modifications and non-coding RNAs, and that collectively contribute to programing cell functions and differentiation. Epigenetic anomalies and DNA mutations are co-drivers of cellular dysfunctions, including carcinogenesis. Alterations of the epigenetic system occur in cancers whether the initial carcinogenic events are from genotoxic (GTxC) or non-genotoxic (NGTxC) carcinogens. NGTxC are not inherently DNA reactive, they do not have a unifying mode of action and as yet there are no regulatory test guidelines addressing mechanisms of NGTxC. To fil this gap, the Test Guideline Programme of the Organisation for Economic Cooperation and Development is developing a framework for an integrated approach for the testing and assessment (IATA) of NGTxC and is considering assays that address key events of cancer hallmarks. Here, with the intent of better understanding the applicability of epigenetic assays in chemical carcinogenicity assessment, we focus on DNA methylation and histone modifications and review: (1) epigenetic mechanisms contributing to carcinogenesis, (2) epigenetic mechanisms altered following exposure to arsenic, nickel, or phenobarbital in order to identify common carcinogen-specific mechanisms, (3) characteristics of a series of epigenetic assay types, and (4) epigenetic assay validation needs in the context of chemical hazard assessment. As a key component of numerous NGTxC mechanisms of action, epigenetic assays included in IATA assay combinations can contribute to improved chemical carcinogen identification for the better protection of public health.

## 1. Introduction

Epigenetics encompasses mechanisms that regulate DNA functions (e.g., gene transcription, DNA replication, repair, and stability). Epigenetic programing is fundamental for normal mammalian development and provides a mechanism by which the environment can rapidly alter gene expression within single or multiple generations. The working definition of epigenetics utilised for the purpose of this work is “the study of molecular processes that influence the flow of information between a constant DNA sequence and variable gene expression patterns. This includes investigation of nuclear organization, DNA methylation, histone modification and RNA transcription. Epigenetic processes can result in intergenerational (heritable) effects as well as clonal propagation of cell identity without any mutational change in DNA sequence” (Nature, 2021: https://www.nature.com/subjects/epigenetics) (accessed on 19 June 2021). The complete epigenetic status of a cell at any given time is termed the epigenome. Even if all cells carry the same DNA, during development, the epigenetic system establishes tissue-specific patterns of DNA methylation [1], of histone post-translational modifications (HPTM) [2] and of chromatin-interacting non-coding RNAs. The epigenome permits the expression (or inhibition) of gene profiles necessary to accomplish the specialised functions of each tissue in an individual. The establishment of the epigenetic marks is a gradual process initiated during embryonic development and cellular differentiation, continuing through tissue development [3,4], and postnatally [5,6,7].

The induction of adverse DNA mutations by environmental factors that lead to disease, particularly cancer, is well established and recognized by regulatory bodies worldwide [8]. As such, testing procedures and safety guidelines are in place to protect human health against the adverse effects of chemically induced genetic mutations. In the regulatory sphere, whilst some sectors such as the agrochemical sector, under regulatory jurisdiction requirements, can conduct rodent cancer bioassays that can detect non-genotoxic carcinogens (NGTxC), other sectors have regulatory restrictions to do so [9]. Often, once mutagenicity and genotoxicity testing have been conducted, no further testing for the detection of NGTxC is requested or performed. To date, there is a lack of sufficient regulatory test methods to assess non-genotoxicity modes of action. This is recognised internationally as a critical gap toward the optimal protection of public health [9,10].

The importance of epigenetics in cancer biology is firmly established; “Cancer genetics and epigenetics are inextricably linked in generating the malignant phenotype; epigenetic changes can cause mutations in genes, and, conversely, mutations are frequently observed in genes that modify the epigenome” [11]. Alterations of the epigenetic system are common in cancers [12] whether the initial carcinogenic events are derived from GTx or NGTx mechanisms. Therefore, the induction of epigenetic anomalies represents a mode of action to be considered in chemical hazard assessment for all carcinogens and offers possibilities for the development of assays to improve the detection of NGTxC. As such, epigenetic assays are being considered for addition to an integrated approach to the testing and assessment (IATA) of NGTxC, which is under development as a project of the Organisation for Economic Cooperation and Development (OECD) Test Guideline Programme [10]. A typical IATA is defined as “a structured approach used for hazard identification (potential), hazard characterization (potency) and/or safety assessment (potential/potency and exposure) of a chemical or group of chemicals, which strategically integrates and weighs all relevant data to inform regulatory decision regarding potential hazard and/or risk and/or the need for further targeted testing and therefore optimizing and potentially reducing the number of tests that need to be conducted” [13,14]. The IATA for NGTxC intends to integrate and weigh all available NGTxC data for hazard assessment purposes, with the following key events considered to be more pivotal for protection at earlier stages of carcinogenic development: inflammation, immune dysfunction, mitogenic signalling, sustained cell proliferation, cell injury, and cell transformation leading to a change in morphology [10]. Epigenetic mechanisms underpin all these cancer hallmarks and characteristics [15].

DNA methylation (DNAm) involves the addition of a methyl group, primarily to the carbon-5 of cytosine within cytosine–phosphate–guanine dinucleotides (CpGs) to produce 5-methylcytosine (5mC). DNA hypermethylation of promoters can silence tumor suppressor genes, whereas DNA hypomethylation can activate expression of oncogenes, retrotransposons, and non-coding RNAs, and can also destabilize structural satellite DNA repeats [16]. These anomalies can originate from site-specific changes in epigenetic enzyme abundance and activities. It has been suggested that the transfer of epigenetic methylating or hypomethylating enzyme complexes across normal genomic boundaries (limited by CCCTC-binding factor (CTCF) [17], or by differential methylation vs. hydroxymethylation abundance [18]) could favour spreading of DNA hypomethylation (in oncogene promoters) or hypermethylation (in enhancers or promoters of tumor suppressor genes) in adjacent genomic areas [19,20,21,22,23]. Such phenomena are also demonstrated with inserted reporter constructs [24]. A recent review summarized how epigenetic DNA modifications (5mC, 5-hydroxymethylcytosine (5hmC)) influence mutagenesis [25].

The nucleosome is the structural unit of chromatin (protein–DNA complex), composed of histone octamers (two copies each of H2A, H2B, H3, and H4) which are small basic positively charged proteins. The negatively charged DNA winds around nucleosomes, and the “linker” histone H1 locates on the nucleosome, binds to the DNA and keeps it in place around the nucleosome. DNA condensation and its packaging into the nucleus are further enabled by looping formed by DNA-interacting condensins and cohesins. The presence of nucleosomes inhibits DNA accessibility that can be regained by nucleosome displacement or eviction by the SWI/SNF families of ATP-dependent chromatin-remodeling complexes, which are necessary for DNA-related functions (transcription, replication, and repair). Mutations in the SWI/SNF family components are found in more than 20% of all cancers [26,27,28]. Histone tail post-translational modifications (HPTM), such as acetylation, methylation, or ubiquitination of lysine (K), methylation of arginine (R), and phosphorylation of serine (S), are additional epigenetic marks altering the affinities of interacting proteins to modulate chromatin structure and functions [29]. While acetylation reduces the positive charge of histones and is limited to acetylation or deacetylation, histone methylation is a more complex system with combination of mono (me), di (me2), or trimethylated (me3) sites that are not affecting the positive charge of the lysine residue. Generally, actively transcribed regions (euchromatin) are associated with methylated H3K4, H3K36, or H3K79, which can be located beside silenced bivalent regions characterized by the presence of H3K27me3 and H3K9me2 [30,31]. Constitutive heterochromatin is associated with H3K9me3 [32] that attracts heterochromatin protein-1 (HP1) and DNA methyltransferases to collectively repress gene expression [33]. A list of 67 H3-PTM enzymes (families of HMT, KDM, HAC, and HDAC) were reported to be significantly increased or decreased in expression, in 16 types of cancers relative to normal tissues [34].

There are numerous reviews describing the effects of exposure to chemical substances on the epigenetic system using laboratory models [7,35,36,37,38,39,40,41,42], and epidemiological investigations [43,44,45,46,47]. The application of epigenetic investigations to chemical hazard assessment has not been progressing as quickly as the field of clinical epigenetics. The clinical benefits of epigenetics are numerous, providing information toward the diagnostic of disease subtypes, toward innovative epigenetic drugs and oncology treatments, and toward prognostic predictions [48,49,50,51]. While clinical epigenetics aims to understand and cure diseases, it shares a common goal with chemical hazard assessment; that of identifying epigenetic markers predictive of carcinogenicity to ensure that new chemical substances or new drugs do not have the undesirable side effect of deregulating the epigenetic system toward oncogenicity. In this respect, a number of short-term epigenetic enzymology assays have been developed (examples in Section 8).

**Figure 1:**
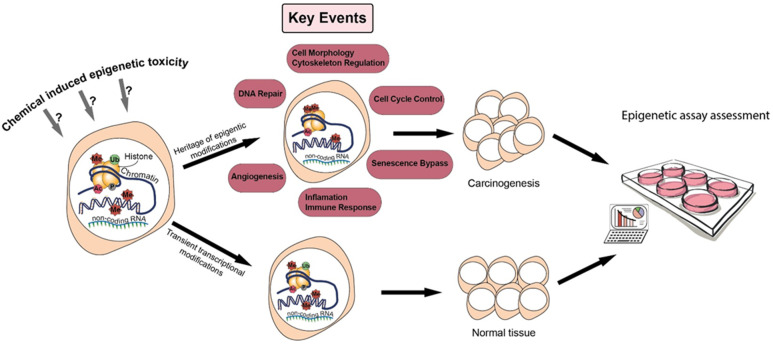
This literature review aims at identifying relevant epigenetic mechanisms and assays with high potential for optimization and validation in the context of chemical hazard assessment, and for contributing to the design of an IATA for NGTxC.

In the present work, our goal (Figure 1) is to review the literature to assess the relevant epigenetic mechanisms and assays, with the intent of selecting those that have a high potential for optimization and validation in the context of chemical hazard assessment, and for contributing to the design of a successful OECD IATA for NGTxC [10].

The current work includes Section 1, Section 2, Section 3, Section 4, Section 5 and Section 6 and an overall conclusion (Section 7) as the main document that positions cancer epigenetics within the framework of the IATA for NGTxC and of chemical hazard assessment. Section 8, Section 9, Section 10 and Section 11 provide additional in depth information. Section 2 provides an overview of cancer epigenetics focusing on DNAm with interplay with histone modifications and ncRNAs and suggests how both NGTxC and GTxC substances can directly and indirectly affect the epigenetic system and contribute to carcinogenesis. Section 3 positions epigenetic endpoints across the key events of the NGTxC IATA and highlights technical considerations for improving the value of epigenetic data for easier inclusion into chemical hazard assessments. Section 4 examines three principal case study chemical examples, arsenic, nickel and phenobarbital (with some additional chemical examples), for which there is sufficient evidence of epigenetic effects for the identification of both common and distinct epigenetic alterations, thereby uncovering key roles of epigenetic anomalies in carcinogenesis. Section 5 provides a list of epigenetic endpoints known to be affected during carcinogenesis, together with a small subset of epigenetic assays, which could be considered, with further optimization and validation, for the NGTxC IATA integration. A selection of relevant epigenetic assays, in combination with other non-epigenetic assays, will thereby contribute to the detection of NGTxC. Finally, Section 6 proposes a strategy that involves key questions that may help evaluators in assessing the importance of cancer epigenetic data in chemical hazard assessments.

The present work does not include a critical review of non-coding RNAs as epigenetic components contributing to the carcinogenicity of NGTxC. Long non-coding RNAs and microRNAs (miRNAs) have multiple targets involved in carcinogenesis. DNAm and histone modifications can regulate miRNA expression, and reciprocally numerous miRNAs regulate expression of DNMTs, histone-modifying enzymes, and methyl-binding proteins [52,53].

## 2. Epigenetics and Carcinogenesis

### 2.1. Metabolism Pathways as Epigenetic Regulators

**Figure 2:**
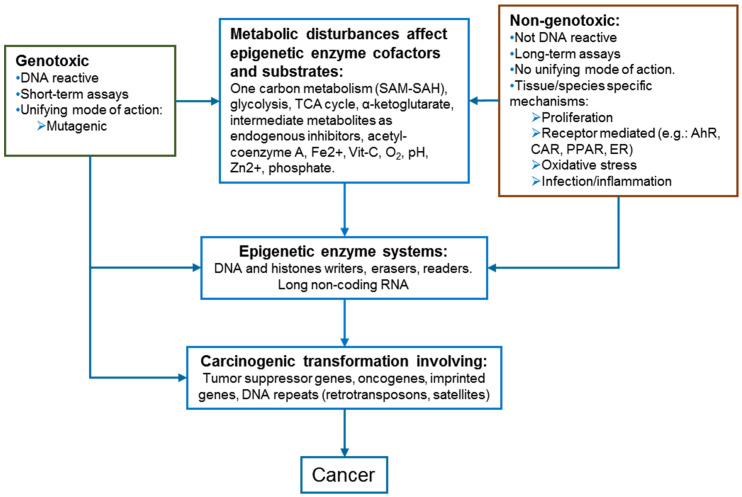
Schematic relationship between epigenetics, non-genotoxic and genotoxic mechanisms.

In addition to mutations in epigenetic driver genes, metabolic deficiencies can induce epigenetic disturbances and reprograming that may lead to carcinogenic events. Metabolic changes can reduce the availability of substrates and co-factors necessary to maintain optimal epigenetic enzyme activities. Enzyme kinetic analyses have demonstrated that histone acetyltransferases (HATs), histone methyltransferases (HMTs), and DNA methyltransferases (DNMTs) can be affected by metabolic deficiencies, whereas ubiquitin ligases and kinases are less likely to be affected [54]. Figure 2 shows a schematic relationship between epigenetics, NGTxC and GTxC mechanisms. While the early carcinogenic events for GTxC involve interaction with DNA (with or without metabolic transformation) as a unifying mechanism of action that can affect various oncogenes, NGTxC do not follow a unified mechanism of action [55]. NGTxC, as well as GTxC, can induce various metabolic disturbances (energy, lipid, and one carbon metabolism, co-factors, iron and zinc availability, oxidative stress, endogenous enzyme inhibitors) that can compromise epigenetic enzyme activities, and are likely be conducive to deregulated expression of carcinogenic genes and DNA repeats.

Carcinogenesis involves abnormal activation of the glycolytic pathway under aerobic conditions instead of oxidative phosphorylation (Warburg effect) and diverts glycolytic intermediates into biosynthetic pathways (supplying nucleotides, proteins, lipids) that permit rapid cell division in cancers [56,57]. Deregulation of energy metabolism is an early step in carcinogenesis [57] and is one of the hallmarks of cancer [56]. Anomalies in the energy metabolism pathways can change the abundance of intermediate metabolites that are co-factors and others that are endogenous inhibitors of epigenetic enzymes [58]. However, the earliest stage of carcinogenesis at which energy metabolism is affected and whether these events can be precipitated by exposure to NGTxC remain ill defined.

Alterations in metabolic pathways, induction of detoxification reactions and oxidative stress are all early events following exposure to substances that contribute to epigenetic disturbances and are extensively reviewed [58,59,60,61,62,63,64]. In the following section, the potential for oxidative stress to act as an epigenetic regulator is initially discussed. Next, six selected family groups of epigenetic enzymes regulating the chromatin structure [31,65], that are proposed to be potentially affected by altered metabolism [54], and that can lead to global or site-specific changes in DNAm, to histone modifications, and ultimately to carcinogenic events, are discussed.

#### 2.1.1. Oxidative Stress as an Epigenetic Regulator

Both energy metabolism (such as one-carbon metabolism, glycolysis, and the Krebs cycle) and oxidative stress (through induction of glutathione synthesis by the trans-sulfuration and one-carbon metabolism pathways) are known regulators of the epigenetic system. The induction of oxidative stress is a frequent outcome following exposure to a wide range of substances [66,67]. Detoxification reactions from Phase-I enzymes (e.g., oxidation by cytochrome p450 enzyme families) can contribute to oxidative stress when abundance of endogenous antioxidant, or Phase-II (e.g., glutathione conjugation) enzymes are deficient [68]. 8-oxo-7,8-dihydroguanosine (8-oxo-dG) is an indicator of DNA exposure to reactive oxygen species. The presence of 8-oxo-dG during replication leads to GC to TA transversion in the replicated strand. The 8-oxo-dG can be repaired by the DNA 8-oxoguanine glycosylase (OGG1), which is reported to form a complex with TET1 to also demethylate 5mC [69]. Interestingly, the activity of other demethylases, KDM (LSD1 and LSD2), and nuclear oxidases release H_2_O_2_ and can promote the formation of 8-oxo-dG [64]. It is noted that: (1) 8-oxo-dG interferes with DNMTs and with methyl-CpG-binding proteins, (2) there is a negative correlation between 8-oxo-dG and 5mC, and (3) the expression of some genes is regulated by the presence of 8-oxo-dG. Collectively, these observations suggest that 8-oxo-dG is not only a by-product of oxidative stress but is also an epigenetic mark that can control gene expression [64]. Oxidative stress also influences histone methylation and acetylation [70,71,72] (discussed in Section 2.1.1, Section 2.2, Section 2.4, Section 4.1.1 and Section 4.2.3). The causal relationship between oxidative stress and morphological transformation and its inhibition by vitamin E has been demonstrated in Syrian hamster embryo (SHE) cell culture [73,74]. (historically referred to “embryo” but in fact the SHE cells are derived from fetuses at 13–14 days of gestation; a more than 50-year-old legacy of literature in chemical carcinogenicity assays [75,76]) (See Section 4.2.3 for the effects of nickel on hypoxia and oxidative stress.)

### 2.2. Methyltransferases

Methyltransferases, including DNMTs and HMTs, rely on the one-carbon metabolism pathway for the supply of the universal methyl donor S-adenosylmethionine (SAM) as the substrate for the methylation reaction [77]. DNA methyltransferases (DNMT1, 3a, 3b) add a methyl group to cytosines mostly in CpG dyads to generate 5-methyl 2′-deoxy-cytidine (5mC). Methylation occurs in other dyads but at a much lower frequency. *S*-adenosylhomocysteine (SAH) is the methylation reaction by-product that can inhibit methyltransferases, but a positive SAM/SAH ratio is conducive to methylation [78,79]. There are examples of external factors modulating methyltransferase activities. Diet (e.g., intake of folate, choline, or betaine), detoxification, and oxidative stress, affect the *trans*-sulfuration reaction producing cysteine and glutathione, and can affect the availability of SAM [80]. SHE cell assay data further support links between one-carbon metabolism and cell transformation. SAH hydrolase is a tumor suppressor enzyme [81] reported to downregulate in di(2-ethylhexyl)phthalate (DEHP)-treated SHE cells [82]. Choline is known to be an important cellular metabolite and methyl donor in the one-carbon metabolism pathway, and diethanolamine reduces the uptake of choline to induce SHE cell morphological transformation [83]. Developmental exposure to environmental contaminants such as a mixture of polychlorinated biphenyls (PCBs) reduced the hepatic abundance of SAM and DNA methyltransferases in the rat [84]. 2,3,7,8-tetrachlorodibenzo-*p*-dioxin (TCDD) also alters the hepatic one-carbon metabolism pathway and abundance of SAM in mice [85].

It should be noted that some epigenetic enzymes also target non-nuclear proteins, for example the histone methyltransferase SETD2 (Su(var)3-9, Enhancer of Zeste, Trithorax-domain-containing 2; alias KMT3A) that is responsible for H3 lysine 36 trimethylation (H3K36me3), and methylation of the cytoskeleton alpha-tubulin at lysine 40, the same lysine that may be marked by acetylation [86,87]. The latter reinforces the occurrence of mutually exclusive acetylation and methylation. Therefore, deleterious effects of exogenous chemicals on the activity of this enzyme can alter histone and cytoskeleton functions and cause mitotic spindle and cytokinesis defects, micronuclei, and polyploidy [86,87].

Collectively, these observations support understanding of how chemical exposure can induce oxidative stress, alteration of the one-carbon metabolism pathway, and of methyltransferase activities that can lead to cellular transformation.

### 2.3. Demethylases

Another set of epigenetic enzyme families that can be metabolically affected includes demethylases that are dioxygenases which actively remove methyl groups from DNA [Ten Eleven Translocation enzyme family (TET1-3)], or from histones lysine (K) or arginine (R) residues [KDM2 (FBXL), KDM3 (JMJD1), KDM4 (JMJD2/JHDM3), KDM5 (JARID1), KDM6 (UTX/JMJD6)]. Note that the lysine-specific histone demethylase 1A (LSD1(KDM1A)) targeting H3K4me2/3 is a flavin-dependent monoamine oxidase [88], that induces demethylation via a different mechanism than the dioxygenases [89]. Methylation marks can be actively removed or passively removed through failure of deposition through cell replication. The TET enzymes oxidize 5-methylcytosine (5mC) to 5-hydroxymethylcytosine (5hmC), and the latter can be further oxidized by the TET enzymes to 5-formylcytosine (5fC) and 5-carboxylcytosine (5caC), which are then actively removed by thymine-DNA glycosylase (TDG) and the base excision repair mechanism. Induction of the stress-response protein GADD45 (growth arrest and DNA damage-inducible 45 beta and alpha) alters DNA demethylation by interacting with TET1 [90], thymine DNA glycosylase [91], and DNA repair complex [92,93] (see Section 4.3 on phenobarbital).

5hmC is not only an oxidised derivative for demethylation of 5mC, as they both have different functional roles [94]; 5mC and 5hmC are considered to be the fifth and sixth base of DNA [95]. The various methods that use the sodium bisulphite reaction to identify sites with 5mC cannot distinguish 5mC from 5hmC, which can confound data interpretation. Methods to measure 5mC and 5hmC separately, as well as their functional roles in part mediated through specific binding proteins, are discussed in Section 11.2.

Dioxygenases acting on histones or on DNAm are dependent on mitochondrial production of α-ketoglutarate (also identified as 2-oxoglutarate), Fe^2+^ (ferrous ion), vitamin C (Vit-C) [96,97], and oxygen availability [98], and therefore are sensitive to environmental changes under in vivo stress and in vitro conditions [99,100]. Vit-C acts as an electron donor to recycle and reduce Fe^3+^ (ferric ion) into biologically active Fe^2+^. The importance of Vit-C is such that a deficit can to a large extent recapitulate TET1 mutation on epigenetic reprogramming and mammalian reproduction [101]. The availability of α-ketoglutarate is dependent on the tricarboxylic acid cycle (TCA/Krebs cycle), during which the conversion of citrate to α-ketoglutarate is catalyzed by the enzyme isocitrate dehydrogenase (IDH). Mutation in this enzyme is found in some cancers and leads to the production of 2-hydroxyglutarate (a competitive inhibitor of α-ketoglutarate), resulting in the arrest of cellular differentiation [102,103]. Succinate and fumarate are other TCA cycle intermediate metabolites that are endogenous competitive antagonists of α-ketoglutarate [63]. Mitochondrial metabolism can regulate cell function and gene expression pathways by changing abundance of α-ketoglutarate, thereby affecting dioxygenases involved in the hypoxia response and DNA/histone modifications. Bailey et al. [98] showed that increased α-ketoglutarate/succinate ratio promotes embryonic stem cell pluripotency, and antagonises the growth of solid organ tumours through increased 5hmC and histone demethylation. Moreover, they identified ABHD11 (αβ-hydrolase domain containing 11) as a mitochondrial enzyme required to maintain TCA cycle integrity, and that can potentially alter cell fate decisions [98].

Optimal activities of dioxygenases (involved in regulation of hypoxia, demethylation of DNA, and of histones) are dependent on the maintenance of oxygen availability, oxidative stress, or acidity (that also affects histone deacetylases) created by the glycolytic production of lactic acid [104]. TET1 enzyme activity can respond to hypoxia. Its activity is differentially regulated within the embryogenesis physiologically relevant range of oxygen (TET1 activity is affected at ≤5% O_2_ and inhibited at 1% O_2_) [61]. Aerobic conditions are regulated by the transcription factor hypoxia-inducible factor-1 (HIF-1α), which forms a heterodimer with the constitutively expressed HIF-1β (also known as the aryl-hydrocarbon receptor nuclear translocator (ARNT)), to induce target gene expression in response to hypoxia. In the presence of oxygen, the dioxygenase prolyl hydroxylase (PHD) hydroxylates HIF-1α, a modification that directs HIF-1α to the ubiquitin pathway for degradation. However, under low-oxygen conditions, the efficiency of PHD decreases and HIF1-α remains stable, dimerizes with HIF-1β, and this transcription factor complex can bind the hypoxia response elements to induce relevant gene expression to regain normoxia. PHD is another Fe^2+^-dependent dioxygenase reliant on oxygen and α-ketoglutarate, which is in turn inhibited by succinate and fumarate [105]. ARNT (HIF-1β) is also required in detoxification reactions; upon activation of the aryl-hydrocarbon receptor (AhR) by ligands such as B[a]P, PCB, polychlorinated-dibenzodioxins (PCDD), or -dibenzofurans (PCDF), ARNT (HIF-1β) is required for the translocation of the receptor complex AhR/ARNT (HIF-1β) to the nucleus for the induction of detoxification genes. Consequently, hypoxia regulation and the detoxification systems are induced by the heterodimers HIF-1α/HIF-1β (ARNT) and the AhR/ARNT (HIF-1β), respectively, and both systems compete for the availability of ARNT (HIF-1β) [106,107]. Thus, exposure to NGTxC that bind AhR and sequester ARNT (HIF-1β) reduces the efficiency of the HIF-1 system, with consequent lower oxygen availability and reduced activities of other dioxygenases involved in epigenetic demethylation.

Collectively, these mechanisms can modify the abundance of 5mC and 5hmC across the genome and deregulate gene expression pathways.

### 2.4. Histone Acetylation

Histone acetyltransferases (HAT, e.g., CBP, p300) using acetyl-coenzyme A (ac-CoA) as an acetyl donor, and histone deacetylases using Zn^2+^ (HDAC family I, II, and IV) or NAD+ (Sirtuins HDAC family III) as a co-factor, are additional sets of metabolically responsive epigenetic enzymes. Conditions (e.g., oxidative stress, starvation) leading to fluctuations of ac-CoA or NAD+ alter their activities [70,108]. Differences in the global amount of histone acetylation are reported between normal and cancer cells, lower amounts are associated with the more aggressive phenotypes [109]. Histone acetylation is an important modification of the chromatin regulating DNA transcription, replication and repair. Acetylation neutralizes the positive charges of histones and weakens interactions with the negatively charged DNA, which consequently reduces chromatin compaction and favours DNA accessibility. In euchromatin, the processes of acetylation and deacetylation can be rapid. For example, the half-life of histone acetylation marks is within a few minutes but those of methylation marks vary from less than a day to more than four days for H4K20me3 [110,111]. Therefore, given these differences in half-lives, the measurement of chemical effects on global genome changes in acetylation reflects induction of rapid disequilibrium in enzymatic reactions (necessary for the rapidly changing euchromatin), and as such, longer experiments may be required to investigate impacts on methylation marks and in slowly changing heterochromatin [112].

Histone deacetylation contributes to tumour suppressor gene silencing. Deacetylation reactions are responsive to oxidative stress, to metabolic changes and to altered pH [58]. HDACs with conserved cysteine residues reduce their activities upon oxidative stress due to the formation of a disulfide bond between cysteine thiol groups [108]. Changes in intracellular pH, as a result of metabolic disturbances or acidic microenvironment, are buffered by various systems including histone deacetylation. By monitoring the abundance of acetylated histones (H4ac, H4K12ac, H4K16ac, and H3K18ac) in human cancer cell lines and embryonic stem cells, McBrian et al. [113] demonstrated that as the internal pH decreases, histones are globally deacetylated by HDAC and the released acetate anions are co-exported with protons outside the cells preventing further reduction in intracellular pH, but with consequences in the expression of numerous genes. Interestingly, they observed contrasting results with cytoskeleton α-tubulin acetylation that was minimally affected by pH, but highly acetylated in nutrient deprived media, while histones were deacetylated; this suggests that remaining pools of acetyl-coA have a selective allocation under starvation conditions. The epigenetic response to acidic environment (pH 6.5, 24 h) include DNA hypomethylation, reduced HDAC1 expression, and enhanced H3-Ac in normal primary cells, but without effect in an osteosarcoma cell line [104]. However, an acidic environment (pH 6.2) promotes a more aggressive phenotype in cells derived from a low metastatic variant of Lewis lung carcinoma [114] and morphological transformation of SHE cells (pH 6.7 [115,116]).

HDAC can be inhibited by four types of chemicals (hydroxamic acid-based chemicals, benzamide derivatives, short-chain fatty acid-based chemicals, and cyclic peptides) with known mechanisms of action [117]. With the exception of Class III HDACs (the Sirtuins family), HDACs are Zn-dependent enzymes, and therefore, HDAC inhibitors include a Zn-binding domain [118]. Such characteristics can be instructive in predicting other potentially disruptive substances on the basis of structure–activity relationships. In summary, histone (and cytoskeleton) acetylation can rapidly respond to metabolic disturbances, and the sensitivity to pH can differ between cell types.

### 2.5. Mechanisms of DNA Hyper or Hypomethylation

5mC is an important heritable epigenetic mark present in variable abundance across genomic regions and impacting on the regulation of their DNA functions (heterochromatin, promoters, enhancers, intron–exon boundaries, and intragenic sites). DNAm is accomplished by complexes of proteins that vary in composition depending on where they are located (replication fork, or other genomic locations) and on the phases of the cell cycle. DNMT can be found in association with polycomb proteins, transcription factors, heterochromatin readers, and replication associated proteins, and such complexes target and modulate DNMT activities to specific DNA regions [119]. Non-coding RNAs (ncRNAs) can recruit DNMT complexes to alter DNAm and histones to regulate gene transcription [120,121,122]. The presence of histone modifications also modulates DNAm. There is a strong positive association between H3K9 methylation and DNAm in differentiated cells, whereas H3K4 methylation inhibits DNMT activities [123,124]. Weinberg et al. [125] demonstrated that DNAm of promoters, gene bodies, or of intergenic regions was influenced by the relative abundance of H3K36me2 (methylated by NSD1/2), H3K36me3 (methylated by SETD1/2), and H3K4me3 (methylated by SETD1A/1B and MLL methyltransferase family). They summarise that in euchromatin, the presence of H3K4me3 in promoters prevents interaction with the de novo methylators DNMT3A or 3B and the lack of DNAm favours the recruitment of the TET enzyme family (DNA demethylases) and H3K36 demethylase KDM2B. KDM2B demethylates H3K36me2 and H3K4me3 [126]. In contrast, in intergenic regions, DNMT3A interacts with H3K36me2, and in gene bodies DNMT3B is guided by H3K36me3 to methylate CpG sites [125]. There are clearly complex counterbalancing interactions among the chromatin components involved in modulating the activity of maintenance DNA methyltransferase (DNMT1), cooperation between DNMT1 and de novo methyltransferases DNMT3A and 3B [119,127], with specificity of genomic regions. Importantly, such interactions are deregulated during carcinogenesis, leading to hypo or hypermethylation.

#### 2.5.1. DNA Hypermethylation

Mechanisms of DNA hypermethylation (methylation in excess of the basal control level) are associated with an increased abundance of DNMT, slippage of DNMT complexes across epigenetic boundaries, and reduced TET enzyme activities. The activated carcinogenic KRAS pathway in the mouse fibroblast cell line NIH3T3 can lead to DNA hypermethylation, TSG silencing, cell transformation including anchorage-independent growth and tumorigenicity [128,129]. It was initially suggested that abnormal RAS pathway signaling increases expression of DNMT1 (but not DNMT3A or 3B) contributing to TSG silencing by promoter hypermethylation (e.g., *Fas, Sfrp1, Par4, Plagl1, H2-K1* and *Lox*) [129]. However, a more recent study indicates that DNMT1 expression is not increased by KRAS activation; rather, KRAS activation induces the expression of miRNA (miR)-29b, which represses expression of the demethylase TET1, with a consequent increase DNAm and silencing of TSG promoters, as in the case of the pro-apoptotic *Fas* gene [128]. They also showed reversible effects, such that inhibition of miR-29b restored activity of TSGs and specifically expression of TET1, TET3 and DNMT3B by 5-fold, 2-fold and 4-fold, respectively.

Abnormal epigenetic functioning can induce DNA hypermethylation that silences TSG promoters and/or enhancers, but other regions known as “canyons” (regions normally having the lowest amount of DNAm) can also become hypermethylated. Hypermethylation of the canyon associated with homeobox oncogenes (*Dlx1*, *Pou3f3*) increases their expression instead of the usual decrease in expression associated with promoter hypermethylation [130]. In mESC, the presence of TET1 in canyons and in proximal promoters establishes the boundaries preventing DNAm by DNMT3A1 [131].

In the context of a chemical testing strategy, DNA hypermethylation can lead to both decreases and increases in gene expression. In addition, the reversible effects of activated KRAS pathway mediated by miR-29b on DNAm and TSG silencing suggest that DNAm changes in promoters can be transient. Therefore, abnormal signaling should be maintained over time to induce such adverse outcomes.

#### 2.5.2. DNA Hypomethylation

Global genome DNA hypomethylation (GGDHo) occurs in many cancers [132,133], except perhaps in acute lymphocytic leukemia [134]. Given the normal distribution of DNAm across the genome, GGDHo is mostly attributed to loss of methylation occurring in late-replicating heterochromatic domains and in intragenic regions [133,134]. The abundance of DNAm varies across the normal genome. Only 4% of CpG sites are methylated in “canyons” targeted by transcription factors (TF); in contrast, high levels of methylation are observed in heterochromatic DNA repeated sequences such as retrotransposons (*Line* and *Sine* with 89% and 90% methylation, respectively) and in intragenic regions (83%) [135]. Consequently, these are the regions that largely contribute to GGDHo.

Loss of DNAm during malignant transformation is suggested to involve passive demethylation due to the accelerated state of cell division and a delay in maintenance of methylation [132]. Note that spontaneous deamination of 5mC leading to C to T transition mutation also contributes to passive demethylation [136]. However, proliferation rates cannot explain GGDHo measured in histologically normal cells surrounding tumours [137,138]. GGDHo can be an early carcinogenic event, that can be measured in epithelial metaplasia/dysplasia [139], and in tumour-associated fibroblasts [140]. Such events are also referred to as “effects in bystander cells”, “field effects”, or “field defects” [137,138]. The Supplementary Table S1 lists examples of investigations reporting progressive changes in DNAm associated with cancers and field effects. Mechanisms leading to GGDHo may include both active processes mediated by TET enzyme activities, and passive mechanisms involving decreases in DNMT activities during DNA replication. There are also several emerging mechanistic explanations for GGDHo. As a first example, in human breast cancer MCF7 cells and mouse embryonic stem cells (mESC), the over-expression of the protein arginine (R) methyltransferase-6 (PRMT6) that deposits the histone marks H3R2me2 and that impairs UHRF1 recruitment on the replicating chromatin, prevents the formation and activity of the DNMT1/UHRF1 complexes for maintenance methylation [141]. In a second example, the DNMT1/UHRF1 complex formation can also be inhibited by a pluripotency marker DPPA3, that binds and displaces UHRF1 from the chromatin [142]. In a third example, Hervouet et al. [119] summarised that decreases in DNMT1 expression can be induced by miR-21, -148a, and 29b, but a decrease in expression of DNMT1 is rarely observed in solid tumours. They suggest that reduction in activity is mostly attributable to post-translational modification of DNMT1, including phosphorylation, methylation, or sumoylation [119]. With a fourth example, where KRAS activation induces site-specific hypermethylation (Section 2.5.1), HRAS is also noted to contribute to GGDHo by reducing the expression of DNMT3B [143]; DNMT3B not only performs de novo methylation but also cooperates with DNMT1 in the methylation of highly methylated genomic areas [119,144]. A fifth example for the reduction in DNA methylation is the impairment of the UHRF1 ubiquitylation activities. UHRF1 ubiquitylation of PCNA-associated factor 15 and of H3 is necessary to recruit DNMT1 to replicating domain [145] and to the maintenance of DNAm in H3K9me3 labelled chromatin [123]. Finally, CpG damage induced by oxidative stress or inflammatory reactions can reduce affinity of methyl binding protein and DNMT activities [146,147] or can attract TET enzymes [69]. The 8-oxo-dG can be repaired by the DNA 8-oxoguanine glycosylase (OGG1), which forms a complex with TET1 to also demethylate 5mC [69]. Collectively, these observations indicate how global genome DNAm in mammalian cells can be reduced through many alternative mechanisms, but the direct link between H3K9me3 and DNMT1-mediated maintenance DNAm that influences genome stability [123] is clearly of particular utility for chemical hazard assessment.

##### Consequences of Global Genome DNA Hypomethylation

Whilst the DNAm of CpG islands is mostly known for tumour suppressor gene silencing, GGDHo can be conducive to altered gene expression, oncogene and retrotransposon activation [148]; functional deficiencies in structural DNA repeats [149]; genetic and chromosomal instability; and ultimately to carcinogenesis. These observations are based on substantial experimental evidence. GGDHo has been shown to promote mutations [150], and to be associated with activation of epigenetically silenced oncogene (e.g., *Met* oncogene [151]). In mice, experimental induction of DNA hypomethylation by the transfection of defective DNMT1 induces tissue-specific effects including (1) the development of aggressive T cell lymphomas with chromosomal instability (trisomy of chromosome 15) [152,153], (2) activation of rodent-specific transposon (leading to the generation of an oncogenic form of NOTCH1 by the transposition of the intracisternal A particle (IAP) into the *Notch1* locus) [154], (3) loss of heterozygosity (LOH) at the *Apc* gene and elevated incidence of intestinal microadenomas and growth reduction in macroscopic intestinal tumors [155], and finally, (4) development of multifocal liver tumours accompanied by *Apc* LOH [155].

Indices of GGDHo can be derived by the measurement of methylation in DNA repeated sequences. Examples of DNA repeated sequences include the mutagenic retrotransposons long and short interspersed nuclear element (*Lines* and *Sines*, e.g., *Line-1* or *L1* and the *Sine AluYb8*), and structural centromeric (e.g., *satellite-alpha*, *Sat-α*) and juxtacentromeric DNA repeated sequences (*Satellite-2*, *Sat-2*) [156,157]. Methylation measurements of these DNA sequences are often reported as indices of GGD methylation due to their distribution across the genome. However, methylation abundance in these sequences does not always correlate either amongst each other, or with GGDHo.

DNAm silences retrotransposons, and GGDHo can be interpreted as a predisposing condition to retrotransposition events. *L1* remnants represent 17% of the human genome [158], and only a few *L1* sequences are complete with an intact promoter and functional elements enabling retrotransposition (the “hot-*L1*” [158]). Despite the limited numbers of functional L1, L1-retrotransposition represents an important contribution to the mutation spectrum of cancers occurring in 53% of cancer patients—most frequently observed in colon (93%) and lung cancers (75%) [158]. GGDHo occurs with aging associated with *L1* activation that leads to genomic instability. *L1* cDNA cytoplasmic accumulation triggering the cGAS DNA sensing pathway and a type I interferon response (increased abundance of Interferon α and β) creates ageing pathological inflammation [159,160].

Intragenic DNAm is abundant in actively transcribed genes, where it stimulates gene expression and has regulatory functions [161]. Housekeeping genes form the exception [162]. Therefore, in addition to GGDHo activating mutagenic retrotransposons, intragenic hypomethylation can also reduce the expression of more favourable and protective genes [163,164]. It can deregulate alternative splicing and promote transcription initiation at alternative promoters and at cryptic transcription start sites, leading to the expression of abnormal transcripts [165]. Intragenic DNAm mechanisms involve the RNA polymerase II, which recruits the methyltransferase SETD2/KMT3A, creating the histone marks H3K36me3 that attract DNMT3B for intragenic methylation of actively transcribed genes [164]. In introns, however, intragenic epigenetic repression involves the CCCTC-binding factor (CTCF) that contributes to the recruitment of the histone demethylase KDM4A, which reduces the abundance of H3K36me2/3 and thus transcription [163]. Similarly, CTCF are associated with KDM5B to demethylate H3K4me3 (a marker of active genes) and pause RNA polymerase II [166]. It can be assumed that the repressive activity of intronic complexes is reduced in exons by DNAm given that DNAm prevents the binding of CTCF to approximately 40% of the CTCF binding sites [17,167]. Using mESC, Neri et al. [164] demonstrated that DNMT3B methylation within gene body prevents aberrant transcription initiation. They also re-emphasise that fact that abnormal transcripts in cancer cells can originate from intragenic hypomethylation, defective functions of DNMT3B, SETD2/KMT3A, and H3K36 methylation. Finally, hydroxymethyl cytosine (5hmC), a stable oxidised form of 5mC [168], is also more abundant within gene bodies of normal cells than in cancer cells [169], and its intragenic roles deserve further investigation. Collectively, this information suggests that GGDHo can adversely affect the expression of intragenic DNAm-dependent genes, and activation of abnormal/cryptic transcription start sites (TSS), CTCF and KDM occupancies.

As discussed above, GGDHo can have adverse consequences in normal cells, but it can also have anti-tumorigenic effects by inducing re-expression of silenced TSG [16] and reducing expression of oncogenes that are dependent on intragene DNAm. The treatment of acute myeloid leukemia (AML) illustrates a benefit derived from induced hypomethylation of intragene DNAm. Treatment of AML cells with the DNMT inhibitor 5-aza-2′-deoxycytidine (5aCdR) and HDAC inhibitors (panobinostat or valproic acid) in vitro synergistically downregulated the oncogenes MYC associated with gene body DNA demethylation, overexpressed epigenetic modifiers (e.g., KDM2B and SUV39H1), and changes in acetylated H3K9/K27 [170]. Another example is that for colorectal cancer cells that develop an immunogenic response derived from DNA hypomethylation. Here, 5aCdR-induced hypomethylation activates cryptic non-annotated TSS, which leads to the expression of: (1) endogenous retroviral element double-stranded RNA (dsRNA) activating the MDA5/MVS/IRF7 immune pathway, (2) non-coding RNAs, and (3) RNAs coding to functional proteins and immunogenic non-functional proteins [171,172]. These events represent unsuspected immunogenic mechanisms that contribute to the elimination of colorectal cancer cells.

In summary, while drug-induced GGDHo and histone modifications have therapeutic benefits in the treatment of some cancers [173], the induction of DNA hypomethylation in normal cells can promote carcinogenesis by activating proto-oncogenes, retrotransposons, cryptic TSS, and the promotion of chromosome instability.

##### Global Genome DNA Hypomethylation and Chromosome Instability

GGDHo can destabilize structural DNA repeats in centromeric and pericentromeric areas, leading to overexpression of satellite repeats, defective centromere and kinetochore functions, chromosome instability, and aneuploidy [174]. The centromeres and pericentromeres include simple DNA repeats (satellite-alpha (*Sat-α*), *beta-, gamma-*, *-I*, *-II*, *-III)*, transposons, long terminal repeats (*Ltr*), and non-*Ltr* retrotransposons (*Line*, *Sine*). Pericentromeric regions are composed of heterochromatin with their abundant silencing marks (H3K9me2/3, HP1, H3K27me2/3, H4K20me2/3, and cytosine and adenine methylation) but these regions can be transcribed. These silencing epigenetic marks exert a precise transcription control of repeated sequence non-coding RNAs that are required for the stabilization of the centromere to prevent its recombination, and for recruiting cohesin rings for sister chromatid cohesion [174]. SAT-α non-coding RNAs are essential for the insertion of centromeric variant histone-3 protein (centromeric protein CENP-A, -B, and -C) into the DNA, and for the recruitment of protein complexes for kinetochore assembly. The latter orients the segregation of replicated chromosomes during mitosis and meiosis. CENP-C recruits DNMT3A/3B to promote methylation and reduce local transcription. DNMT3B methylates intragenic regions of active genes, subtelomeric, centromeric, and pericentromeric repeats [144]. Overexpression of SAT-α RNAs leads to eviction of histone variant proteins (e.g., CENP-A), to DNA hypomethylation, to destabilization of the centromeric/pericentromeric areas and of the kinetochore, and also to chromosome instability and aneuploidy [149,174]. Therefore, epigenetic anomalies inducing either over- or under-expression of repeated sequences have adverse effects.

Overexpression of satellite DNA has been reported in numerous conditions; it can be chemically induced and may therefore be a useful marker. In human cells, satellite DNA overexpression is observed during stress, senescence, aging, and in various cancers associated with deregulation of epigenetic enzyme activities (e.g., DNMT3B, KDM2A, JMJD2B, SIRT6, GCN5, BRCA1, PRC1, and PRC2) [174]. SAT-II RNA expression is noted to increase in the serum of patients with precancerous pancreatic lesions, and even more so in patients with pancreatic ductal adenocarcinomas [175]. Satellite RNA expression can be detected in 3D pancreatic cancer cell cultures (BxPC3), but not in 2D cultures, and while SAT-α RNAs are the most abundant, SAT-II RNAs have provided the most accurate data [175]. SAT-III expression from pericentromeres is weakly induced by etoposide (a DNA damaging topoisomerase inhibitor), methyl methanesulfonate (an alkylating agent), and the DNA polymerase inhibitor aphidicolin. It is moderately affected by UV radiation or hyperosmosis, but strongly increased by heat shock and exposure to the heavy metal cadmium [174]. In murine cells, 5aCdR (DNMT inhibitor) or staurosporine (apoptosis inducer) induces the expression of murine-specific satellite repeats and mitotic defects [174]. DNAm of *Sat-α* measured by pyrosequencing [156,176] and *Sat-II* by MethyLight [149,157] are hypomethylated in cell lines relative to normal cells. Therefore, incorporation of measured DNAm and RNA changes from satellite DNA deserves consideration within the NGTxC IATA.

## 3. Positioning Epigenetics in Chemical Hazard Assessment

### 3.1. Epigenetics in Adverse Outcome Pathways to Overcome the Multiplicity of NGTxC Modes of Action

For chemical hazard assessment purposes, the adverse outcome pathway (AOP) concept is being increasingly used to organize toxicological information into mechanistic/mode of action, sequence of events starting from the molecular initiating event (MIE), and leading to a successive series of key events (KE) that are required to generate the adverse outcome at the end of the sequence [13]. Epigenetics is a broad field with many potential endpoints, and the selected terminology to describe the epigenetic events (MIE, KE, associated event (AE), and mode of action (MOA)) is dependent on the context, the evidence, and the available understanding of both a contributory and causal relationship of the epigenetic changes to a specific event or to an apical/adverse outcome.

Using epigenetic knowledge within the AOP construct suggests that an epigenetic event can be a MIE, a KE, or an AE [177]. There has been abundant recent literature demonstrating that disruption of epigenetic modifiers (these are “writers”, “erasers”, or “editors”) are by themselves drivers of carcinogenesis [65,178]. Deregulated expression of any of 25 epigenetic toxicity pathway components (SET1, MLL1, KDM5, G9A, SUV39H1, SETDB1, EZH2, JMJD3, CBX7, CBX8, BMI, SUZ12, HP1, MPP8, DNMT1, DNMT3A, DNMT3B, TET1, MeCP2, SETDB2, BAZ2A, UHRF1, CTCF, HOTAIR and ANRIL) can induce human cellular transformation, suggesting that individually these epigenetic components and non-coding RNAs can be drivers of carcinogenesis [179].

**Figure 3:**
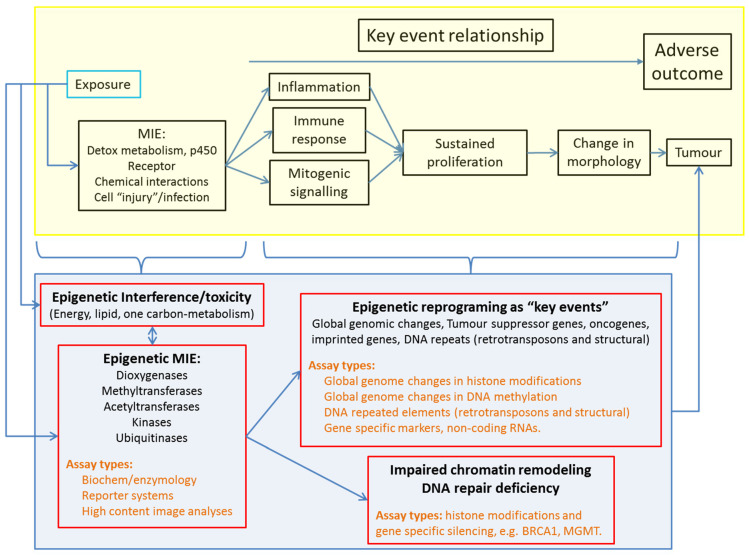
Positioning of epigenetic assay types relative to the AOP flow in order to monitor their contribution to initiating events and key events during carcinogenesis. The AOP flow used to develop the NGTxC IATA [10] is shown modified in the upper yellow rectangle. Carcinogens induce an MIE through direct or indirect interactions that subsequently create a series of key events that will lead to an adverse outcome; in this case, a tumour. The lower blue rectangle indicates examples of epigenetic assay types that can be used to monitor the key events, starting with metabolic alterations and interference with epigenetic enzyme expression and activities as potential MIE, and then indicators of heritable epigenetic reprogramming of key events that may have long-term carcinogenic impact. Epigenetic alterations can be induced by both genotoxic and non-genotoxic carcinogens. The integration of epigenetic assays together with other data types constitutes the IATA. (Acronyms: p450, cytochrome p450; BRCA1, breast cancer type 1 susceptibility protein; MGMT, O6-methylguanine-DNA methyltransferase).

NGTxC have several mechanisms of action that raise the challenge of developing a generic testing strategy that can identify early epigenetic anomalies as a common event induced by various NGTxC. To overcome this challenge, epigenetic assays, in combination with other assay types in the context of the IATA, could be designed to target KE common to numerous cancers and their progression (Figure 3). This would permit the detection of NGTxC even if their actions were variable and tissue specific.

Figure 3 positions epigenetic events relative to the recently proposed key events of an IATA for NGTxC [10]. Following chemical exposure, the cancer IATA in the upper yellow box proposes MIE such as p450 enzyme induction leading to oxidative stress or chemical biotransformation, or receptor interactions, then these various MIE can lead to KE relationships between cancer hallmarks/characteristics that are often held in common across cancer types. Figure 3 shows how impaired chromatin remodeling and deficient DNA repair can be consequences of altered activities of epigenetic enzymes, also be early events across cancer types. DNA damage normally arises via DNA replication and transcription, replication fork collapse, or from reactions of DNA with endogenous by-products such as reactive oxygen species. DNA repair must occur at all times and chromatin plasticity regulated by the epigenetic system has been firmly linked to efficient DNA repair [180]. For example, repair of double-strand breaks conducted by the 53BP1 error-prone nonhomologous DNA end-joining pathway and the BRCA1 error-free homologous recombination pathway is dependent upon the coordination of epigenetic systems involving: (1) methylation of H4K20 mediated by lysine methyltransferases (KMT5A/B/C, MMSET), and (2) demethylases, acetylase, ubiquitinase and epigenetic reader proteins (i.e., JMJD2A (alias KDM4A), L3MBTL1, MBTD1, and TIP60) [180]. Chemically induced epigenetic disturbances can therefore contribute to or be at the origin of mutagenic events. Disturbance in methylation processes and/or chromatin rearrangement affecting the epigenetic system can contribute to DNA repair deficiencies in any cell types. These deficiencies can also be induced by any NGTxC inducing sufficient “cellular stress” (defined by occurrence of reactive oxygen species, oncogene activation, telomere erosion, stalled replication forks [181], and by oxidative, proteotoxic, endoplasmic reticulum, genotoxic, hypoxic, and metabolic stress [182]).

### 3.2. Adverse Epigenetic Effects vs. Normal Epigenetic Regulation and Disturbances

It is challenging to develop a testing strategy that can distinguish between normal epigenetic regulation following chemical exposure, as opposed to adverse epigenetic reprograming causally linked to carcinogenesis. For example, enrichment of H3K27ac in the promoter region of induced genes is a normal process [183], and not indicative of adversity even when detected following chemical exposure. In contrast, epigenetic changes associated with a carcinogenic key event can provide mechanistic understanding of adversity. Numerous investigations have demonstrated that epigenetic disruptions can be at the origin of mutations and genomic instability leading to tumour development [65,184,185,186]. In addition to the spontaneous deamination of 5mC leading to C to T transition mutation [136], other epigenetic changes can trigger mechanisms (e.g., silencing genes for p16^INK4a^, RB, BRCA1, MLH1, and MGMT; activation of retrotransposons and DNA repeat instability) that can eventually lead to genetic mutations, deletions, insertions, translocations, and chromosomal aneuploidy, all of which are indicators of genome instability conducive to carcinogenesis [187]. Therefore, results from relevant epigenetic assays may trigger the need for complementary assays in the NGTxC IATA.

Long(er)-term, or follow-up of, experiments may be required to better understand if an epigenetic change represents an adverse epigenetic reprograming linked to carcinogenesis. For example, nickel induced not only persistent changes in gene expression, but also newly emerging gene expression changes due to epigenetic deregulation, months after termination of exposure [188,189]. TET enzyme activities were both reduced and increased by short- and long-term exposures to arsenic, respectively [190,191]. Consequently, it is recommended that demonstrating epigenetic reprograming requires a combination of short- and longer-term assays; short-term assays may highlight direct interactions with epigenetic proteins/enzymes, whereas longer-term assays may identify those genes that have been epigenetically reprogramed with activity modified to a persistent or heritable altered state.

Epigenetic assays may assist in revealing inadequate interpretation of transcriptomic analyses, and as such are complementary to each other. While the expression of TSG or oncogenes may appear normal, it could in fact be deregulated by adverse epigenetic events. Low expression of cell cycle regulators, such as p16^INK4a^, can be observed in both normal and cancerous cells, but in the latter cell type the expression can be suppressed by epigenetic silencing of the *INK4b*/*ARF*/*INK4a* locus. The complexity of this locus and its role in regulating the expression of other cell cycle regulators are addressed in Section 9.2.

An epigenetic difference in one gene, even if linked to altered gene expression, has limited weight for consideration as an AE. There are numerous genes involved in each carcinogenic KE (Figure 3) and the function of a single gene can frequently be compensated for via molecular pathway/network crosstalk and compensatory talk. In addition, a single gene has limited predictive value as compared to a signature gene set given that various cancers demonstrate different epigenetic anomalies. The concept of “methylator phenotypes” (detailed in Section 5.3 and Section 10.2), which represents selected gene sets affected by abnormal methylation and of clinical diagnostic and prognostic value [192,193] can be a more useful strategy. This approach can be developed to target KE of carcinogenesis, such as metabolic reprograming [57], evasion of growth suppression [194], senescence bypass [195] (Vaccari et al. in preparation), or immune evasion [196] (Corsini et al. in preparation).

### 3.3. Dose/Concentration–Response, Response Threshold and Magnitude, and Assay Duration

Table 1 summarises the technical characteristics needed for suitable epigenetic assays for chemical hazard assessment purposes. There should be sufficient understanding of the epigenetic assay to capture the significance of the chemically induced epigenetic change (orchestrated by the 426–450 epigenetic regulators [197,198]) to carcinogenesis. Such understanding should permit the consideration of the epigenetic change either as MIE, KE, AE [177], or adaptive event [199,200], within the context of the cancer KER (Figure 3), such that its contribution may be evaluated as part of a weight of evidence approach within a toxicity/carcinogenicity assessment.

Data utility is greater where epigenetic endpoints can be compared to apical endpoints [201]. Epigenetic assays with a large dynamic range (difference between the positive and negative controls [202]) would be useful to facilitate: (1) comparison to apical endpoint; (2) identification of PoD (e.g., BMD, NOAEL, and LOAEL) to establish the margin of exposure relative to estimates of human exposure [203]; (3) detection of effects even if there could be variability across laboratories or experiments; and (4) the identification of a threshold response above baseline to enable classification of a chemical. Whilst the field of epigenetic toxicity is maturing, ensuring that the relevant assays provide reproducible data is essential.

The nature of epigenetic modifications poses challenges in generating concentration- or dose–response assessment. The dynamic range of the abundance of epigenetic marks is limited to the number of genomic loci and to the number of cells affected in a sample (this contrasts with mRNA or protein expression that are the outcomes of intrinsic cellular amplification systems from a gene). The propagation of epigenetic changes may occur throughout adapting cells within a tissue or culture, and the heritable marks may spread across the cell populations following delays sufficient to allow for multiple cellular divisions. Even the measurements of DNAm abundance in DNA repeated sequences that are distributed across the genomes in high copy numbers are generally insufficient to generate concentration–response patterns with a large dynamic range. Moreover, such assessments can be biased by mixed cell populations in a sample homogenate due to responsive and resistant cells, or due to the invasion of immune cells carrying different epigenetic profiles that can confound the target cells’ epigenome [204]. Finally, epigenetic responses may occur in a non-monotonic pattern, for example DNMT mRNA abundance following organochlorine exposure in rats [205], or postnatal exposure to BPA that has been found to decrease promoter methylation of some genes, but which occurs at low but not high doses [206].

### 3.4. Assay Robustness and Reproducibility

There are numerous methods available for the measurement of DNAm [207,208,209], but the disappointing rate of using DNAm measurement as a reliable biomarker has been attributed to a lack of validation, unclear clinical value [210], inaccuracies of older technologies (e.g., clonal bisulphite sequencing), methodological caveats, and differences among techniques [48,211]. Section 11.1 summarises a series of validation exercises that were performed to validate DNAm measurements [48,211,212]. Overall, relatively recent pyrosequencing assays for site-specific changes and next-generation sequencing (NGS)-based techniques for genome-wide studies were found to offer versatile and reliable approaches for investigating DNAm in various types of samples. Similar validation experiments are still needed for other epigenetic endpoints.

Relative to numbers of differentially expressed genes in various studies, only a limited number of genes demonstrate a typical relationship between promoter DNAm and gene expression (hypermethylation with decreased expression, or hypomethylation with increased expression) [213,214,215,216]. These associations may be obscured by biological factors, or by inappropriate NGS bioinformatics criteria for a correct interpretation of the data within and across studies. These may be due to several factors. First, DNAm is only one amongst the diverse epigenetic modulators that may play a key role [217]. Measuring methylation variability among individuals within groups instead of average values was found to be a better marker of changes in gene expression [218]. Second, the change in DNAm will not have an impact if the appropriate transcription factors to induce the corresponding gene expression are absent, and this can depend upon the stage of cellular transformation reached (fully developed diseases show more methylation-expression correlations). Third, the changes in DNAm may not target the relevant DNA sequence in the promoter or enhancer regulating gene expression. Fourth, the bioinformatics criteria used to call a methylation level can be restrictive and insensitive (e.g., number of CpGs considered to calculate an average methylation level in a differentially methylated region, size of window, criteria to define a negative background). Fifth, the use of the sodium bisulphite reaction to identify 5mC, but that cannot distinguish 5mC from 5hmC (Section 11.2 discusses this aspect further). Some of these limitations are being mitigated with new laboratory techniques [219] and new computational epigenomic tools [220]. With the advance of technology and knowledge, the robustness and reproducibility of assays can be improved.

### 3.5. Extrapolation from In Vitro Culture Systems

With the increasing understanding of cell culture conditions that may affect the epigenetic system and where biases may occur in the interpretation of effects induced by chemicals [100], there is a need for consistent and transparent reporting formats [221], as being developed for omics reporting at the OECD. Mass spectrometry analyses demonstrate that HPTM alterations occur with the transitions from the original tissues to primary cell cultures, and then to (immortalised, transformed and transfected) cell lines, with losses of acetylation marks being the earliest changes. Indeed, more differences occurring between tissues and cell lines have been noted than differences between cancer types [222]. Consideration with respect to cell culture variables in the generation of epigenetic data are further elucidated in Section 11.3 and Section 11.4.

Effects of NGTxC as well as epigenetic mechanisms are often tissue and cell specific; consequently, the relevance of a cell culture model can depend on knowledge of tissue-targets, on the apical endpoint requiring investigation, and on the anticipated exposure route (oral, inhalation, dermal). In the absence of toxicokinetic information, the liver is the organ with the most exposure after oral ingestion and is often selected as a sentinel model tissue, as it is the target organ for biotransformation. While the liver is the target tissue for the majority of carcinogens [223], many non-genotoxic substances or their metabolites are carcinogenic but not to the liver (see “IARC-List of classifications by cancer site”, https://monographs.iarc.fr/agents-classified-by-the-iarc/) (accessed on 5 October 2021). Consequently, the cell culture model should be carefully selected to improve the weight of evidence in the hazard assessment process.

### 3.6. Extrapolation of Experimental Model to Human

In the absence of clear human data, experimental animal and cell culture models are essential in predicting toxicity to humans in the context of an IATA [9,10]. If animal experiments are required, a list of OECD Test Guidelines in which epigenetic assays can be integrated has been established [7,224]. It must be noted that interspecies epigenetic differences, including the distribution of epigenetic marks and repeated sequences that exert regulatory roles, differ across genomes of human, non-human primates [225,226], and other laboratory models [227], and therefore this impedes direct extrapolation of findings to humans [3]. Beyond epigenetics, differences in carcinogenesis between animal models and human were raised many years ago [228,229]. Nevertheless, at the current time, animal models are still fundamentally essential in research and regulatory chemical hazard assessment (see Section 11.3), though a very strong move toward novel approach methodologies (NAMs) is being effected. Extrapolating epigenetic data from experimental models to humans requires careful mechanistic considerations.

## 4. Epigenetic Disturbances Induced by Genotoxic and Non-Genotoxic Carcinogens

Causal relationships between the disturbance of epigenetic pathway components and carcinogenic human cell transformation are compelling [179]. This section provides examples of a third layer to this understanding, suggesting a causal relationship between chemical exposure, induction of epigenetic disturbances, and carcinogenesis.

Even if chemical carcinogens (GTx or NGTx) induce cancers as a unifying outcome, the induced epigenetic changes may differ depending on the chemical and on the carcinogenic pathways that are activated. Indeed, 7 NGTxC with different modes of action induced different DNAm profiles 10 to 15 days after transformation of the v-Ha-RAS-transfected BALB/c 3T3 Bhas42 cell line [230,231]. Similarly, Fonti et al. [232] investigated the chronology of genomic changes after immortalizing primary human mammary epithelial cells (HMEC) with a shRNA inactivating TP53, and adding a second oncogenic vector to activate different oncogenic pathways, either CCNE1 (coding for CYCLIN E1), WNT1, or HRAS^v12^. They observed changes in miRNA and mRNA expression, followed by DNAm changes, and later by gene copy number alterations (CNA). The HMEC transformed by HRAS had clearly distinct patterns of genetic and epigenetic modifications compared to CCNE1 or WNT1 transformed cell lines, whereas the latter two induced similar CNA and DNAm patterns [232]. These findings indicate that oncogenic pathways and epigenetic changes can change depending upon the early activation of pathways in different transformed cell lines, and this complicates the identification of a “standardised” carcinogenic epigenetic signature for NGTxC using different models and with different modes of action.

The scope of the following section is limited to the review of examples of carcinogenic substances (elemental arsenic, arsenic salts, and arsenic compounds, elemental nickel, nickel salts, and nickel compounds, phenobarbital) and of irradiation, for which their epigenetic effects were sufficiently investigated to identify mechanisms that can offset chromatin functions and be informative in the perspective of developing epigenetic testing strategies. This section also reveals that chemicals can induce epigenetic alterations and cancer predisposition through mechanisms that are chemical specific, in combination with mechanisms that are common across chemicals. For example, the induction of oxidative stress is a common mechanism induced by exposure to heavy metals [189], or to many other chemicals and to ionizing radiation. However, the magnitude of induced oxidative stress differs across treatments, e.g., cobalt induces approximately 3-fold more oxidative stress than nickel in human A549 lung adenocarcinoma cells [233]. The oxidative stress induced by nickel is aggravated by specific mechanisms including the displacement of iron from the catalytic site of dioxygenase enzymes, which on one hand induces hypoxia and adding to oxidative stress, but on the other hand deregulates demethylase epigenetic enzymes (KDMs, TETs). Arsenic also generates ROS and depletes GSH availability by binding to thiol groups, together with the addition of its metabolic transformation into methylated forms. Further, arsenic displaces essential metals such as Zn from Zn finger proteins, it creates imbalances in histone variants and in global and site-specific changes in DNA methylation and HPTM. This series of epigenetic mechanisms and others are addressed in this chapter and summarised in Table 2.

### 4.1. Arsenicals

Arsenic (As) is an IARC class-1 human carcinogen and exposure to arsenic has been found to be related to the development of certain cancers, including skin, lung, bladder, and liver cancers [272]. The carcinogenic potential of arsenic is attributed to genotoxicity and to disturbances of epigenetic mechanisms [234,235]. Despite the abundant literature on the toxicity of arsenic [273,274,275] and the existing regulatory measures taken by numerous agencies (e.g., maximum drinking water contamination level of 10 µg/L (10 ppb or 0.13 µM) set by the World Health Organization and US-EPA [276]), the toxicity of this metal is still an important risk assessment research topic. This is because: (1) it is present naturally in the environment and as waste in mining industries [277], (2) inorganic arsenic levels exceed safe limits in drinking water in more than 50 countries [278], (3) reasons for inter-individual disease susceptibility to arsenic must be understood [278], (4) mitigation [278] or remediation nutritional supports are required for the exposed population [273], and (5), understanding the extent of genotoxic and non-genotoxic mechanisms contributing to its carcinogenic mode of action is important given that this might influence regulatory measures and treatment intervention for correctable non-genotoxic epigenetic anomalies. Finally, numerous epigenetic anomalies described in the following paragraphs and in the Supplementary Tables S2 and S3 occur at physiologically relevant concentrations (human whole-blood levels of total arsenic range from 0 to 46.5 µg/L or 0.6 µM) [279].

There is an ongoing debate related to the genotoxic or non-genotoxic carcinogenic nature of arsenicals. Roy et al. [280] concluded that inorganic arsenic is a genotoxic human carcinogen based on induction of chromosomal aberrations, sister chromatid exchanges, and micronuclei formation in human cells in vitro and in vivo. In contrast, others have indicated that arsenicals are non-genotoxic as they do not react with DNA, and that the genotoxicity originates from cytotoxicity and is therefore not the basis for cancer development [281]. Arsenic was reported to be only weakly mutagenic [235] and not a classical point mutagen [245,282]. Instead, arsenic was reported as an epimutagen [239,283], given the induction of deregulation of the epigenetic system leading to genomic instability (chromosomal and microsatellite instabilities, mutations, and micronuclei) [245,284,285]. In humans, arsenic exposure through drinking water induces persistent GGD hypomethylation and differentially methylated regions across generations with specific methylation patterns in individuals bearing arsenicosis skin lesions [237]. Arsenicals clearly disturb the epigenetic system by altering numerous mechanisms (Table 2).

#### 4.1.1. Oxidative Stress

Oxidative stress is a key mediator of arsenic-induced toxicity [286]. Although exposure to other metals (e.g., lead (Pb)) can also elicit oxidative stress [287], they do not have similar epigenetic effects or carcinogenic potential. The combination of oxidative stress induction and the metabolism of arsenicals limits the availability of SAM for DNMT and other MT [233,234]. Briefly, the metabolic transformation of absorbed pentavalent arsenate (As^+5^) produces arsenite (As^+3^) which is then mono-methylated (MMA) and di-methylated (DMA) into excretable metabolites (in line with the following reactions: As^+5^ + 2e → As^+3^ + Me^+^ → MMA^+5^ + 2e → MMA^+3^ + Me^+^ → DMA^+5^ + 2e → DMA^+3^). These metabolic reactions are conducted by enzymes (arsenic (+3) methyltransferases (AS3MT), glutathione *S*-transferase Ω (GSTO)) that are dependent upon SAM and GSH as co-factors. In addition, arsenite reacts spontaneously with sulfhydryls in cysteine residues of proteins and of GSH, so the combination of these mechanisms reduces GSH availability. Oxidative stress and GSH depletion stimulate redirecting homocysteine to the *trans*-sulfuration pathway for the synthesis of GSH, thereby reducing homocysteine availability for the methionine cycle that produce SAM (the required co-factor for DNMT and other MT) [234,235]. Therefore, collectively, the SAM- and GSH-dependent AS3MT activity, the interaction with GSH-sulfhydryls moieties, the induction of oxidative stress and the trans-sulfuration pathway, are all arsenic-induced mechanisms limiting the availability of SAM for MT [234].

#### 4.1.2. DNMT Expression and Activities

Arsenical exposures in human cells reduce DNMT expression and interfere with DNMT activities. Nuclear extracts from HepG2 cells exposed to 2 to 10 µM As_2_O_3_ for 48 h, incubated in the presence of polydeoxyinosine-deoxycytosine as substrate and tritiated-SAM as the methyl donor (analyzed by scintillation counting), showed a concentration–response reduction in DNMT activities associated with a reduction in DNMT1 mRNA [288]. The reduction in DNMT activities can be interpreted as a consequence of direct binding of arsenic to sulfhydryl moieties and/or reduction in gene expression [234]. Arsenic-induced expression reductions in DNMT1 and DNMT3A, as well as in SAM abundance, were observed in the human HaCaT cell line (a spontaneously transformed aneuploid immortal keratinocyte cell line from adult human skin) [236]. Arsenic trioxide used in chemotherapy [289,290] was shown to induce DNA hypomethylation in a human colon cancer reporter system at 50 and 10 µM after 24 h or 72 h, respectively [291]. In the human bronchial epithelial SV-40 immortalised cell line (BEAS-2B) exposed to arsenic for 8 weeks, reduction in mRNA and protein expressions of DNMT1, 3A, and 3B, were associated to a reduction in CTCF binding-protein on the DNMT promoters [240]. CTCF can have multiple roles acting as repressor, insulator, or as a transcription factor (TF), which in the latter case acts on promoters to favour gene expression. CTCF has 11 zinc fingers among which the 11th (the only zinc finger with a grouping of three cysteines and one histidine compatible with arsenic binding) was proposed to be inhibited by arsenic, and to contribute to a 64–67% reduction in CTCF binding and reduction in DNMT expression [240]. Collectively, the above in vitro data from different cell lines suggest that both short (24–48 h, 2 to 25 µM As) and long exposure (0.5 µM for 8 weeks) to arsenicals can disrupt DNMT activities via a variety of mechanisms such as interference with mRNA expression, CTCF, and protein activities [240].

#### 4.1.3. Ten Eleven Translocation (TET) Enzymes

Arsenicals can alter the activity of the TET enzymes that oxidize 5mC into 5hmC. Global genome reduction in 5hmC abundance and TET enzyme activities are frequently observed in human cancers due to mutation, to decrease in TET expression, or to decrease in co-factor availability [292]. However, effects of arsenic differ across models depending on stage of carcinogenesis and association of TET to different protein complexes. Owing to its ability to bind sulfhydryl groups of cysteine residues, arsenic can displace zinc ions from the zinc finger DNA binding domains of numerous proteins and potentially altering their functions (e.g., TIP60 histone acetyltransferase, RBX1 E3 Ubiquitin ligase, ERα, DNA repair proteins, PARP-1 [241]). Short-term exposure to arsenic (As^3+^ in the form of NaAsO_2_, 24 h, at 1, 2, 5 µM) was found to induce a concentration-dependent decrease in TET activities in human embryonic kidney HEK293T cells transfected with the catalytic domain of human TET1, TET2, and TET3 enzymes, and to reduce TET activities in mouse embryonic stem cells [190]. In contrast, long-term experiments in the human bronchial epithelial BEAS-2B cell line transformed with Ad12-SV40 2B (0.5 µM NaAsO_2_, 8 weeks) induced epithelial mesenchymal transition associated with increases in expression and activity of TET enzymes modulated by CTCF binding to the distal rather than the proximal *Tet1* and *Tet2* promoters [191]. This effect on TET enzymes leads to a global increase in 5hmC and genome-wide redistribution of 5hmC. It should be noted that while arsenic favored CTCF binding to the distal *Tet* promoters [191], it prevented CTCF to bind to *Dnmt1/3a/3b* promoters [240]. 5hmC is a demethylation intermediate and this increase in 5hmC precedes the usual global genome hypomethylation observed in cancers. Overall, the data described above and in Table 2 highlights differences in outcomes depending on the investigated models, while short-term experiments can reveal potentials for enzymatic disturbances, the long-term experiment with increased TET activities and epithelial mesenchymal transition does reveal a carcinogenic association.

#### 4.1.4. Micro RNAs

Arsenicals induce dysregulation of micro RNA (miR/miRNAs) expression. Cardoso et al. [242] reviewed human cell line studies describing arsenic-induced dose- and time-dependent changes in the expression of miR-21 (in skin HaCaT, in lung HBE, BEAS-2B, and in fetal hepatocyte L-02 cells), miR-141 (in HaCaT cells), miR-155 (in 16-HBE cells), miR-191 (in L-02 cells), miR-199a (in BEAS-2B cells), miR-200 family (in HaCaT and HUC1 cells), and the let-7 miR family in HaCaT cells. miR are translational repressors and do not generally modify the chromatin, but miR expression can be regulated by DNAm, as suggested for the let-7 family members [243]. Let-7 family members repress the oncogene *Ras* which is an upstream regulator of the NF-κB pathway involved in the acquisition of cancer stem cell-like properties. It was observed that short-term exposure to sodium arsenite (24 h, 1.0 µM NaAsO_2_) reduces the expression of Let-7 through a DNAm process that leads to activation of the RAS/NF-κB pathway and HaCaT cell transformation [243]. Using long-term chronic exposure to NaAsO_2_ (2.5 µM for 16 weeks) in p53^low^ human bronchial epithelial cells, or 20 ppm in drinking water from gestation day 18 until 34 weeks in heterozygous knockout p53^+/−^ or in ZEB1^+/−^ mice, Wang and Yang [293] summarize that DNAm-dependent decrease in expression of miR-200 lead to the upregulation of ZEB1 (zinc finger E-box-binding homeobox factor) promoting epithelial to mesenchymal transition (EMT) and lung tumorigenesis (ZEB1 is also upregulated by nickel, see next section). EMT is a process by which epithelial cells lose their cell polarity and cell–cell adhesion and gain migratory and invasive phenotypes characterized by genetic and epigenetic reprograming of multiple functions, including extra-cellular matrix organization [294,295] (also Section 9.4). Therefore, miR expression frequently deregulated by DNAm anomalies might be useful surrogate markers of epigenetic changes associated with carcinogenesis.

#### 4.1.5. Histone Variants

The mechanisms by which histones play important roles in carcinogenesis include (1) mutations which give rise to oncohistones [296], (2) histone post-translational modifications, and (3) abnormal expression of histone variants. Arsenic exposure was found to create a carcinogenic imbalance in the expression of the replication-dependent canonical H3.1 and non-canonical H3.3 variant [244]. Briefly, normally H3.1 mRNA expression is regulated by the protein SLBP (a stem-looping binding protein required for canonical histone mRNA processing and translation) and not by polyadenylation. However, arsenic exposure (BEAS-2B cell line, sodium arsenite NaAsO_2_, 0.5 and 1 µM, 96 h) prevents SLBP expression, leading to abnormal polyadenylation of H3.1 mRNAand its protein expression. H3.1 can then displace the H3.3 variant from gene regulatory elements (TSS, promoters, enhancers), which induces deregulation in gene pathways, chromosomal instability, cell transformation, and tumor formation in nude mice [244]. Further investigation of SLBP and H3.1 expression in the context of testing strategies is warranted for the identification of non-genotoxic carcinogens. Indeed, Chen et al. [244] also reported that nickel induces SLBP depletion and that this mechanism might be specific to arsenic and nickel.

Other histone variants are also involved in carcinogenesis which complicates epigenetic carcinogenic mechanisms. There are multiple H1 variants and normally the H1.0 levels accumulate in somatic cells gradually replacing the replication-dependent H1 variants. However, the expression of H1.0 is reversible and silenced in cancer cells through enhancer DNA methylation. Silencing H1.0 destabilises nucleosome-DNA interaction, induces derepressions of large sets of genes that maintain proliferative capacity and prevent differentiation [297]. Chemically induced H1.0 silencing remains to be investigated.

#### 4.1.6. Histone Post-Translational Modifications (HPTM)

Recent detailed reviews describe arsenic-induced HPTM associated with oxidative stress, DNA damage and repair, and cell cycle regulation [245,298]. In this section, these are discussed as well as other observations (see details in the Supplementary Tables S2 and S3).

For screening purposes, short-term experiments can detect alterations in histone methyltransferase (HMT) or histone demethylase (HDM) activities. For example, exposure to arsenic alters methyltransferase activities toward H3K27 in BEAS-2B cells [299], and H3K4 in the human lung carcinoma A549 cell line [300]. These experiments suggest that chemical-induced changes in methylated histones can detect alteration in HMT or HDM activities, but these changes may also reflect toxicity or adaptive mechanisms as well as, or instead of, carcinogenicity.

Others investigated effects on histone acetylation. Concentration and time response in the human urothelial UROtsa cell line (SV40 transformed non-tumorigenic) treated with sodium arsenite or monomethylarsonous acid (MMA^+3^) showed decreases in H4K16ac abundance after 24 h, presumably through reduction in activities of the histone acetyl transferase MYST1 [301]. Physical interaction between arsenic and the zinc finger domain (Cys2HisCys) of MYST1 (alias hMOF, KAT8) explained reduction in this HAT activity in the human embryonic kidney HEK293T cell line [302]. However, effects on H4K16ac appear to be cell type specific, increasing with p53 and the deacetylase SIRT1 within 24 h (0.5 µM As^+3^) in primary cultures of normal human epithelial keratinocytes (NHEK), and H4K16ac remained elevated until at least 48 days of culture [303].

The above NHEK cell study [303] revealing H4K16ac as an interesting epigenetic mark also confirmed a non-histone feedback loop between p53, SIRT1, and miR-34a in the regulation of p53 activity. Acetylation activates the p53 protein whereas the deacetylase SIRT1 maintains p53 inactivity; under “cellular stress” miR-34a downregulates SIRT1 which permits p53 activation. This p53/SIRT1/miR-34a axis is supported in the NHEK primary culture by a twofold increase in miR-34a after 24 h exposure to 0.5 µM As^+3^, but this increase was not detected in the HaCat cells and thus this loop was not confirmed in this cell line [303].

Long-term exposure experiments more directly imply links between arsenic exposure, HPTM, and carcinogenesis. In the UROtsa cell line, 8-week exposure to 50 nM MMA^+3^ caused global histone acetylation deregulation, changes in gene expression regulatory network, and malignant transformation after 12 weeks based on colony formation in agar and colony transplants developing into tumour xenografts in nude mice [304]. Using LC–MS/MS analyses over periods of exposure of 4, 8, 10, 12, and 14 weeks, the same laboratory reported decreases in acetylated H4 that remain relatively constant, but with dynamic changes in the series of acetylated H3 with highest abundance of H3K4ac and H3K23ac at the time of malignant transformation (8–10 weeks) [305]. They also showed a loss of H3K4me1, increases in H3K9me1 and H3K27me1, after 8 weeks of exposure [305]. The MMA^+3^-induced cell malignant transformation was blocked by exposure to a histone deacetylase inhibitor (suberoylanilide hydroxamic acid, SAHA) supporting a driver role of acetylation in this carcinogenic process [306]. This laboratory also found that H3K18ac was significantly increased in leukocytes of people exposed to high inorganic arsenic (165 μg/L) while H4K8ac was substantially decreased, and this supports their in vitro findings (H4 hypo and H3 hyperacetylation) [305].

The dynamic changes in histone acetylation described above [305], and differences in histone methylation reported in various studies (Table S2), demonstrate the need for documenting multiple acetylated and methylated histone marks in time-series experiments in order to derive a better understanding of the most relevant time point and HPTM that can be measured in the global genome to predict chemical carcinogenicity. However, global genome measures do not reflect gene-specific effects, such as DNA repair gene silencing.

#### 4.1.7. DNA Repair

The West Bengal population (30 million people) are exposed to arsenic through ground water and 15–20% of the individuals have arsenicosis (dermatological anomalies) [307]. Epigenetic analyses of peripheral blood mononuclear cells (PBMC) suggest that the DNA repair system is defective in the exposed population (urinary arsenic up to 434.1 µg/L compared to control with concentrations up to 33.7 µg/L) with arsenicosis symptoms [308]. Subjects with arsenicosis had increased activity and expression of the histone methyltransferase DOT1L (KMT4) as well as its product H3K79me1 and had reduced abundance of the tumour suppressor protein 53BP1 [308]. Bhattacharjee’s laboratory demonstrated that the components of the mismatch DNA repair system (MMR) are deregulated [246]. The MMR system components (i.e., MLH1, MSH2, MSH6 and PMS2) recognise mismatches and small insertions/deletions, and this is important for the protection of the genome against microsatellite and genomic instability. During DNA repair, normally MLH1 forms a heterodimer with PMS2, while MSH2 forms a complex with MSH6, and H3K36me3 recruit MSH6 to the chromatin anomalies. The authors demonstrated reduced expression and promoter methylation of the MMR genes MLH1 and MSH2, and reduced abundance of H3K36me3 which support susceptibility to DNA damage and cancer risks in arsenic-exposed individuals [246].

Recently, using in vitro experiments, Tryndyak et al. [309] demonstrated in human liver HepaRG cells that the concentration of sodium arsenite (1 µM, NaAsO_2_, 14 days) found naturally in contaminated water decreases H3K36me3 abundance by 54%. Additionally, genome-wide DNA demethylation and site-specific DNA hypermethylation, changes in gene expression supporting epithelial-to-mesenchymal transition, inhibition of DNA repair genes, oxidative damage of proteins, DNA damage, and genomic instability arise. They concluded that the interplay between both the epigenetics and genetic alterations contributed to arsenicals-induced carcinogenesis. Based on these observations and on H3K36me3 that can promote MMR [245] and homologous recombination repair [310], it appears that H3K36me3 might be a suitable predictive biomarker to elucidate both initiations as well as promotion mechanisms.

In addition to H3K36me3, in vitro experiments suggest beneficial roles for H3K79me1 and H4K20me2/3, but detrimental roles for H3K9me2 in DNA repair. Under in vitro conditions, sodium arsenite (≥0.4 µM As^+3^) increased the abundance of H3K79me1 [308]. A role of H3K79me1 in the recruitment of 53BP1 to sites of DNA damage has been suggested, but others assigned DNA repair function to H4K20me2 [311], which can bind 53BP1 to enhance nonhomologous repair [312]. H3K9me2 abundance measured throughout the genome of HaCat cells displays a downward sodium arsenite concentration–response pattern, but using Chip-seq it increased the abundance of H3K9me2 in promoters of base excision repair genes (*Mpg*, *Xrcc1*, and *Parp1*), silenced their expression and aggravated DNA damage [247]. As an additional mechanism, Paul and Giri [283] hypothesize that an increase in G9a activity (the histone methyltransferase required for H3K9 dimethylation) not only increases the H3K9me2 moiety causing downregulation of tumour suppressor and DNA repair genes but also might inactivate p53 via methylation of p53^K373me2^ that hinder the p53-dependent DNA repair pathway. Finally, sodium arsenite exposure was shown to increase BRCA1 promoter methylation in a human breast cancer cell line in vitro [313], also reducing DNA repair capacity (see Section 3.1).

#### 4.1.8. Mitochondrial Biogenesis

Mitochondrial DNA content and mitochondrial mass both increase during the transition from normal tissue to hyperplasia and malignancy, as indicated by Lamb et al. [314] investigation using human breast cancer MCF7 cells and normal and cancer cells from various tissues. Similarly, chronic exposure to arsenic increases mitochondrial DNA copy number, mitochondrial toxicity, and oxidative stress in PBMC [249] and arsenic-induced skin cancers [248]. Epigenetic alterations play crucial roles linking arsenic-induced mitochondrial biogenesis and arsenical skin carcinogenesis. The mitochondrial DNA displacement loop (D-loop) controls the transcription of the mitochondrial genome including the gene *Nd6* (Nicotinamide adenine dinucleotide subunit 6, the only mt gene present in the heavy chain of mtDNA). Hypomethylation of both the D-loop and *Nd6*, as well as expression of ND6, increases with the urinary level of arsenic [249]. The mitochondrial biogenesis functions of the D-loop are regulated by the transcription factor TFAM (Transcription Factor A, Mitochondrial), which is transcribed from its nuclear gene, and also hypomethylated and expressed in PBMC in arsenic exposed populations and in arsenic-induced skin tumours [248]. Finally, miR-663 is a mediator of mitochondrial oxidative phosphorylation and has tumour suppressor functions, its promoter was found to be hypermethylated and its expression found to be decreased in arsenic skin cancer tissues compared to noncancerous control tissue [248]. Collectively, this information suggests that deregulation of epigenetic mechanisms in mitochondrial biogenesis (and mitochondrial DNA copy number) is induced by arsenic exposure and in arsenic-induced skin cancer.

#### 4.1.9. In Utero Exposure

In utero exposure to arsenic has been reported to modify the fetal epigenetic system which then contributes to cancer and disease risks later in life. In utero and childhood exposure to arsenic has been associated with increased mortality from lung, bladder, liver, and laryngeal cancers, and chronic renal diseases in adulthood [315]. In vivo rodent studies show that transplacental exposures to arsenicals during pregnancy carry a higher cancer risk compared to exposures in adulthood, with sex, strain, and tissue differences in tumour incidences [316].

Links between in utero exposure and abnormal DNAm were demonstrated in both human and animal investigations. For example, in a Mexican population exposed via drinking water (0.456–236 µg/L), newborn cord blood leukocytes demonstrated 2919 genes with differentially methylated regions, among which a subset of genes with altered DNAm levels was associated with gene expression as well as birth outcomes, including newborn gestational age [317]. More recently, water and maternal toenail arsenic concentrations in a Bangladesh population were associated with decreases in gestational age and birth weight, the latter correlating with increases in *DNMT3A* CpG methylation [318] and decreases in methylation of miR-124-3 in cord blood samples [319]. In C3H mice, the livers of male fetuses at 19 days of gestation from dams exposed to arsenic from gestation day 8–19 (42.5 and 85 ppm in drinking water, doses known to be carcinogenic but not acutely toxic) demonstrated global DNA hypomethylation, gene-specific hypo and hypermethylation (e.g., *Cyclin D1*, *Tp53*), and 140 aberrantly expressed miRNAs [320]. Further research is needed to understand effects of transplacental transfer of arsenic on fetal epigenetic programming and disease outcomes in adulthood.

#### 4.1.10. Human Blood Measurements Confounding Factors

Sources of variability in human studies may be affected by differences in the cellular composition of samples, differences in metabolism, nutrition, and assay procedures. Bozack et al. [238] conducted an epigenome-wide association study (EWAS) to investigate the relationship between low to moderate levels of arsenic exposure and loci-specific DNAm in blood samples of a large population of American Indian adults (*n* = 2325 individuals). Twenty CpG sites mostly hypermethylated were associated with urinary arsenic levels, and were located in genes (*Slc7a11*, *Anks3*, *Lingo3*, *Csnk1d*, *Adamtsl4*) that together may be involved with GSH biosynthesis, tumor development, and glucose metabolism. In addition, they found a differentially methylated region on chromosome 11 annotated to the genes *C11orf2* and *Tspan32*, located in a cluster of imprinted genes known to be involved with the Beckwith-Widemann syndrome (abnormal growth and tumors in childhood) and chronic lymphocytic leukemia. Bozack et al. [238] reported similarities and discrepancies across similar EWAS studies and highlighted factors to explain the differences, including the cellular composition of the peripheral blood leukocytes population. Each leukocyte cell type has specific patterns of epigenetic marks, and they found an increased proportion of NK cells but a reduced proportion of B cells with urinary arsenic levels; such proportions may vary across studies and exposure levels [238].

There are sex differences in the one-carbon metabolism pathway generating SAM necessary to methylate arsenicals [321], such that men are at a higher risk of developing skin lesions due to the lower methylation rate of MMA to DMA [322]. Differences in arsenic metabolism, nutrition and micronutrients (e.g., methionine, choline, folate, betaine, and vitamin B12) are known to affect DNAm [273,321]. Timing of sampling relative to exposure and disease status may also influence detection of associations. Positive dose-dependent associations between arsenic exposure and peripheral blood leukocyte DNAm were found following occupational exposure to arsenic [323,324], and in a Bangladesh population [325]. This has been suggested to be adaptive because hypomethylation of leukocyte DNA was associated with increased risk for skin lesions [326]. Similarly, arsenic exposure in human populations generally leads to Line-1 hypomethylation [239]; in this case, methylation in retrotransposons is provided as an indicator of global genome DNA methylation owing to their genome-wide distribution. The data produced from different methodologies can be difficult to compare, for example Line-1 DNA methylation does not always correlate with global genome changes. In the above study, global genome DNA methylation was measured using commercially available antibody driven colorimetric ELISA assays [323,324], or radiometric ^3^H-methyl incorporation assay [326].

There are also challenges in reproducing patterns of histone modifications across epidemiological investigations. Global genome histone marks (H3K4me3, H3K9ac, and H3K9me2) are stable over time in PBMC within individuals, supporting their use in epidemiological investigation [327]. Howe and Gamble [298] reviewed studies suggesting that arsenic increased abundance of H3K9me2, H3S10ph, but losses of H4K16ac. In contrast to global loss of H4K16ac, which is reported as a hallmark of human cancers, findings for other HPTM have not been entirely consistent across studies; the authors attributed differences in the dose, duration, exposure timing, type of arsenic species examined, the tissue or cell line evaluated, differences by sex, factors affecting the capacity to metabolise arsenic, induction of histone variants carrying different HPTM and methodological issues [298].

### 4.2. Nickel

IARC classified nickel (Ni) compounds as carcinogenic to humans (Group 1) [328]. They reported an elevated risk of lung and nasal sinus cancers among Ni refinery workers. The direct interaction of nickel compounds with DNA does not appear to be the cause for initiating the carcinogenic response; the induction of oxidative DNA damage, chromosomal damage, and weak mutagenicity, were associated with several indirect mechanisms including oxidative stress, inhibition of DNA repair, and epigenetic mechanisms [328]. Both types of genomic effects (genotoxic and epigenetic) have been reported, for example, nickel (NiCl2, 0.25 mM for 18 h) was found to be a potent inducer of Syrian hamster dermal cell immortalization [329], whereby DNA damage and epigenetic disruption were revealed through loss of a p16 (*Cdkn2a*) allele, and DNA hypermethylation of the p16 (*Cdkn2a*) promoter on the remaining allele [251]. Exposure to Ni disrupts epigenetic marks and gene expression through multiple ways—by preventing CTCF from binding to DNA [262,263], by favouring DNA methylation [253] and chromatin condensation [330], by inhibiting dioxygenases [256], by increasing abundance of H3K9me2 [254], and by spreading heterochromatin marks to euchromatin. Heterochromatinization of chromatin inhibits molecular interactions with the underlying gene’s sequence and effectively induces gene silencing [330].

The type of Ni compounds and cell types used as a model can influence prediction of toxicity from screening assays. Broday et al. [250] examined the effects of soluble (NiCl_2_) and insoluble (crystalline subsulfide, Ni_3_S_2_) nickel, and found that NiCl_2_ induced H4 hypoacetylation in yeast (0.2 mM); however, in human A549 cells (lung adenocarcinoma), NiCl_2_ had no effect but the subsulfide did induce H4 hypoacetylation after a 2-day exposure at 0.2 µg/cm^2^. There is sufficient evidence for the carcinogenicity of nickel sulfides, including nickel subsulfide, but limited evidence for nickel chloride [328]. Moreover, the above data showing an effect of NiCl_2_ in yeast but not in human A549 cells demonstrates the importance of and preference for data derived from human cells in human epigenetics.

#### 4.2.1. Heterochromatization and H3K9 Methylation

Mechanisms for nickel-induced gene silencing have been investigated in a number of publications from Costa’s laboratory [254]. Using a Chinese hamster cell line devoid of the endogenous hypoxanthine guanine phosphoribosyltransferase but transfected with the bacterial xanthine guanine phosphoribosyltransferase (*gtp)* gene inserted near a heterochromatin region, it was demonstrated that Ni induces a redistribution of the H3K9me2 and 5mC silencing marks. Ni had no effect when the transgene was not inserted near heterochromatin [254]. In this assay, 6-thioguanine is toxic to the cells when the gene *gtp* is active, but Ni epigenetically silences *gtp* and maintains cell survival. Ni (subsulfide at 2 µg/cm^2^ for 16 h in a 3-day culture [253]) inactivated the transcription of the transgene by inducing DNAm, DNA compaction, reduction in H4 acetylation, increases in the abundance of H3K9me2 by inhibition of the H3K9 demethylase [253,254]. The inhibition of the transgene was likely dependent upon DNAm since inhibition was reversed following treatment with the DNMT inhibitor 5-aza-2′-deoxycytidine [254]. The effect of Ni on global genomic abundance of H3K9me2 was also observed in human cell lines, such as HOS human osteosarcoma, HEK293 embryonic kidney, and A549 human lung adenocarcinoma cell lines. For example, in the A549 cell line, dose- and time-dependent increases in global H3K9me2 (but not me3) are induced by NiCl_2_ with a first effect detected after 12 h exposure to 0.25 mM [254]. The latter group also observed that NiCl_2_ can reduce the histone methyltransferase activities of G9a (alias euchromatic histone-lysine N-methyltransferase 2, EHMT2 or KMT1C, targeting H3K9 dimethylation) and SUV39H1 (alias KMT1A, targeting H3K9 trimethylation) [254]. It should be noted that these observations of Ni-induced reduction in G9a and SUV39H1 activities considered independently would have been misleading by suggesting a reduction in histone methylation, but in fact the final outcome is gene silencing through increases in H3K9me2. Therefore, the presence of dual mechanisms in HPTM (in this case, methylation and demethylation) favours the use of screening assays that measure the epigenetic mark in a complete cellular system rather than isolated enzymatic activities.

#### 4.2.2. Inhibition of Dioxygenases

The previous paragraphs highlight H3K9me2 abundance and histone demethylase activity as relevant epigenetic endpoints in Ni carcinogenicity. Lysine demethylase 3A (KDM3A/JMJD1A) demethylates H3K9me1 and me2, while KDM4A-D/JMJD2A-D acts on H3K9me2 and me3 [331,332]. As discussed in Section 2.3, most demethylases belong to families of iron and 2-oxoglutarate-dependent dioxygenases that share two histidines and a carboxylate as motif to coordinate the Fe^2+^ ion at the catalytic site. The Ni ion can replace iron at the catalytic site and inactivate the Fe^2+^ and 2-oxoglutarate-dependent dioxygenases [255]. In addition to inhibiting KDM3A, Ni can inhibit other dioxygenases including the hypoxia-inducible factor (HIF) prolyl hydroxylase dioxygenase (PHD), the DNA repair enzyme ABH2 that demethylates alkylated DNA bases (1-MeA, 3-MeC, 1-MeG, and 3MeT), and TET DNA demethylases [71,255]. The affinity of Ni^2+^ for the catalytic sites of TET1, PHD2, and KDM4A-D, is 7.5-, 4.5-, and 4-fold higher than that of Fe^2+^, respectively [259], suggesting persistent disruption of iron binding even after discontinuation of exposure [255].

The consequences of KDM inhibition were investigated further by Chen and collaborators [333]. They found that Ni-induced inhibition of KDM3A increases H3K9me2 in promoters of 68 KDM3A-target genes in human bronchial epithelial BEAS-2B cells. Among these, the gene *Spry2* was found to be downregulated by hypoxia (1% O_2_ for 3 days) or exposure to NiCl_2_ (100 µM, for 3 to 8 weeks) via increased abundance of H3K9me2 in the promoter. SPRY2 is a negative regulator of the receptor tyrosine kinase-extracellular signal-regulated kinase signaling (RTK-ERK) pathway. The effect on SPRY2 expression was not reversed by 5-aza-2′deoxycytidine treatment and so considered to be independent from DNAm. The repression of *Spry2* can potentiate Ni-induced anchorage-independent growth of BEAS-2B colonies in soft-agar, which is an indication of a carcinogenic cell transformation step [333]. It is interesting to note that the loss of KDM3A activity was associated with its increased expression induced by HIF-1α binding to its promoter, a mechanism explained by the Ni-induced inhibition of the PHD which stabilizes the transcription factor HIF-1α, that then activates hypoxia gene expression pathways [333].

#### 4.2.3. Hypoxia and Oxidative Stress

Important mechanisms of Ni-mediated carcinogenesis involve its ability to mimic hypoxia by replacing iron from the PHD dioxygenases [257,258], and induction of oxidative stress that further reduces dioxygenase activities by preventing the reduction of Fe(III). Fe (III) present in inactive dioxygenase enzymes is reduced to Fe(II) following electron donation from ascorbate and this then permits enzyme activation. ROS compete with Fe(III) as electron acceptors and this prevents the regeneration of active enzymes. Conditions creating hypoxia can induce oxidative stress even in the absence of chemical treatment. For example, in human pancreatic cells hypoxia stimulates TGFB1-induced NADPH oxidase 4 (NOX4) expression and activity, this is a mitochondrial-independent source of ROS. The latter generates oxidative stress and inactivates KDM5A leading to increases in H3K4me3, in particular in the promoter of *Snail1* (a key regulator of epithelial-mesenchymal transition (EMT)) which increases its expression and that of EMT [334]. Culture systems are usually deficient in reproducing the appropriate in vivo oxygen tension and antioxidant balance to affect the epigenetic system even in the absence of chemical treatment. Induction of oxidative stress by H_2_O_2_ treatment was shown to increase abundance of methylated histones (short-term 3 h H3K4me3; long-term 3 weeks H3K4me3 and H3K27me3) by decreasing KDM activities, and to also decrease activities of the dioxygenase TETs DNA demethylases [71]. These results support KDM dependence on ascorbic acid as an antioxidant, which, when supplemented, decreased the abundance of H3K9me2 to approximately 30% of the control levels, regardless of the H_2_O_2_ treatment. The short-term H_2_O_2_ treatment also decreased abundance of the acetylation marks H3K9ac and H4K8ac that could be related to small increases in activities of class I/II histone deacetylases. These H_2_O_2_ effects can be adaptive given that global methylation and acetylation changes did not persist after 3 weeks [71]. Ni-induced hypoacetylation is not limited to H3 and H4; it also occurs in H2A and H2B, with H2B being the most sensitive, on the basis of time- and concentration-dependent experiments performed with the human airway epithelial 1HAEo cell line (initial 20% reduction after 12 h at 0.4 mM of Ni^2+^ acetate with detectable reduction at 0.1 mM at day 5 [251]). Overall, these results demonstrate the vulnerability of the epigenetic system to chemicals and conditions inducing oxidative stress or hypoxic conditions.

#### 4.2.4. Ubiquitination/Deubiquitination Machinery

Ubiquitination of proteins can lead to protein degradation by proteasomes [335], which for example, regulate cell cycle progression and tumorigenesis by the degradation of cyclin partners and cyclin kinase inhibitors [336]. Ni-acetate was shown to deregulate the ubiquitination/deubiquitination machinery acting on core histones H2A and H2B, with the potential to alter gene expression and genomic integrity [260,261]. Briefly, time and concentration-dependent increases in abundance of H2A and H2B ubiquitination have been observed in many cell lines (up to 72 h exposure to soluble (≤1 mM) or insoluble (≤1 µg/cm^2^) Ni; cell lines: A549, Cl41, Beas-2B, HeLa and Hep3B); however, it was suggested that the increases were due to the inhibition of deubiquitinating enzymes [261]. Others report that exposure of the human pulmonary cell line HPL1D with Ni-acetate (Ni(II), 1 to 5 days, 0.05–0.5 mM) induces small changes on H2A but a gradual increase in H2B ubiquitination peaking at approximately 0.2 mM, followed by ubiquitination suppression that concurred with H2B truncation (a similar biphasic effect was observed for the abundance of H3K4me2 which increased up to 0.1 mM followed by a decrease below control level) [260]. Abundance of some ubiquitinating enzymes was also affected (RAD6 and UBCH6). RAD6 is an integral part of the post-replication DNA repair systems, which conjugates ubiquitin with histones H2B and H2A whereas UBCH6 conjugates on H2B. A steady decrease in RAD6 level with increasing Ni(II) concentration was observed, but UBCH6 levels tended to increase. The biphasic effects of Ni require further investigation to better discriminate between adaptive and adverse effects, given that monoubiquitination of histone H2A that occurs on K119, is associated with gene silencing. However, monoubiquitination on H2B occurring on K120 (H2Bub1) decompacts chromatin, regulates nucleosome dynamics to allow DNA access, contributes to DNA replication, repair, and transcription, plays a role during meiosis, mitotic chromosome segregation, and in maintenance of centromere and telomere [337]. The loss of H2Bub1 is associated with cancers, not surprisingly given that its interactome includes p53, BRCA1, the SWI/SNF remodelling complex, and HMT DOT1L and COMPASS, and multiple deubiquitinases including USP22 and USP44 [338].

#### 4.2.5. Interference with the Zn^2+^ Finger Protein CTCF

As with arsenic and cadmium that can replace the Zn^2+^ ion from the zinc finger DNA binding domain of proteins [190,259], there are data suggesting that Ni can interfere with the Zn^2+^ finger protein CTCF. Based on electrophoretic mobility shift assays, Jose et al. [262] demonstrated concentration-dependent inhibition (NiCl_2_, 50 to 300 µM) of CTCF/DNA complex formation in vitro, with greater inhibition of DNA sites with weak affinity for CTCF. In the human BEAS-2B cell line, they also observed Ni-induced (NiCl_2_ at 500 µM for 72 h) spreading of the H3K9Me2 silencing mark from heterochromatin to euchromatin regions bound by CTCF binding sites. They suggested that in addition to a reduction in demethylase activity, reduction in CTCF DNA binding can promote spreading of H3K9Me2 and gene silencing.

#### 4.2.6. Long-Term Exposure and Persistent Effects in the Absence of Exposure

Long-term exposure to Ni has been demonstrated to induce persistent effects in human bronchial BEAS-2B and non-invasive RT4 human cancer cell lines, and these Ni-induced EMT changes and altered gene expression remained irreversible even after termination of exposure [188,263]. Indeed, long-term in vitro exposure of BEAS-2B cells to Ni (6 weeks at 10, 50, 100 µM NiCl_2_) induces persistent EMT as determined by the observations of RNA-Seq EMT enriched pathway, downregulation of epithelial markers (*E*-CADHERIN and CLAUDIN-1), upregulation of mesenchymal marker (fibronectin), wound healing and transwell invasion assays [263]. While short-term exposure to a high dose of Ni (72 h, 500 µM) revealed expression of EMT gene pathways, these short-term exposure changes did not persist [263].

A year later, the same group demonstrated a chronology of changes in gene expression profile (RNA-Seq, Illumina HiSeq 4000) across four groups of BEAS-2B cells: (1) untreated, (2) exposed to 100 μM NiCl_2_ for 6 weeks, (3) exposed and then washed and reseeded at clonal density and grown in Ni-free medium for two weeks, or (4) for 6 months [188]. The Ni treatment induced transient and persistent down- and upregulation of genes. The most abundant changes in gene expression were the persistent changes from group 4 representing treated washed cells reseeded at clonal density and grown for 6 months in Ni-free medium. This demonstrates that Ni exposure-induced genome-wide transcriptional changes can persist even after the termination of exposure, and interestingly that the transcriptional changes were much more abundant 6 months after the termination of exposure [188]. They also investigated changes in abundance of H3K4me3 and H3K27me3 in gene promoters (±2 kb from TSS) in the presence of Ni, and after the washout. Gene expression changes that occur in the presence of Ni were not associated with alterations in H3K4me3 abundance (an upregulation mark), but persistent increases in expression occurring after the washout period were associated with increases in H3K4me3. Genes with persistent downregulation showed no changes in H3K27me3 (a bivalent silencing) but genes upregulated were associated with decreased abundance of H3K27me3 both in the presence of Ni and after the termination of exposure [188].

Collectively, these results demonstrate the importance of long-term in vitro studies to understand persistent effects induced by chemicals and shed light on the importance of genomic location and timing for epigenetic changes associated with persistent effects in gene expression. Long-term 6-week exposure is a required delay for expressing Ni-induced EMT phenotypes and for distributing the heritable epigenetic marks across the cell population, whereas the 6-month period in Ni-free medium was sufficient for the selection over time of proliferating epigenetically different variants.

To gain a better understanding of the mechanisms leading to EMT, Jose et al. [263] demonstrated a decrease in H3K27me3 abundance in the promoter of the gene *Zn finger E-box Binding Homeobox 1* (ZEB1, an EMT master regulator), and the irreversible upregulation of ZEB1 (and downregulation of the ZEB1 repressors miR-200/205). ZEB1 recruits epigenetic regulators (SWI/SNF chromatin-remodeling complex) to its target genes, such as *Cdh1* (E-CADHERIN) [339], which are found downregulated by Ni exposure [263]. As an additional mechanism for EMT induction, others have shown that Ni-induced EMT in BEAS-2B cells (colony formation, anchorage-independent growth) was associated with increased expression of the stress-response protein Nuclear Protein-1 (NUPR1) mediated by JUN, FOS, and AP-1 activity [258]. Finally, Zhu and Costa [189] indicated that Ni-induced cell transformation is associated with expression modulation of non-coding RNAs, but these changes originate from Ni-induced activated DNMTs, HDACs, and HIF-1α pathways.

Overall, Ni can induce a redistribution of epigenetic marks (DNAm, HPTM). Some mechanisms may be similar to those of arsenic (oxidative stress, interfere with the Zn^2+^ finger protein CTCF), but Ni affects additional systems including deregulation of ubiquitination/deubiquitination, iron displacement from multiple dioxygenases including demethylases, hypoxia regulating enzymes, and DNA repair enzymes, which together modify various gene expression pathways conducive to EMT and carcinogenic steps. The importance of performing long-term experiments to allow for the manifestation of delayed effects following withdrawal of Ni exposure, and the importance of monitoring both methylase and demethylase activities to explain changes in abundance of a shared epigenetic target between enzymes, were clearly demonstrated.

### 4.3. Phenobarbital

IARC classified phenobarbital (PB) as possibly carcinogenic to humans (group 2B) [340]. PB is used as an anticonvulsant drug and has been extensively investigated as a non-genotoxic rodent hepatocarcinogen and epigenetic disruptor [264,341,342]. It is used as a prototype tumour inducer in projects such as MARCAR (https://www.imi.europa.eu/projects-results/project-factsheets/marcar, accessed on 5 October 2021) aiming at understanding and identifying early biomarkers of NGTxC. However, in humans, PB is not mitogenic and is not considered a liver carcinogen [341]. Human hepatocytes are refractory to the mitogenic effects of PB and CAR activators based on in vitro cultures as well as on chimeric mice models [343,344]. Nevertheless, PB investigations have revealed important epigenetic mechanisms that can assist in guiding human epigenetic data interpretation and in the development of epigenetic testing strategies. These epigenetic findings reported here, involve the stress protein GADD45 in mediating DNA demethylation, the relevance of 5hmC and of the *Dlk-Dio3* locus as potential markers of carcinogenesis.

#### 4.3.1. Mechanisms of Cell Proliferation and DNA Demethylation

It was recently reviewed that the effects of PB are mediated through indirect activation of the constitutive androstane receptor (CAR) [345]. PB does not bind within the CAR ligand binding domain and is known to modulate CAR activity by binding and reducing activity of the epidermal growth factor receptor (EGFR). This reduces the abundance of phosphorylated ERK1/2 and releases ERK1/2 from the inactive CAR homodimer. These events lead to further modification and heterodimerization of CAR to the retinoid X receptor (RXR), which can then activate target genes [345]. PB induces the expression of CYP2B enzymes, drug transporters, and proteins involved in lipid; alters glucose and energy homeostasis; and stimulates rodent liver cell proliferation [346].

Sharapova et al. [264] unravel a possible mechanism by which NGTxC could induce activation of DNA demethylase enzymes (through the same mediator of cell proliferation). They observed that the stress-response protein GADD45B (growth arrest and DNA damage-inducible 45 beta) was upregulated by both the PB and clofibrate treatments. First, this can explain the proliferative effect of these NGTxC. Normally, GADD45B indirectly interacts with p38 MAPK to activate p53, the cell cycle check point, and cell cycle arrest; however, PB activates CAR that binds and inhibits GADD45B as part of a complex that represses p38 MAPK signaling, and thus facilitates hepatocyte proliferation [347]. Secondly, GADD45 regulates DNA methylation/demethylation by interacting with TET1 (oxidizing 5mC to 5hmC) [90], thymine DNA glycosylase [91], and DNA repair complex [92,93]. Sharapova et al. [264] hypothesised that these mechanisms might contribute to the hypomethylation observed during chemical-induced carcinogenesis.

#### 4.3.2. 5-Hydroxymetylcytosine (5hmC) as an Early Marker of NGTxC

Correlations between 5hmC and 5mC have been extensively investigated in PB experiments. A causal role for PB-induced changes in DNA methylation (5mC) of cancer-related genes in mice was suggested quite some time ago [348,349]. Now, a change in abundance of 5hmC is suggested to be a more sensitive indicator of epigenetic anomalies than is 5mC, and perhaps an early marker of hepatocarcinogenesis [264]. Hydroxy-methylated DNA immunoprecipitation (HmeDIP) and MeDIP on microarray technologies demonstrated more abundant PB-induced changes in 5hmC than in 5mC, and a stronger correlation between gene expression and 5hmC than with 5mC [344,350,351]. It is notable that better immunogenicity of 5hmC than 5mC was suggested to partly explain these observations [344]. Upon 28-day exposure to 0.05% PB in drinking water in mice, strong correlations between increasing abundance of 5hmC and decreasing levels of 5mC in the promoters of highly induced genes were demonstrated, which were also associated with increases in H3K4me2 and H3K36me3 and decreases in H3K27me3 [350]. Changes in 5hmC and in 5mC are detected after only one day of PB treatment and increase over time (7, 28, and 91 day of exposure), before losses of both modifications in some proximal promoter regions following 91 day of exposure, in line with a role of 5hmC as a demethylation intermediate [351]. Ohara et al. [344] compared hepatocellular adenomas of liver samples from mice treated with a 90 mg/kg intraperitoneal injection of diethylnitrosamine followed by 500 ppm or to 0 ppm PB in the diet for 27 weeks. They observed that the major enrichment pathways detected by 5mC- and 5hmC-altered genes differ from pathways obtained based on altered gene expression; both enrichment results for 5mC and 5hmC profiles contain fewer pathways related to cell cycle regulation and more pathways related to development and cancer than gene expression. The resultant interpretation is that the activation of signaling pathways leading to tumorigenesis is more associated with DNA modification than with gene expression [344].

Sensitive techniques are required to accurately investigate and identify the tissue-specific low abundance of 5hmC (e.g., 0.03% of all cytosines in spleen, 0.7% in cerebral cortex), in contrast to the relatively more abundant and uniform amount of 5mC across tissues (4–5% of all cytosines) [352]. Liquid chromatography–ionization mass spectrometry (LC–MS) analyses of 5hmC and 5mC in liver samples from CbyB6F1-Tg(HRAS)2Jic^wt/wt^ mice (a transgenic mice bearing copies of the human *H-ras* protooncogene to expedite carcinogenicity assessment [353]) exposed to 0.5% clofibrate or 0.14% PB in the diet for 7 and 28 days were recently investigated [264]. PB and clofibrate are rodent NGTxC acting through CAR and PPARα [354], respectively. The abundance of 5hmC was decreased at 7 days for the PB, but at 28 days for clofibrate-treated mice, whereas 5mC was not affected by any treatment [264]. Moreover, they showed that a methyl donor supplemented diet (methionine, choline, folate, betaine, vitamin B12) can transiently counteract the adverse effects of PB at day 7, but not at day 28. Overall, decreases in 5hmC precede global DNA hypomethylation, so global loss of 5hmC has been proposed as an early marker of hepatocarcinogenesis [264]. After reviewing 5mC and 5hmC in vitro and in vivo data following exposure to pharmaceuticals and reagents (PB, diethylstilbestrol, cocaine, methamphetamine, ethanol, and dimethyl sulfoxide), and pollutants (heavy metals, particulate air pollution, bisphenol A, hydroquinone, and pentachlorophenol metabolites) in various cells and tissues, Efimova et al. [265] also reached a similar conclusion indicating that DNA hydroxymethylation is a sensitive biosensor that may play a role in mediating genome flexibility.

#### 4.3.3. NcRNAs from the *Dlk1-Dio3* Locus as Marker of Carcinogenesis and Epigenetic Deregulation

PB (≥0.02% in drinking water for 28 days [355]) as well as other CAR activators (including chlordane, gavage 8 mg/kg/day for 28 days [266]) were found to increase the expression of miRNAs and LncRNAs from the imprinted *Dlk1-Dio3* locus from mouse liver perivenous hepatocytes. Activation of this locus and expression of its ncRNAs (such as Meg3 [267]) lead to the proposition that this locus could serve as biomarker for non-genotoxic hepatocarcinogenesis [266,267]. The *Dlk1-Dio3* locus is an imprinted genomic region that includes three protein-coding genes (*Dlk1*, *Dio3*, and *Rtl1*) on the paternal allele, a cluster of microRNAs (≈53) and several long non-coding RNAs on the maternal allele [356]). This region is conserved among mammalian species, and it is located on chromosome 12qF1 in the mouse and 14q32.2 in humans [357]. It has been shown to be activated in the c-MET-driven mouse hepatocellular carcinoma model [357], and in the KRAS-induced lung adenocarcinoma mouse model [356]. DIO3 (iodothyronine deiodinase type III) has functional roles in a number of relevant canonical pathways [358] and in multiple human cancer subtypes [356,357,359,360,361]. The *Dlk1-Dio3* locus is subjected to epigenetic deregulation and was found hypomethylated but with a hypermethylated *Dio3* segment in lung tumours from smokers [360]. Activation of the *Dlk1-Dio3* locus is therefore not PB specific but it is a carcinogenic marker in many tissue types. *Dlk1-Dio3* is an imprinted locus, it controls the expression of many miRNAs, it is inducible by β-catenin, and the origin of its activation (epigenetics, transcription factor signaling, miR-122 [362]), including its chemical inducibility and specificity of its non-coding RNA expression, indicates that it deserves further investigation as a carcinogenic marker for chemical hazard assessment purposes.

#### 4.3.4. DNA Methylation Enzymes and L1 vs. Apical Endpoint

PB investigations highlight interesting comparisons between apical effects and doses inducing epigenetic changes. For example, following 7-day exposure to PB or to the hypolipidemic drug clofibrate in F344/DuCrl male rats, Miousse et al. [201] demonstrated that both chemicals increased cell proliferation (replicative DNA synthesis is the key event in the MOA of CAR activators [341]), reduced hepatic expression of epigenetic system components (clofibrate reduced DNMT1, DNMT3A, MECP2, and MBD1, whereas PB reduced DNMT3B and MBD1), and altered miRNA expression in the liver but not in the serum. Finally, only PB induced a statistically significant 4% decrease in DNAm of L*L1* open reading frame-1 (orf1), which occurred at the lowest dose previously demonstrated to be carcinogenic (100 mg/kg/day). Although there is to our knowledge no known DNA hypomethylation threshold for L*L1* activation to compare with the 4% decrease, DNA hypomethylation occurred at the potentially carcinogenic dose, eventhough it was not the most sensitive endpoint. Thus, in this short-term 7-day study, DNA hypomethylation at the carcinogenic dose may more accurately predict liver carcinogenesis than endpoints demonstrating adverse effects at lower doses [201]. In addition, the study by Miousse et al. [201] showed that changes in expression of genes from the DNAm machinery occurred only in the liver (the carcinogenic target), and not in the kidney.

### 4.4. Irradiation

Although irradiation induces DNA damage, numerous irradiation experiments highlight epigenetic deregulation [363], effects distant from target cells, and persistent effects that can inform about carcinogenic mechanisms for consideration in the development of testing strategies for NGTxC. GGDHo and genomic instability are induced by ionizing radiation in exposed cells [364], but also in cells distant from the irradiated sites with GGDHo that still persist 7 months after exposure [137,268]. Indirect effects in unexposed cells, referred to as field or bystander effects, and persistent effects in subsequent generations of unexposed cells, all imply active mechanisms. Indirect effects were demonstrated to be transferable to naïve cells (e.g., genomic instability detected by comet assays) using the spent media of exposed cell cultures, and to be prevented by DNMT1 and DNMT3A knock out cells [364]. Similarly, treatment with the DNMT inhibitor 5-azacytidine also reduces the impact of gamma irradiation [365]. Rugo et al. [364] suggest that DNAm by the DNMT is essential to maintain the secretory profile of insulted cells. The ability of exposed cells to affect distant cells is not unique to irradiated preparations. Arsenic-transformed cells can also mediate the transformation of normal stem cells into cancer stem cells through exosomes carrying multiple oncogenic factors (inflammation-related and apoptosis-related transcripts and proteins, and oncogenesis-associated microRNAs) [366]. Moreover, irradiation can induce senescence, during which cells develop a senescence-associated secretory phenotype (SASP), which involves the excretion of numerous factors that can elicit inflammatory and immune responses for the elimination of the senescent cells, and depending on the context, conditions promoting the emergence of tumorigenic cells [367]. Multiple forms of senescence (replicative, oncogene-induced, and stress-induced premature senescence) lead to the activation of *L1* retrotransposons that activates a type-I interferon response and the senescence-associated secretory phenotype [368]. Overall, the literature indicates that secretory products and inflammatory reactions, contribute to these temporal and distant effects on GGD methylation and genomic instability in unexposed cells or tissues [137,369,370].

Koturbash [268] reported that irradiation induces changes in DNAm that are cell type and assay dependent, with assays targeting L*L1* of old evolutionary age showing hypermethylation, while those of younger age showing hypomethylation. L*L1* hypomethylation can lead to its activation and promote aberrant transcription, alternative splicing, insertional mutagenesis, DNA damage, and genome instability [371]. Koturbash [268] reported that mechanisms leading to DNA hypomethylation following irradiation involve methionine depletion and impaired glutathione synthesis associated with irradiation-induced oxidative stress and DNA damage. The one-carbon metabolism pathway and DNA methylation of L*L1* (independent of evolutionary age) and pericentromeric major satellite DNA and expressions were also affected, although in a non-linear dose–response pattern, in the hearts of male C57BL/6 J mice after delays of 14 days, and interestingly at 90 days, following a single dose of radiation relevant to the space environment [372].

In addition to DNA hypomethylation, irradiation was shown to induce expression of DNMT3b that can contribute to the silencing of the tumor suppressor gene p53 and of p21 (cyclin-dependent kinase inhibitor 1A, *Cdkn1a*) and consequently favour tumorigenic phenotypes [269]. DNMT3B (as well as DNMT1 and DNMT3A) is frequently overexpressed in different cancer types [373], including nasopharyngeal carcinoma (NC) [269]. A series of experiments by Wu et al. [269] using NC cell lines demonstrated that irradiation induces dose-dependent (2, 4, 6, 8, 10 Gy) increases in DNMT3B mRNA and protein abundance but has no significant effect on DNMT1 and 3A. Silencing of DNMT3B inhibits EMT in NC cells (migration and invasion assays, E-CADHERIN increased, N-CADHERIN and VIMENTIN decreased), and induces cell cycle arrest and apoptosis through restoration of p53 and p21 function via DNA demethylation [269]. The mechanisms that link irradiation to increases in DNMT3B abundance remain to be defined.

### 4.5. H4K20 Methylation in Target vs. Non-Target Tissues, and Effects of Other Chemicals

Whilst differences in the DNAm system induced by PB were suggested to distinguish cancer-target from non-target tissues [201], methylation of H4K20 might also be an epigenetic marker that can differentiate a carcinogen target from non-target tissues. H4K20 mono-methylation is performed by KTM5A (alias SET8), followed mostly by di- and tri-methylation by KMT5B and C, respectively. Altered expression of these enzymes is involved in carcinogenicity mechanisms. Lung is a carcinogenic target following exposure to acrylamide (a Group 2A genotoxic carcinogen) in mice, but not the liver [374]. Testing dose–response effects of acrylamide in mice (0.0875 to 0.7 mM in drinking water for 28 days), de Conti et al. [270] observed similar levels of DNA adducts in lung and liver but contrasting global epigenetic alterations between tissues. Noticeably, the lungs displayed dose-responsive decreases in abundance of H4K20me3 and of its corresponding histone methyltransferase family (KMT5A/B/C); in contrast, liver showed increases in abundance of H3K27ac, larger number of sites with altered DNAm (hypo or hypermethylated), and a larger number of genes differentially expressed. The larger number of genes affected in liver as compared to lung was associated with the increases in abundance of H3K27ac and in DNA hypomethylated sites. De Conti et al. [270] observed that other carcinogens induce similar levels of adducts in target and non-target tissues (2-acetylaminofluorene, 1,3-butadiene), but with a decrease in H4K20me3 in the target tissues of 2-acetylaminofluorene, 1,3-butadiene, furan, and methapyrilene.

There is support that global loss of H4K20me3 and H4K16ac might be used as cancer biomarkers [108,179,375,376]. The abundance of H4K20me1 catalyzed by KMT5A is cell cycle dependent, with a peak during the G2 and M phases. In combination with other mechanisms [377,378], it promotes the chromatin compaction necessary for mitosis and the maintenance of genome integrity. Investigating the human bone osteosarcoma epithelial U2OS cell line exiting from mitosis, Shoaib et al. [376] demonstrated a decline in KMT5A and H4K20me1 promoting chromatin de-compaction, binding of origin recognition complex (ORC) to the chromatin in daughter cells (a complex that remains bound to chromatin at replication origins throughout the cell cycle and licensing for replication), opening of double-stranded DNA, and promotion of DNA damage and loss of genome integrity. Others have indicated that an increased expression of KMT5A induces chromosome segregation defects, indicating that progression through the cell cycle requires tight regulation of KMT5A and H4K20 methylation [379].

Moreover, H4K20me2 plays a major role in DNA repair processes and its binding partners (including 53BP1) appear to direct DNA double-strand break repair either through the homologous recombination (BRCA1-dependent) or the non-homologous DNA end-joining (53BP1-dependent) pathways [180,311]. On H4, reduced H4K16ac is associated with a wide range of cancers, with the exception of non-small-cell lung cancer in which its corresponding H4K16 acetyltransferase is highly expressed [375]. H4K16 is acetylated by the lysine acetyltransferase KAT8 using acetyl-CoA as substrate. Although H4K16ac de-compacts the chromatin, the mechanisms by which changes in KAT8 expression and H4H16ac abundance leading to tumorigenesis remain unclear [375]. Acetylation of H4K16 by KAT5 (alias TIP60) also appears to be involved in recruitment of binding partners for DNA double-strand break repair [180].

Overall, after studying acrylamide, 2-acetylaminofluorene, 1,3-butadiene, furan, and methapyrilene, and examining cancer-target and non-cancer-target tissues for adducts and epigenetic effects, only abundance of H4K20me3 and KMT5A/B/C could identify the cancer-target tissue [270]. Moreover, the comparison of arsenic-induced skin cancer to non-cancer tissues revealed depletion of H4K20me3 in subtelomeric DNA, resulting in arsenic-induced telomeric elongation and cancers [284].

Finally, among HPTM, generally, methylation at H3K4, H3K36, and H3K79 is associated with gene activation; methylation at H3K9, H3K27 and H4K20 is associated with gene silencing [379]; and histone lysine acetylation is associated with transcriptional activities [380]. Despite increasing numbers of HPTM [381,382] and of their emerging roles [383], currently, examples of HPTM that are frequently investigated in chemical hazard assessment include H3K4me3 (a memory of transcription activation [384]), H3K9me3 and H3K9ac (as opposing marker of heterochromatization), H3K27me3 and H3K27ac (opposing markers of poised and expressed genes), and H4K20me3 and H4K16ac as cancer biomarkers [34] and DNA damage response [311]. Thus, differences in abundance of H4K20me3 and KMT5A/B/C deserve further investigation as potential epigenetic markers of carcinogenic substances.

## 5. Overview of Potential Epigenetic Assay Types Adaptable to Chemical Hazard Assessment

Table 3 presents a list of assay types and genes that are known to be frequently epigenetically modified during carcinogenesis, more detailed information is provided in Part B. These gene/assay combinations are suggested for consideration as epigenetic markers of carcinogenic KE, and as partners with relevant assays to derive higher predictive values in carcinogen identification. Under the current series of KE (Figure 3), it is anticipated that a combination of a limited number of KE-specific assays could be sufficient to predict chemical carcinogenicity. Many of these assays were carefully evaluated by the current authors based on the methodology proposed by Jacobs et al. [10] and received good ratings. However, for many of these, further assay adaptation and validation experiments using human cells would be required to more faithfully match the criteria of Table 1, in order for the epigenetic data to be easily endorsed for the purposes of chemical hazard assessment.

Given the large number of potential epigenetic assays that can be considered, a provisional small workable subset of assays applicable to all cell types is suggested in Table 4, which can be prioritized for assay improvement and validation toward chemical hazard assessment. The following sections briefly describe Table 3 content, in addition to reasons for the selected subset of assays in Table 4.

### 5.1. Epigenetic Enzyme Screening Assays (Cell-Free Biochemistry, Enzymology Assay, High-Content Image Analyses, Reporter Systems, and QSAR Approaches)

Numerous companies offer cell-free biochemistry and enzymology assays that can generate concentration–response curves and that are used for the development of epigenetic drugs [385,386,387]. These assays can be adaptable to detect interactions between chemicals and epigenetic enzymes described in Table 3 in the context of chemical hazard assessment. Although results from cell-free screening assays are not definitive, they inform upon the capacity of a substance to directly interact with a target enzyme to support interpretation of mechanisms of action (see Section 8). This type of information is often necessary and complementary to observations from complex cellular assays from which the origin of the effect requires further investigation and confirmation of potential molecular initiating events [388]. It is within this context that these types of assays deserve further validation (Table 4).

High-content analysis (HCA) systems are based on image analyses of whole cells or components of cells [389], with simultaneous readout of several parameters including cell proliferation and phenotypic changes following treatments. Section 8.2 provides examples of HCA systems that have been used as high-throughput screening assays to investigate endogenous histone methyltransferases [390], and a series of histone demethylases [391,392], which are oncogenic drivers [179,393]. High-content imaging microscopy systems are particularly valuable in the first assay group (Table 3 and Table 4), given that the cell cultures do not need to be disturbed, the cells remain intact in the culture wells in contact with their neighbouring cells which could be of the same or of a different type. HCA systems measure multiple endpoints, which can then be expressed as the proportion of cells affected in a concentration–response manner rather than measuring a signal-average across a mixed cell population. This is important given that not all cells respond equally in toxicology, consequently, the percentage of cells with a significant response can be a more sensitive observation than the average response from a homogenate of the cell population. As alternative assays, flow cytometry analyses can also generate data regarding the proportion of cells affected with consideration of cell cycle and cell types, but the cells require manipulation [291,394]. On the whole, the high-content imaging microscopy systems for enzyme-specific assays such as HMT-HDM appear to be most informative, and since they are based on the simultaneous measurement of multiple endpoints and applicable to different cell types, they are versatile. High-content image analyses systems can be adapted to monitor changes in HPTM in combination with abundance of both “writer” and “eraser” enzymes and thus are included in Table 4. However, the selection of antibodies with appropriate specificity can be an issue (see Section 8.3), and, as with all in vitro assays, how appropriately they are combined will improve their capacity and utility in the prediction of human in vivo effects.

Reporter systems integrate the cellular complexity that is absent in biochemistry and enzymology assays and can integrate many factors responsible for enzyme activities (e.g., enzyme and cofactor abundance). DNAm reporter systems generate concentration–response curves of large magnitude facilitated by the measurement of a fluorescent/luminescent signal, and therefore offer the advantages of being amenable to a HTS format or to flow cytometry analyses [291,394]. Whilst some are already suggested as HTS for the development of epigenetic drugs, applications for chemical hazard assessment still need to be taken forward [395]. Five systems are described in Section 8.4; locus derepression systems include the YB5 system (a cytomegalovirus promoter-based assay [291,396]), and two others use endogenous promoters [397,398]. A locus repression system is presented using a combination of interacting constructs [399], and finally, the reporter for genomic methylation (RGM) system that use CRISPR/Cas9-guided insertion of the minimal promoter of the imprinted gene *Snrpn* (small nuclear ribonucleoprotein polypeptide) to detect decreases and increases in methylation [24,400].

QSAR methodologies have been used to assess reactivities of 1038 pesticides with the human DNMT1 and DNMT3A [401]. Among these chemicals, rodenticides (flocoumafen, brodifacoum, and difenacoum) were found as potential DNMT ligands, but these coumarin-related chemicals are highly toxic anticoagulants, not carcinogens [401]. Similarly, chemicals found to alter DNAm using the YB5 reporter system [291] were all bioactive but not necessarily carcinogens, and these include thiram (fungicide, bactericide, developmental and reproductive toxicant), pyrithion zinc (shampoo), cycloheximide (protein synthesis inhibitor), oxyquinoline (multipurpose, fungistatic, and alias 8-hydroxyquinoline), and cardiac glycosides; some belong to IARC group 3 (not classifiable as to their carcinogenicity in humans; thiram and pyrithion zinc). Overall, positive results in an epigenetic screening assay may highlight real concern in chemical hazard assessment, but not necessarily related to cancer. It is therefore important that the results from in silico methodologies, or from other screening assays, be considered in the context of the cancer IATA in association with other assays.

### 5.2. Changes in Global Genome Epigenetic Marks and in Repeated DNA Sequences

As indicated in previous sections, global genome DNA hypomethylation (GGDHo) is a characteristic of cancer cells [402]. Factors that normally contribute to DNA hypomethylation include (1) the nucleotide context, which can be monitored by next-generation sequencing methodologies (NGSm), (2) being a late replication locus, and (3) cells that have had a large number of cell divisions [403]. Progressive losses in DNAm occur predominantly in late-replicating heterochromatin DNA linked with the nuclear lamina (lamina-associated domain characterized by low gene and GC density) [403], supporting that specific CpG sites are better indicators of global genome DNA hypomethylation. Indeed, CpG sites that are more prone to hypomethylation represent only 13% of all CpG sites and are flanked by an A or T on both sides of such tetranucleotides (WCGW; W is the nucleotide symbol corresponding to A or T) [403], but these tetranucleotides are among the preferred methylation sites by DNMT1 [404]. In contrast, CpG sites robust to hypomethylation are associated with the presence of H3K36me3 histone marks that attract DNMT3B and that are located in gene bodies [403]. This information highlights the importance of the genomic context in contributing to hypomethylation susceptibility. Therefore, NGSm offer the advantages of providing genomic context information to derive an understanding of the origin of GGDHo, in addition to coverage for genes, retrotransposons, and DNA repeats. Consequently, NGSm are included in Table 4 as preferred approaches to further characterize the causes of global epigenomic changes, the timing throughout carcinogenesis for the development of these conditions, and the magnitude of changes that lead to carcinogenic events. However, NGSm are resource demanding, not readily available or applicable to large number of samples and require additional steps to measure 5mC and 5hmC. A discussion of alternative methodologies to measure GGDm in large numbers of samples but that do not consider sequence context is provided in Section 8.5. These include LC–MS, capillary electrophoresis (CE), cytosine extension assays, and flow cytometry.

**Figure 4:**
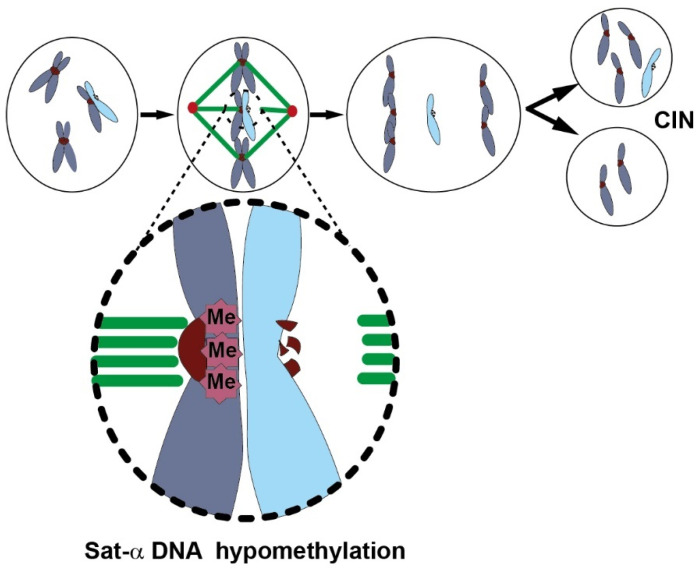
Satellite-α DNA hypomethylation is observed in cancer cells and may alter expression of SAT-α non-coding RNAs, essential for the insertion of centromeric histone variants (CENP-A, -B, and -C) into the DNA, for centromere and kinetochore assembly, and for the segregation of replicated chromosomes during mitosis and meiosis. Epigenetic destabilization of the centromeric/pericentromeric areas and of the kinetochore can lead to chromosome instability (CIN) and aneuploidy (Section 2.5.2).

Suggested assays in Table 4 generate data that are relevant to any tissue and that can be obtained from both in vitro and in vivo experiments, which can facilitate testing extrapolation and translation of in vitro observations to in vivo conditions and vice versa. Reduction in methylation of DNA repeats, such as retrotransposons (*L1*, *AluYb8*) and satellite DNA, might be valuable assays predictive of carcinogenicity because they are linked with genomic instability as adverse key carcinogenic events [405,406,407,408]. *Sat-α* DNAm can be measured precisely by pyrosequencing, and it offers a larger dynamic range than *L1* or the Sine *AluYb8* when comparing human primary liver cells to hepatic cell lines (HepG2 and HC-04) [176], or in clones from irradiated and non-irradiated primary human fibroblasts [370]. *Sat-α* is pericentromeric and epigenetic anomalies in this region induce centromere and kinetochore malfunctions, chromosomal instability, and cancers [174]. In contrast to retrotransposons, *Sat-α* DNA is located at specific genomic location in the human genome which may facilitate correlation with chromosomal instability (see Section 2.5.2, Figure 4). Therefore, *Sat-α* DNAm assay deserves consideration for further validation, as it might provide threshold levels for induction of genomic instability that could be pivotal for KERs in an NGTxC IATA.

**Figure 5:**
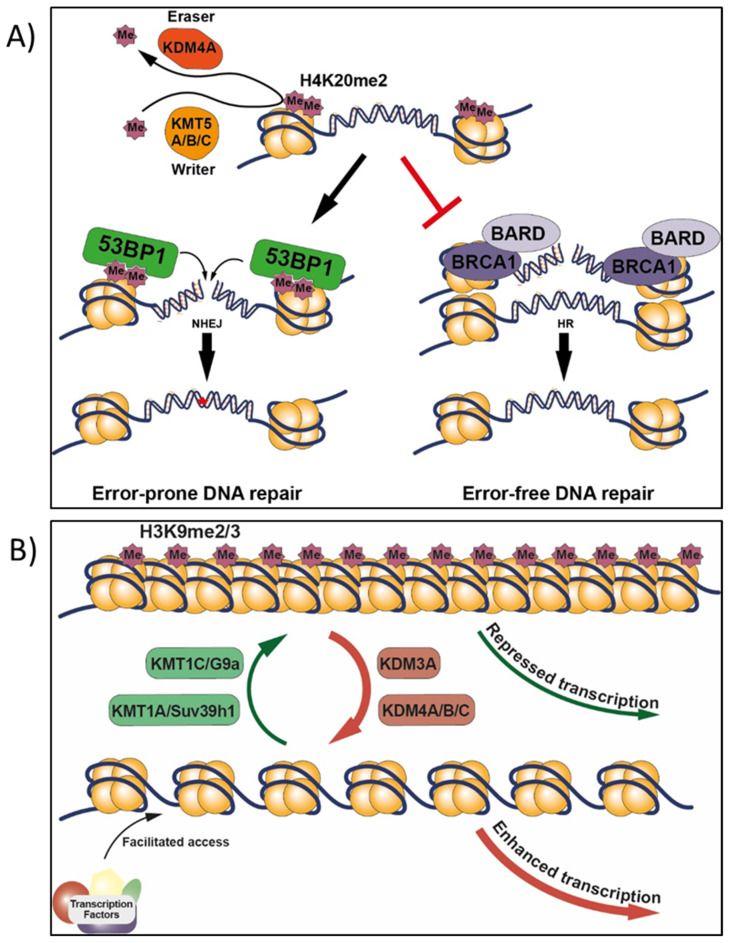
(**A**) H4K20me2 plays a major role in DNA repair processes. It binds 53BP1 and promotes DNA double-strand break repair through the error-prone 53BP1-dependent non-homologous DNA end-joining (NHEJ) pathway, thus preventing activation of the error-free BRCA1-dependent homologous recombination (HR) DNA repair pathway. Decreases in H4K20me3 can be induced by chemical exposure and to differentiate carcinogen target from non-target tissues. (**B**) H3K9 dimethylated by KMT1C silences DNA repair enzymes, and with H3K9 trimethylated by KMT1A, are markers of heterochromatin with repressed genes. Decreases in H3K9me2/3 abundance create epigenetically permissive conditions that facilitate access to transcription factors for the activation of genes normally silenced, including oncogenic genes. Assays measuring the abundance of these histone marks and the activities of their respective lysine methyltransferases (KMT) and lysine demethylases (KDM) can be useful in chemical hazard assessment.

Histone tail modifications are other epigenetic marks that can be measured as indicators of chemical-induced global genome changes. Following chemical exposure, decreases in H3K9me3 and H4K20me3 are recommended as provisional histone modifications to be further validated as indicators of unstable heterochromatin in cells that may escape senescence to become carcinogenic (Table 4; Figure 5). Decreases in H3K9me3 and H4K20me3 abundance are associated with escape from senescence [409,410], loss of heterochromatization, displacement of the chromatin from the nuclear lamina, genomic instability, and carcinogenesis [411]. Following chemical exposure, decreases in H4K20me3 were also reported to distinguish carcinogen target from non-target tissues [270]. Others compared normal tissues and cancers by mass spectrometry analyses and revealed changes in H3K27me2/me3 (increases and decreases depending on tissues), decreases in H4K20me3, in H4K16ac, and in H3K14ac, as frequent modifications associated with cancers [34]. Validating the measurements of H3K9me3 and H4K20me3 are provisional suggestions given the complexity of epigenetic mechanisms and rapidly growing epigenetic knowledge.

**Figure 6:**
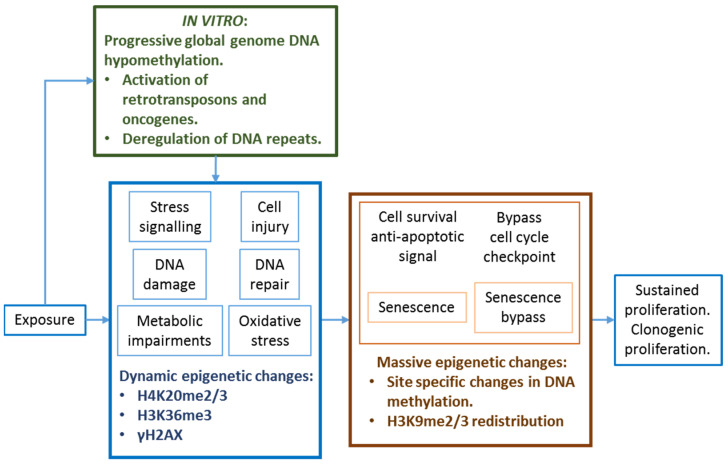
Illustration of representative sequential epigenetic changes that are known to occur in vitro in primary cell cultures in the absence of chemical treatment. These events can be promoted by chemical carcinogens.

In fact, for chemical hazard assessment time-series experiments documenting changes in HPTM are required to identify the combination of HPTM that are predictive of carcinogenesis, which may be possible only in long-term experiments (as illustrated in Figure 6). Early changes in DNAm and HPTM following chemical exposures can be indicative of a series of normal processes and not epigenetic anomalies. For example, an increase in γH2AX abundance suggests induction of DNA damage as it provides sites for recruiting DNA repair enzymes [412,413]. DNA double-strand breaks are recognised by 53BP1 through its interaction with H4K20me2 and H2AK15ub to contribute to non-homologous end-joining DNA repair, but this interaction is prevented by H4K16ac to favour homologous recombination DNA repair mechanisms [311]. The DNA repair may be successful, or not, and the cell may enter senescence, leading to apoptosis, as opposed to carcinogenesis. Cells in senescence show increases in H4K20me3 (mediated by KMT5B and 5C) and the redistribution of H3K9me3 silencing RB/E2F target genes, inducing genome stabilization and tumour suppression [414,415]. Cells in senescence demonstrate cell cycle arrest, survival mechanisms, the release of cytokines and soluble factors (senescence associated secretory phenotypes), and the loss of lamin B1 protein (LMNB1) from the inner nuclear membrane; and all together these events involve DNAm changes, redistribution of HPTM, and profound reorganization of the euchromatin and heterochromatin [411,416]. Then, cells in apoptosis induce a global decrease in histone acetylation [417,418,419], increases in H4K20me3 [418] andH3K27me3, and smaller increases in H3K4me3 and H3K9me2 [392]. Cells that may acquire carcinogenic potential escape apoptosis and senescence through activation of the WNT and NOTCH pathways associated with increased abundance of H3K9me3 [409], and altered p53, retinoblastoma (RB), or telomerase pathways [420]. Interestingly, loss of H4K20me3 and of heterochromatization in subtelomeric regions was associated with telomere elongation in arsenic-induced skin cancers [284]. This HPTM is an addition to the epigenetic mechanisms regulating telomere elongation as a major contributor to human carcinogenesis [421]. Overall, global genome changes in HPTM are more complex to interpret than global genome DNAm. Time-series and long-term experiments are likely to have greater potential at identifying appropriate HPTM as epigenetic markers of steps leading to carcinogenesis.

Figure 6 shows sequential events leading to the clonogenic proliferation of potentially carcinogenic cells. Even in the absence of chemical treatment, primary cells in culture display a gradual decrease in global genome DNA methylation that may lead to deregulation of retrotransposons, oncogenes and DNA repeats. The magnitude of DNA methylation changes required to induce these events needs further characterization. In the emerging clones, additional epigenetic markers that can contribute to immune evasion, reduction in inflammatory signals, and angiogenesis, might be detectable (see Section 9), but without the in vivo influences of other cellular components. In the use of cell lines, special care needs to be taken in the selection and in the interpretation of results, as it is likely that the cells are already extensively transformed to bypass these protective anti-cancer mechanisms.

### 5.3. Consideration of Multiple DMR/Genes in Parallel as Markers of Key Events

Table 3 presents a series of genes for which the expression was shown to be epigenetically regulated and that can target specific carcinogenic key events (detoxification and DNA repair pathway, cell cycle regulation, inflammation/immune response, cell morphology and cytoskeleton, angiogenesis, telomerase, imprints, and reprogramming transcription factors; see Section 9). Relevant chemical hazard assessment assays can be developed based on these genes, using pyrosequencing, targeted NGS, multiplex methylator phenotype, or epigenetic signature approaches. Efforts have been initiated to identify the magnitude of epigenetic changes required to regulate gene expression [422]; however, further knowledge on this topic would be needed before including such data as part of the weight of evidence for regulatory testing strategies.

Some genes listed in Table 3 are among a series of 13 (out of 1800 epigenetic biomarkers) found to be clinically relevant and with subsequent commercial assay development [210]. These include glutathione *S*-transferase P (*Gstp1*), adenomatous polyposis coli protein (*Apc*), ras association domain-containing protein 1 (*Rassf1*), N-myc downregulated gene 4 (*Ndrg4*), bone morphogenic protein 3 (*Bmp3*), septin-9 (*Sept9*), short stature homeobox protein 2 (*Shox2*), twist-related protein 1 (*Twist1*), homeobox protein OTX1 (*Otx1*), one cut domain family member 2 (*Onecut2*), methylated guanine methyltransferase (*Mgmt*), branched-chain-amino-acid aminotransferase (*Bcat1*), and DNA-binding protein Ikaros (*Ikzf1*). Among these, assays for *Gstp1*, *Ndrg4*, *Sept9*, and *Mgmt*, are included in clinical guidelines, and two *Ndrg4* and *Sept9,* have been approved by US-FDA [210]. The DNAm level reached in these genes is linked to long-term changes associated with disease progression; whether or not such epigenetic markers can also be useful in the detection of early chemically induced changes predictive of carcinogenesis remains to be demonstrated.

The concept of “oncogenic module” indicates that although each oncogene contributes to tumorigenesis, they operate in a cooperative manner for ensuring robustness of the tumorigenic process [423]. Therefore, multiplex DNAm assays can be developed to monitor multiple genes considering the various signaling pathways and key events leading to cancers. The “methylator phenotype” approach consists of relying on the DNAm of a limited number of genes as informative biomarkers in clinical epigenetics for diagnostic and therapeutic purposes [192,193]. For example, a DNAm signature based on the combination of four genes (*Bcat1*, *Cdo1*, *Trim58*, and *Znf177*) in bronchial fluids achieved higher diagnostic efficacy compared to conventional cytology to detect minimally and non-invasive lung cancers [424]. Such DNAm signatures that distinguish normal from lung tumour samples can be obtained from sputum, bronchoalveolar lavage, and blood [425]. Assays for more complex DNAm signatures, such as the “epigenetic for cancers of unknown primary” (EPICUP), identify the primary site of cancer originthus assisting diagnosis and cancer treatment [426]. Taken together, these gene set assays can be adapted for chemical hazard assessment.

A transcriptomic biomarker (or signature) is a set of transcripts induced by a specific event that can be useful in chemical hazard assessment, as well as clinical diagnostic, prognostic, and therapeutic purposes [427,428]. Transcriptomic analyses are being improved to identify signatures associated with specific chemical classes and modes of action and may eventually assist in the identification of NGTxC using in vitro [429], and short-term in vivo rodent data [430]. Transcriptomic approaches [431] are data rich, and they offer the possibility of using the same sample to assess multiple signatures in parallel in a screening strategy. Short-term transcriptomic analyses of TK6 human lymphoblastoid cells exposed for 4 h to chemicals permitted the identification of histone deacetylase inhibitors (HDACi) based on a signature of 81 genes. This assay is referred to as the toxicogenomic (TGx) TGx-HDACi biomarker [432]. This data-rich approach may enable the identification of other gene sets as part of the same analysis that can be specific to additional epigenetic mechanisms (histone methyltransferase, demethylase, etc.), which can lead to the development of more complete epigenetic screening assays. The same strategy was previously used to develop the TGx-DDI [428], an assay that identify a signature that corresponds to GTxC on the basis of gene induction for DNA repair and stress signaling pathways [428,433,434,435]. The TGx-DDI and TGx-HDACi assays can be successfully conducted concurrently in a screening strategy using the same samples [432]. Induction of progression then needs to be established across carcinogenic KEs.

The reliable identification of GTxC is easier to achieve than identifying NGTxC given the multiple modes of action of NGTxC, and the requirement of chronic exposure for the NGTxC induction of tissue-specific cancers. Schaap et al. [429] used a signature of 60 differentially expressed genes in primary mouse hepatocytes and embryonic stem cells exposed to chemicals for 8 or 24 h, respectively, and could correctly categorise most of the NGTxC by similarity of signatures with known NGTxC, with greater accuracy after combining both cell type analyses. In short-term experiments, chemical-induced gene expression can rapidly change and can reduce consistencies in signature assays. Efforts for the identification of signatures of NGTxC based on rodent short-term in vivo experiments using genes with less dynamic expression changes are also progressing. Using machine learning and publicly available hepatic datasets Huang et al. [430] exposed male Sprague Dawley rats to chemicals for 1 to 28 days and identified a signature of 9 genes (upregulation of *Akr7a3, Aqp7, Cdc2a*, and *Cdkn3*, and downregulation of *A2m, Ca3, Cyp2c11, Ntf3*, and *Sds*) for which expression levels remain unaltered through time and that can be measured in 3-day exposure experiments to predict NGTxC and other hepatic diseases. By combining gene expression and DNAm analyses, Ito et al. [436] identified four genes (*Ldlrad4*, *Proc*, *Cdh17*, and *Nfia*) silenced and hypermethylated after 28 days of exposure to non-genotoxic hepatocarcinogens (carbon tetrachloride, thioacetamide, methapyrilene hydrochloride) in rats, that remained silenced until the end of the 90-day experiment. The authors concluded that these genes may be in vivo epigenetic markers of non-genotoxic hepatocarcinogens. Note that this set of genes was not found to be a marker for a renal NGTxC (ochratoxin A, vs. renal genotoxic 1,2,3-trichloropropane, 1-amino-2, 4-dibromoantraquinone, and nitrofurantoin) [436], exemplifying the complexity and tissue specificity for effects of NGTxC. Clearly, designing testing strategies based on a predetermined set of genes requires additional work to consider chemicals, tissues, and species specificity.

## 6. Strategy for Evaluators to Assess the Importance of Cancer Epigenetic Data in Chemical Hazard Assessment

**Figure 7:**
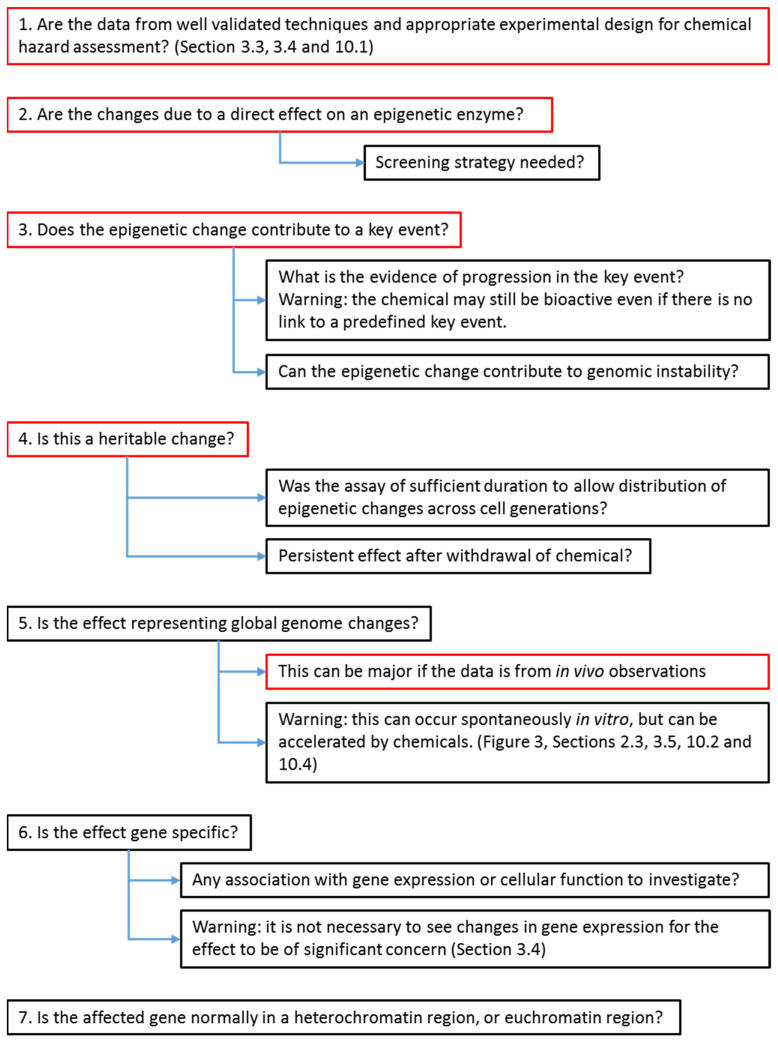
Framework to assess the importance of cancer epigenetic data in a weight of evidence approach in chemical hazard assessment for NGTxC.

A structured way forward to look at how epigenetic mechanisms can be appropriately interrogated and considered in a weight of evidence NGTxC IATA approach is proposed in Figure 7. A series of provisional questions are suggested to evaluate cancer epigenetic data. The boxes are numbered for identification purposes and those in red highlight questions that are of likely greater concern. The detection of a direct effect of a chemical substance on an epigenetic enzyme, for instance, may be vital to cellular functions (question 2). Demonstrating that the epigenetic anomalies contribute to a carcinogenic key event will reduce NGTxC IATA uncertainties (question 3). In relation to question 4, the importance of running the appropriate assays over a sufficiently long duration, as discussed herein, cannot be over emphasised for the detection of heritable changes. Genes from heterochromatin regions that become active during carcinogenesis under the influence of pioneering transcription factors require longer assessment delays before becoming detectable [112]. The investigation of DNA repeated sequences, the application of HTP-NGS epigenetic analyses demonstrating abnormal heritable epigenetic reprograming occurring in the absence of sustained exposure, and additional key events will help pinpoint the identification of NGTxC.

## 7. Conclusions

The absence of validated testing strategies for the identification of NGTxC remains an important regulatory issue. The IATA for NGTxC [10] allows for the positioning of empiric knowledge derived from assays, such as epigenetic assays, relative to carcinogenic KEs. This approach demonstrates biologically plausible roles for assay measurements in carcinogenesis and for the identification of NGTxC. However, despite frequent epigenetic anomalies induced by exposure to chemicals and reviewed by numerous teams [7,35,36,37,38,39,40,42,47], epigenetic data are frequently deficient in many aspects for appropriate consideration in chemical risk assessment.

Here, we have reviewed principles of DNAm and histone modification and potential epigenetic endpoints and assays that could be optimised considering chemical hazard assessment criteria for use in the NGTxC IATA [10]. We examined chemical case studies of epigenetic effects (arsenic, nickel, and phenobarbital) to exemplify different epigenetic mechanisms of action. Some epigenetic changes appear to be more common outcomes across chemical effects (GGDHo, redistribution of the binding of the chromatin organizer CTCF protein, and HPTM alterations), whilst others may be more specific (i.e., H4K20me3 abundance). Inhibitory effects of Ni on histone methyltransferases, demethylases, and various dioxygenases emphasise that complete cellular systems are preferred tools (as compared to in vitro enzymatic assays targeting one enzyme) to adequately understand and apply epigenetic anomalies. Both arsenic and Ni studies support the need for complete cellular systems to accommodate all key compensatory/counter balancing mechanisms, supported by epidemiological studies where available.

The more recent techniques were more reliable and provide convincing evidence that epigenetic mechanisms are involved in carcinogenesis, and that they can be measured. Next-generation sequencing-based methodologies (NGSm) such as whole-genome and reduced-representation bisulphite sequencing (WGBS and RRBS) assays, provide reliable indicators of global and site-specific changes in DNAm demonstrating chemical-induced epigenetic anomalies. Such methodologies are required for the development of chemical-induced carcinogenic methylator phenotypes or epigenetic signatures for the identification of NGTxC. DNA methylation is an epigenetic mark that is less complex than HPTM. DNA methylation research is being applied in the clinical field, and this knowledge and related tools can be adapted for chemical hazard prediction purposes. Using NGSm to develop multiplex DNAm assays covering multiple KE of the IATA would provide better coverage of altered pathways conducive to carcinogenic progression. Collectively, assays demonstrating effects on epigenetic driver genes, effects in epigenetic enzymology assays, and assessment of underlying oxidative stress and metabolic disturbance (reducing co-factors availability) as causes for epigenetic outcomes are important for an understanding of MIE and mechanisms of action leading to carcinogenicity. Both short- and long-term in vivo and in vitro approaches are necessary in the development of epigenetic testing strategies.

Global genomic changes in HPTM can be indicative of DNA repair, of short-term gene expression changes in adaptive responses, senescence, and cellular transformation processes. Site-specific HPTM analyses through chromatin immunoprecipitation and NGSm can further explain gene expression changes. However, the complexity of HPTM systems makes global genome changes in HPTM difficult to interpret without time-series experiments. Nevertheless, screening assays demonstrating a direct effect on the activity of an epigenetic enzyme (writer, reader, and eraser) may not necessarily relate to cancer, but are adverse, and worthy of further investigation.

It is apparent that although targeting epigenetic endpoints informs carcinogenicity assessment, no single assay can target the broad range of mechanisms that contribute to human carcinogenesis in variable tissues. It is only in combination with other assays that carcinogenicity can be more confidently predicted. Overall, we conclude that there is sufficient methodological and mechanistic knowledge to initiate the incorporation of epigenetic tools described herein into the OECD IATA for NGTxC in a specified and selective manner, both for the general NGTxC IATA model and toward the future design of cancer-specific IATA formats. We suggest a provisional short list of endpoints (Table 4: *Sat-α*, H4K20me2/3, and H3K9me2/3) and NGSm assays requiring further development/validation considering chemical hazard assessment criteria (Table 1). As indicated previously [10], it is anticipated that an appropriate combination of a few assays (epigenetics and others) into the IATA will assist in reducing the number of tests required to establish a prediction of chemical carcinogenicity, as well as reducing uncertainties associated with such a prediction. We also suggest a structured framework for the interrogation and evaluation of cancer epigenetic data within a weight of evidence (WoE) approach (Figure 7).

Mechanisms that involve miRNAs have not been substantively included in this review; however, it is well noted that an increased understanding of these types of epigenetic interactions and of the robustness of miRNA assays is also needed, and that better incorporation of such observations from short-term in vitro and in vivo studies into IATA for NGTxC is also necessary in chemical hazard predictions (see for example [7,40,223]).

Epigenetic anomalies can induce genomic instability and reprogramme the expression of genes involved with cancer hallmarks. Inclusion of epigenetic anomalies as “modes of carcinogenic action” [15,177] needs to be with equivalent relevance as other modes, including “mutagenicity, mitogenesis, inhibition of cell death, cytotoxicity with reparative cell proliferation, and immune suppression” [10,437].

To identify epigenetic phenotypes that can predict NGTxC, in line with criteria described in Table 1, it is important important that international efforts be invested in designing and validating epigenetic screening and epigenetic investigative assays, using a selection of human primary cells, carefully selected cell lines, and rodent models. In pursuing these goals, the OECD expert group will further develop critical aspects arising from the series of assay reviews, such as (1) cell or tissue selection, (2) specific endpoints to monitor, (3) threshold characterization, (4) chemicals to be used as controls for positive or negative toxic vs. non-toxic effects, and GTxC vs. NGTxC [438]. Overall, integrating epigenetic assays into the NGTxC IATA will provide more efficient detection of all carcinogens and reduce uncertainties in chemical carcinogen predictions for better public health protection.

Having confirmed the scientific credibility and current state of the art for under-standing epigentic mechanisms in NGTxC pathways, the following sections expands upon the assay summaries in Table 3 (Section 5), with four further sections addressing assay development, markers, and application status. Section 8 discusses relevant assay types (biochemistry, image analyses, reporter genes, and global genome analyses). Combined in tailored ways, they are the building epi-mechanistic information blocks that will underpin the NGTxC IATA. Section 9 addresses relevant epigenetic endpoints on the basis of the key event/hallmark/characteristics of carcinogenesis identified in structuring the NGTxC IATA [10]—specifically, inflammation, immune evasion and immune suppression, cell morphology and cytoskeletal changes, senescence and telomerase, and angiogenesis. In Section 10 and Section 11, transcriptomic biomarkers, DNA methylation (and 5hmC) assay performance, and extrapolation of data from experimental models to human are further discussed.

## 8. Types of Relevant Epigenetic Assays

### 8.1. Cell-Free Biochemistry/Enzymology Assays

Pharma have developed a large number of biochemical assays to study epigenetic enzyme kinetics to screen drugs for their potential to regulate these enzymes, to identify adverse drug–drug interactions, and for safety assessment in drug discovery. The assays are based on either radioactivity, fluorescence, chemiluminescence, antibody, or MS technologies, and have been reviewed in various publications [385,386]. Gul [387] reviewed the performance of mostly commercially available assays for testing candidate epigenetic drugs, including seven histone acetyltransferases (HAT), seven histone deacetylases (HDAC), six histone methyltransferases (HMT), nine histone demethylases (HDM), and four bromodomain assays. MS-based technologies offer the advantages of detecting multiple modifications (unmethylated, mono-, di-, and tri-methylated) within the same substrate [439]. The choice of biochemical epigenetic assays is expanding [440]. Issues for consideration in selecting assays include interference from fluorescent compounds, antibody qualities, radioactive waste, equipment needed, simplicity and adaptability to high-throughput screening (HTS). To avoid false readings, Gul [387] suggested confirming observations using more than one assay.

### 8.2. High-Content Image Analysis (HCA) In Situ Assays

HCA enables the real-time tracking of locus-specific epigenetic marks within living cells and permits the investigation of epigenetic changes during cell differentiation and chemical treatments. Luense et al. [390] investigated activities of the lysine (K) methyltransferase EZH2/KMT6A, which is a component of the polycomb-repressive complex 2 (PRC2 includes EZH2, EED, SUZ12, and RBBP4) that adds a methyl group to H3K27me1 to create the histone marks H3K27me2/3, and these contribute to the silencing of tumour suppressor genes (TSG). The assay is conducted over 3–5 days using either the MDA-MB-231 or the MCF7 breast adenocarcinoma cell lines, or the HeLa S3 cervix adenocarcinoma cell line. Multiplex staining of the cells enabled the direct correlation of the level of EZH2 and H3K27me3 for every single nucleus. In the presence of EZH2 inhibitor, they demonstrated a genome-wide switch from H3K27me3 to H3K27ac in all three cell lines. Inhibition of EZH2 activities by indazole inhibitors (EPI-0023 and -0009) can be associated with a direct effect on the enzyme, whereas effects of the nucleoside analogue 3-deazaneplanocin (DZNep) can occur through changes in *S*-adenosyl-methionine (SAM) and *S*-adenosyl-homocysteine (SAH) metabolism, and inhibition of the SAH hydrolase. Effects of EZH2 inhibitors on the abundance of H3K27me3 were independent of the cell cycle. They demonstrated the performance of their assay by testing nine potential EZH2 inhibitors including the nucleoside analogue GSK343, and other indazole/pyridone derivatives. Notably, although the technique is adaptable to HTS, they reported a decrease in robustness of the assay following automation and miniaturization from a 384-well to a 1536-well plate assay [390].

In contrast to histone methyltransferase, Mulji et al. [391] developed a JMJD3/KDM6B histone demethylase assay based on constructs of active and inactive demethylase transfected into human embryonic kidney cells (HEK293 MSRII). They confirmed the specificity of this demethylase acting exclusively on H3K27me2/3, without affecting methylation of K27me1, or of K4 or K9 methylation. The assay was conducted using subset of the GlaxoSmithKline (GSK) screening collection of 87,500 cell-permeable compounds tested at 10 µM, and 3307 showed ≥20% inhibitions which were then tested in full concentration–response assays (up to 100 µM). A total of 784 of these showed potencies between 25 µM and 400 nM, and some were prioritised for further testing with MS. The latter analyses showed both direct and indirect effects on the KDM6B catalytic site.

In addition to KDM6B [391], seven other KDM were studied through high-content analysis (HCA) systems [392]. To better understand how chemicals can interfere directly or indirectly with these enzymes, the HCA assays monitored cell proliferation, the number of healthy, apoptotic, and necrotic cells, as well as specific demethylases and their histone marks (KDM3A for H3K9me2; KDM4A/B/C for H3K9Me3; KDM6B for H3K27me3; KDM5A/B/C for H3K4me3). Monitoring cellular status was important to avoid confounding effects; for example, some chemicals inducing apoptosis were found to increase the abundance of H3K4me3 (i.e., paclitaxel), but others did not (i.e., doxorubicin). The Jumonji C (JMJC) family of KDMs, comprising the KDM2 to KDM7 subfamilies, belong to the superfamily of Fe(II)- and 2-oxoglutarate (2-OG, alias α-ketoglutarate)-dependent oxygenases. All eight KDM tested are 2-OG-dependent oxygenases, and almost all currently available JMJC-KDM inhibitors act via metal chelation and compete with the 2-OG co-substrate, for binding at the active site [392]. Assays that use lower than physiologically relevant 2-OG concentrations (1000 µM) may overestimate KDM inhibition [392]. In addition to Fe(II), the JMJD2/KDM4 subfamily contains a zinc binding site close to the active site, and it has been demonstrated that its activity can be inhibited by zinc ejecting compounds such as ebselen [441,442]. Monitoring chemical effects on these oncogenic driver enzymes inform on chromatin functions given that H3K4me2/3 and H3K36me3 are associated with transcriptionally active genes whereas H3K9me2/3, H3K27me2/3 and H4K20me3 are associated with transcriptional repression.

Overall, HCA systems with or without automation and miniaturization can be highly informative for chemical HTS assays. Reporter gene systems can be affected by spatial context and locus near the integration sites. However, HCA in combination with emerging technologies independent from reporter systems (such as the BiAD-BiFC engineered protein complex [443]) enables the real-time tracking of locus-specific epigenetic marks within the nucleus of living cells. The BiAD-BiFC system includes a bimolecular anchor adaptor (BiAD) targeting the specific DNA locus (which could also be based on CRISPR/dCas9 anchoring), and a bimolecular fluorescence complementation (BiFC) protein targeting the epigenetic mark of interest, when adjacent to each other the BiAD-BiFC complex emits a fluorescent signal [443]. Such technologies are promising for the investigation of chemically induced epigenetic disruption.

### 8.3. Antibody Requirement for Enzyme-Specific Assays

Antibodies are required for HCA systems, Western blot, enzyme-linked immunosorbent assay (ELISA), and other enzyme-specific assays, however, antibodies are frequently of animal origin and with sometimes inaccurate target specificity. Tests that are dependent on antibodies from animal origin are not encouraged by the OECD Test Guidelines Programme (TGP) without proper justification. Although some believe that antibodies of animal origin are still needed [444], the EU Reference Laboratory for alternatives to animal testing—European Centre for the Validation of Alternative Methods (EURL-ECVAM) recommends that “animals should no longer be used for the development and production of antibodies for research, regulatory, diagnostic and therapeutic applications”, and indicates “In the EU, the provisions of Directive 2010/63/EU should be respected and EU countries should no longer authorise the development and production of antibodies through animal immunisation, where robust, legitimate scientific justification is lacking” [445]. Antibody specificity is a major source of difference across studies in assessment of epigenetic endpoints, this has encouraged the adoption of common validation procedures. Pre-validation of antibody performance can be conducted based on publicly available protocols (e.g., https://thisisepigenetics.ca/for-scientists/protocols-and-standards, accessed on 6 October 2021). In the case of green fluorescence protein-tagged histone (H3) and terbium-labeled anti-H3, the position of HPTM to investigate may affect the system sensitivity and accessibility of the antibody to the target site. The animal origin and performance of antibodies require careful consideration in relevant assay selection.

### 8.4. Reporter Systems in the Cellular Environment

Reporter assays can be designed for various needs and may be specific to promoter or integration sites. Numerous reporter assays are being used to study gene regulations and their impact on phenotypic changes [398,446]. Cancer or non-cancer cell lines may be useful as a “carrier” to investigate direct or indirect interactions of a chemical with a reporter system [447], but “normal” cells that have not already been transformed are preferred with respect to initiation of non-genotoxic carcinogenic processes. Historically, the responses of reporter assays can be biased by technical issues (selection of vector, transfecting reagents, antibiotic response, variable transgene expression [448]), by crosstalk with endogenous cell receptors, epigenetic silencing of the construct [449], and cellular heterogeneity [450], so their use requires adequate cell characterization and key mechanisms identification, in order to minimise the problems. The interpretation of the data from epigenetic reporter systems also need to be accompanied by considerations of the spatial context and locus (euchromatin, heterochromatin) proximal to the integration sites that can influence the spreading of epigenetic marks. Clone and subclone selection can influence the applicability of the reporter systems. For example, logically selecting clones with strong responses after 5aCdR treatment (a DNMT inhibitor) to favour the development of DNA hypomethylation assays, may prevent identification of clones that could mimic other gene types requiring both DNA hypomethylation and histone modifications to become responsive.

The YB5 system has been used for the detection of DNA methyltransferase inhibitors (DNMTi) and/or histone deacetylase inhibitors (HDACi), it generates a green fluorescent protein (GFP) signal in response to demethylase and acetylase activities [396,451]. It also permitted the discovery of Ca^2+^ signaling as an epigenetic mechanism [291]. The YB5 system includes a single copy of a construct composed of a hypermethylated cytomegalovirus promoter driving expression of the GFP in the human colon cancer cell line SW48 [396]. The transgene is integrated into an intragenic region of human EST CD655906 on chromosome 1p31.1, the locus is transcriptionally silent, and has been shown to be stable over time. GFP expression is silenced in >99.9% of the cells [291], but all cells are not equally responsive to chemical treatments. Therefore, DNA hypomethylation is permissive but not always sufficient on its own to induce the re-expression of silenced genes. DNA hypomethylation, chromatin structure resetting including H3K9 acetylation, decreased abundance of H3K27 trimethylation, and nucleosome eviction are associated with the re-expression of the silenced gene [396]. The epigenetic stability of the transfected reporter system for testing demethylase activity is not an issue given that it is already hypermethylated. Interestingly, re-silencing of the reporter system was observed within 5 days after withdrawal of the 5aCdR treatment, attributed mostly to the reassembly of the nucleosomes than DNA re-methylation [396]. The YB5 system was used to test 1206 drugs and demonstrated that many FDA-approved drugs can either enhance or antagonise known DNMTi (5aCdR) and HDACi (trichostatin A) activities [451].

In contrast to the YB5 system that uses a hypermethylated viral promoter construct, Cui et al. [398] developed a reporter system inserting a S*Sfrp1-Gfp* reporter construct into exon-2 of the *Sfrp1* locus. This recombinant allele behaves like the unaltered allele and both remain silenced by DNA hypermethylation in the colon cancer cell line HCT116, unless activated by treatment with a DNMT inhibitor (5aCdR), or with the histone deacetylase inhibitor trichostatin A (TSA). The effect of TSA when administered alone, was transient and detectable only when cells were seeded at low density. It is interesting to note that an effect of seeding density on DNA hypomethylation of the DNA repeat *AluYb8* in HepG2 cells was gene and cell line specific [176]. The system developed by Cui et al. [398] also suggests that DNA hypomethylation is permissive but not always sufficient to induce silenced genes re-expression. Indeed, the comparison of GFP-positive and GFP-negative cells following 5aCdR treatment demonstrated that DNA hypomethylation occurred in both group of cells despite differences in GFP expression. This test method does not appear to have been applied to chemical hazard testing, as yet.

Okochi-Takada et al. [397] established a system for the detection of DNA demethylating agents suitable for HTS. The construct includes luciferase as well as the enhanced green fluorescent protein (*eGfp*) gene driven by an endogenous promoter, the Ubiquitin carboxy-terminal hydrolase L1 (*Uchl1*) promoter. The *Uchl1* promoter is associated with a CpG island usually silenced by methylation in colon cancers. The methylated construct was transfected into the HCT116 colon cancer cell line, and a subclone that expressed luciferase and eGFP after 5aCdR treatment was selected to test the 19,840 small molecules of the RIKEN Natural Products Depository (NPDepo) chemical library. The luciferase is secreted in the medium and is measured with the Ready-To-Glow^TM^ assay, while the eGFP signals remain within the cells and is read by microscopy. The detection sensitivity was 10-fold higher using the luminescence than fluorescence signal. Only 5aCdR and 5-azacytidine (5aCR) generated a response, which led the authors to conclude that this assay will be useful for screening drug candidates and chemical carcinogens inducing DNA hypomethylation [397].

The previous assays were “locus de-repression” assays in which epigenetically silenced reporter systems are activated by epigenetic acting substances (demethylation and acetylation activities). The Hsiao laboratory published a series of studies using a “locus repression” assay that relies on a two-component reporter gene system that can be transfected into different cell types and can monitor silencing of a chosen promoter [399,452,453]. The first component is a construct that includes a promoter sequence of interest (selected depending upon application need) that regulates the expression of a tetracycline (TET) repressor. The second component is a construct that includes a *Cmv* promoter separated from the *eGfp* by a TET repressor binding site. When the first component is not epigenetically silenced, the tet repressor is expressed and binds the tet repressor binding site on the second construct to prevent eGFP expression. In contrast, when the promoter of the first component is epigenetically silenced, then the tet repressor is not expressed and the second component is active and expresses the eGFP. Therefore, this repression assay is based on the epigenetic status of the promoter in the first component and expression of the eGFP signal from the second construct. The functionality of this system was demonstrated using targeted DNA methylation (TDM), a method by which the transfection of a methylated genomic fragment with the same sequence as the target locus (here the promoter of the first component) is used as hemi-methylated docking site for DNMT1 during DNA replication and that progressively leads to the silencing of the promoter through cell division. Using TDM and the two-component system transfected in human bone marrow-derived mesenchymal stem cells (MSC), the following were shown: (1) methylation silencing of *Trip10* (thyroid hormone receptor interactor 10) promoter during the differentiation of the MSC to neurons and to osteocytes [399], (2) concurrent methylation silencing of *Hic1* (hypermethylated in cancer 1) and *Rassf1a* (ras-associated family protein isoforms 1A) is sufficient to induce malignant transformation of MSCs [453], and (3) screening a library of 169 compounds, mostly procainamide derivatives (active reactive oxide metabolite N-acetyl procainamide (NAPA)), for DNA demethylase activities using the *Trip10* system in the MCF7 human breast cancer cell line [452]. With the latter demonstration of suitability to a (limited) chemical applicability domain, this is a promising platform to screen chemicals for DNA methylation capability.

While the previous systems were developed by random insertion of reporter constructs, the reporter for genomic methylation (RGM) system is a CRISPR/Cas-guided insertion model [24,400], and can potentially allow for the monitoring of endogenous decreases or increases in DNA methylation at single-cell resolution using microscopy or flow cytometry. The reporter system is based on a minimal promoter region that includes the conserved elements between human and mouse of the imprinted gene Snrpn (small nuclear ribonucleoprotein polypeptide). Snrpn was chosen because unlike many other genes, Snrpn is expressed in most if not all tissues regardless of cellular differentiation. This system relies on the demonstration that methylation from sequences adjacent to the insertion sites spreads into the promoter that drives the expression of a GFP. Using the CRISPR/Cas gene editing technology, the RGM system was integrated into endogenous promoters (unmethylated *Gapdh*, methylated *Dazl*) and super enhancer regions (*Sox2*, miR-290), and the RGM system adopted the methylation characteristics of these adjacent unmethylated or methylated genomic regions, demonstrating its potential to monitor methylation. The system can also monitor the demethylation process, as demonstrated by an increased GFP signal following the insertion of the methylated RGM system into the unmethylated *Gapdh* promoter. The functionality of the RGM system was also demonstrated under in vivo conditions in chimeric embryos. Whilst the RGM system is promising for use in screening strategies to identify putative epigenetic regulators [400], application for chemical screening has not been reported to date.

### 8.5. Global Genome Changes in Histone Modifications and DNA Methylation

Indexes of GGDm and GG histone modifications can be measured by multiple methods depending on the availability of samples and of instruments. High-performance liquid chromatography and capillary electrophoresis [454,455] methodologies with various mass spectrometry detection and quantification systems [456,457] are the gold standards because they provide absolute measures of GGDm and histone modifications. The strengths of these methodologies reside in the provision of highly accurate data and addition of assay modifications to simultaneously measure other modified bases of epigenetic relevance [458,459,460].

Whole-genome bisulphite sequencing (WGBS) and reduced-representation bisulphite sequencing (RRBS; [461]) are based on the bisulphite reaction of unmethylated cytosines and next-generation sequencing, and in addition to providing indexes of GGDm, they inform about sequence-specific changes in DNA methylation for further validation [462,463]. Alternative techniques to measure GGD methylation include the pyrosequencing-based luminometric methylation assay (LUMA) method [464], antibody driven techniques followed by blotting, flow cytometry analyses [394], or commercially ELISA kits from multiple companies. Additional methodological resources with respect to DNA methylation assays are documented in Wang and Petronis [465], Esteller [466], and Tost [467].

Liquid chromatography–mass spectrometry (LC–MS) and capillary electrophoresis (CE) systems are antibody free systems and highly accurate in reporting absolute changes in global genome abundance of many HPTM and DNA covalent modifications. Many histone modifications (mono, di, or trimethylation) can be measured within the same sample [468]. Similarly, cytosine modifications (5mC, 5hmC, 5fC, and 5caC) can also be measured within the same sample [469,470,471]. Given that such assays can be performed from paraffin embedded samples [468], or from in vitro and in vivo samples, this permits verification that in vitro screening observations can correlate with in vivo outcomes. In contrast to locus-specific measurements, a major advantage of measuring global epigenomic changes is that it is applicable to any cell types and chemical treatments, but without providing locus-specific information.

Cytosine extension assays were first described in 1999 [472], are based on parallel use of methylation-sensitive (*HpaII*, *AciI*, *BssHII)* and insensitive (*MspI*) isoschizomer restriction endonucleases that leave a 5′-guanine overhang after DNA cleavage, and on their extension using DNA polymerase and labelled dCTP. The wide distribution of the CCGG sequence [8% of CpG sites in the human genome [473]], and measurement of incorporated labelled-C reported as ratio of the isoschizomers products, provide an indicator of the global cytosine-methylation level in a cell or tissue sample. The type of labelled dCTP (radioactive, fluorescent, HRP, biotin labelled) gave rise to a family of assays based upon the same principle [reviewed in [394]]. The luminometric methylation assay (LUMA) developed by Karimi et al. [474] is similar but based on pyrosequencing.

In addition to Western blot and enzyme-linked immunosorbent assay (ELISA), commercially available tools can be used to measure global genome changes in histone modifications from cellular extracts. These may include the AlphaLISA/Alphascreen (PerkinElmer, Waltham, MA, USA) [475,476] and the LanthaScreen systems (Life Technologies, Carlsbad, CA, USA), for which the assay specificity is dependent on the antibody quality [477]. The latter group applied this technology to the high-throughput analyses of H3 modifications, including H3K4me2, H3K4me3, H3K9me2, H3K9 acetylation, H3S10 phosphorylation, and H3K27me3. Machleidt et al. [477] confirmed that hypoxic stress, induced by the iron chelator deferoxamine, increases K4 methylation levels, and that the methyltransferase G9a inhibitor, UNC-0638, decreases K9me2 levels with little effects on other modifications. The K9 acetylation assay was also used to screen effects of the 43 compounds of the Enzo Epigenetics Library (Enzo Life Sciences International, Plymouth Meeting, PA, USA) and identified the modulators for type I/II HDACs but not SIRTs. Other similar assays measure histone acetylation [478], and lysine acetyltransferases [479].

## 9. Markers of Specific Key Events for Consideration in Multiplex DNA Methylation Assays

The list of genes listed in Table 3, together with those discussed here, showed deregulated expression by DNA methylation (either hypo or hypermethylation) during carcinogenesis. These genes are relevant to the key events of the IATA leading to cancer development [10] and are explored in more detail. Some of these genes may be considered for the development of multiplex DNA methylation assays, so that more than one gene can be monitored simultaneously from many samples at reasonable cost (Oku et al. in preparation).

### 9.1. Epigenetic Impairment of Detoxification and DNA Repair Pathway; MGMT, BRCA1, and GSTP1

Promoter hypermethylation and gene silencing of the DNA mismatch repair gene hMLH1, the DNA alkyl-repair gene O(6)-methylguanine-DNA methyltransferase (MGMT), the detoxifier glutathione *S*-transferase P1 (GSTP1) and the familial breast cancer gene BRCA1 were reported to create predispositions to four specific genetic lesions: microsatellite instability, G to A transitions, steroid-related adducts and double-strand breaks in DNA, respectively [480]. Commercially available DNA methylation tests, including two of these genes (*Gstp1*, *Mgmt*), were recently examined for (a) associations between their targeted genomic location with corresponding methylation profiles from The Cancer Genome Atlas, (b) for links with changes in gene expression, and other relevant characteristics [210]. They report that nine assays (*Gstp1*, *Apc*, *Rassf1*, *Ndrg4*, *Bmp3*, two *Sept9* biomarkers, *Shox2*, and *Mgmt*) have been included in one or more clinical guidelines [210]. *Mgmt* is the most clinically advanced epigenetic biomarker to predict the response to temozolomide and carmustine in gliomas [425]. On the basis that defective detoxification and DNA repair mechanisms can be at the origin of carcinogenesis, assays for *Gstp1*, *Mgmt*, and for the tumour suppressor gene *Brca1* (as discussed below), will provide useful data and are worth further consideration in the IATA.

#### 9.1.1. BRCA1, H2AX, and H4K20

A systematic review by Vos et al. [481] reports that *Brca1* promoter hypermethylation is rare in breast and ovarian carcinomas of *Brca1* germline mutation carriers but is relatively more frequent in sporadic cases. In sporadic breast carcinomas and in sporadic ovarian carcinomas, *Brca1* promoter methylation was found in 5.8–35.7% and in 12.3 to 22.5% of the cases, respectively. However, most studies (19/21, 90.5%) were judged as having either a high or unclear risk of bias regarding the methylation analyses due to absence or unclear information on blinding, thresholds and controls [481]. *Brca1* and *Mgmt* promoter hypermethylation are not limited to breast cancer but are observed in other malignancies such as lung adenocarcinoma [482]. Interestingly, sodium arsenite exposure was shown to increase *Brca1* promoter methylation in the human breast cancer cell line MCF7 in vitro [313], supporting that it may be a useful marker.

The relevance of monitoring *Brca1* promoter methylation is better appreciated knowing the function of BRCA1 in DNA repair. DNA double-strand breaks (DSB) can occur as a consequence of replication fork collapse, or following exposure to chemicals or ionizing radiation, or can also occur transiently during DNA repair processes [179]. Broken DNA sites are recognised by the MRE11–RAD50–NBS1 (MRN) complex, which recruits the Ataxia telangiectasia mutated (ATM) and related protein kinases, leading to the phosphorylation of serine-139 in the carboxyl-terminal tail of the H2AX protein. The accumulation of the phosphorylated H2AX forms regions called γH2AX. These γH2AX regions mark the site of DNA damage and provides a nucleation site for the formation of damage response and repair complexes. BRCA1 and BRCA2 are involved in the homologous recombination (HR) error-free repair process of DNA double-strand breaks (DSB). HR can occur during the *S*-phase and G2 phase of the cell cycle due to the presence of homologous DNA in the sister chromatid [180,483]. In contrast, the nonhomologous DNA end-joining (NHEJ) error-prone DNA repair process occurs in all phases of the cell cycle and recruits p53 Binding Protein 1 (53BP1) to DNA damage sites and blocks the HR process [180]. Reduction in BRCA1 abundance favours the error-prone NHEJ pathway, supporting the predominance of the error-prone NHEJ pathway following *Brca1* gene silencing [180]. Many factors can direct DNA repair toward the HR or NHEJ pathway, including the level of methylation of histone H4 lysine 20 (H4K20) that changes across the cell cycle [180,483]. H4K20me2 is abundant during G1 phase and diluted 2-fold during S phase as new unmodified histones are deposited onto nascent DNA. H4K20me2 levels are subsequently restored during the G2 phase. Unmethylated H4K20 is required for BRCA1 (through BARD1, its obligate binding partner) to conduct the HR pathway [483], and to oppose the 53BP1 function that requires H4K20me2 [179,483]. H4K20 is monomethylated (H4K20me) by KMT5A, and di (H4K20me2), and trimethylated (H4K20me3) by KMT5B/C, demethylation of H4K20me2/3 is conducted by KDM4A (alias JMJD2A). It may be speculated that in addition to *Brca1* silencing by promoter DNA methylation, interference with the above histone methylase and demethylase activities can contribute to DNA repair deficiencies.

#### 9.1.2. GSTP1

There are nine classes of glutathione *S*-transferases that can be cytosolic, membrane bound microsomal, or mitochondrial. The function of GST is classically to eliminate by-products of oxidative stress and electrophilic xenobiotics by catalyzing their binding to the tripeptide glutathione (GSH), but also GST can act as intracellular transport proteins for various hormones/proteins, or as modulators of signaling pathways (e.g., JNK/c-JUN, MAPK) [484]. Overexpression of GSTP1 is found to occur in many cancer types [484,485] and in foci as a marker of preneoplastic lesions in numerous tissues in rodents and human [486,487,488], and it is indicative of cellular stress and antioxidant response as observed in an arsenic exposed population [324]. Reduction in GST activity is also of great relevance to carcinogenesis. Gene deletion, single nucleotide polymorphism, and epigenetic silencing, all cause reduction in GST activities that are positively associated with increased DNA damage and incidence of various cancers [484,485]. *Gstp1* is one of the nine genes for which commercially available methylation assays were developed and integrated in clinical guidelines [210]. The promoter of *Gstp1* includes a hypomethylated region at the preneoplastic stage, and later during carcinogenesis these sites get methylated to silence GSTP1 expression, as observed with human hepatocarcinoma [488,489]. Ongoing controversies in the *Gstp1* methylation literature concern the adjacent methylated and unmethylated regions of the promoter, methylation progression during carcinogenesis, as well as differences in technologies and assay designs [490]. Only well-validated *Gstp1* assays should be used (e.g., pyrosequencing [490]) to further test *Gstp1* methylation as a marker in chemical testing strategies.

### 9.2. Cell Cycle Regulation Breakdown, the Ink4b/Arf/Ink4a Locus

DNA damage, aging, and cellular stress (occurrence of reactive oxygen species, oncogene activation, telomere erosion, stalled replication forks [181]), are detected by gene pathways (e.g., telomerase, p53, RB) regulating the transcription of the *Ink4b/Arf/Ink4a* locus, which produces cyclin-dependent kinase inhibitors inducing cell cycle arrest and contributing to senescence and apoptosis to prevent carcinogenesis. In the absence of such a key event relationship, chemical induction of proliferation can contribute to carcinogenesis by permitting the fixation of mutations (and epimutations) in subsequent cellular generations. This section summarises how the *Ink4b/Arf/Ink4a* locus can be epigenetically silenced, and how it can be reported as a measurable key event in the IATA.

Progression through the cell cycle is mediated at the G1, G2, and mitosis checkpoints of the cell cycle by cyclins, cyclin-dependent kinases, and their inhibitors such as p14^Arf^ (p19^Arf^ in mouse), p15^Ink4b^, p16^Ink4a^, p21^Cip1/Waf1^, and p27^Kip1^. The *Ink4b/Arf/Ink4a* locus is an important carcinogenic target as it transcribes four genes mediating activity of the p53 and pRB pathways. The cyclin-dependent kinase inhibitors (CDKI) p14^Arf^ (p19^Arf^), p15^Ink4b^, p16^Ink4a^, and the long non-coding RNA ANRIL (antisense non-coding RNA in the *INK4* locus) are four genes transcribed from the *Ink4b/Arf/Ink4a* locus, considered by some as the most frequently mutated locus (MYC amplifications and *Cdkn2a*/*b* deletions in 14% of 3131 cancer samples [491]), while p16^Ink4a^ is epigenetically silenced in more than 70% of cases, depending on cancer types [492,493]. Upon detection of an oncogenic signal and aging, p16^Ink4a^ is transcribed from the tumor suppressor gene *Cdkn2a* from the *Arf*/*Ink4a* locus to arrest the cell cycle at the G1 checkpoint [181]. P14^Arf^ is also transcribed from *Cdkn2a,* but *Arf* express exon 1α and alternate reading frame of exon 2, whereas *Ink4a* express exon 1β, 2 and 3. P14^Arf^ (p19^Arf^ in mouse) arrest the cell cycle at G1 and G2 phase of the cell cycle and inhibits MDM2 (HDM2 in human) and thus stabilizes p53 [494,495]. P15^Ink4b^ is transcribed from *Cdkn2b* to arrest the cell cycle either at G1 or G2 [496,497]. The long non-coding RNA ANRIL can reduce p15^Ink4b^ expression by recruiting the polycomb repressive complex PRC2 [498].

In the G1 phase of the cell cycle, the RB tumour suppressor proteins are bound to the transcription factor E2F preventing it from inducing transcription of genes necessary to advance in the cell cycle. To move from the G1 to the S phase, cyclin D and cyclin-dependent kinase CDK4/6 form the CDK4/6-cyclin D active complex that can phosphorylate RB, which then releases E2F to induce gene transcription and proteins necessary for cell cycle progression. Under cellular stresses and senescence (replicative, oncogene-induced), p16^Ink4a^ binds CDK4/6 and prevents the formation of the CDK4/6-cyclin D complex, this prevents RB phosphorylation and the release of E2F, and consequently inhibits cell cycle progression. During carcinogenesis the *Ink4b/Arf/Ink4a* locus and cell cycle checkpoints can be deregulated by mutation, homozygous or heterozygous gene loss, or by epigenetic anomalies acting either at the enhancer element upstream of the entire locus or at gene-specific promoters, or by actions of the lncRNA ANRIL [492,499].

Given both the importance and complexity of the *Ink4b/Arf/Ink4a* locus, more than a single assay type together with long-term assays may be required to assess its functionality. p16^Ink4a^ alone, or in association with p14^Arf^ [494] or with p15^Ink4b^ [500] can be epigenetically silenced. In either case, this will lead to the absence of functional *Ink4b/Arf/Ink4a* locus and permit cell cycle progression in the presence of unrepaired genomic and/or other epigenetic anomalies. p14^Arf^ and p16^Ink4a^ are barely detectable in normal cells and can be silenced in cancers, consequently measuring differences in protein expression may not differentiate both situations, but epigenetic silencing of these genes should permit to distinguish normal from epigenetically abnormal tumorigenic cells. The expression and silencing of the *Ink4b/Arf/Ink4a* locus require the interplay of numerous epigenetic enzymes [126,181,492,495], with gene silencing occurring prior to the detection of DNA methylation [501]. Indeed, after growing primary human mammary epithelial cells in serum-free medium for 2–4 weeks, colonies emerge with silenced p16^INK4A^, but it is in the following weeks that the promoter shows gradual de novo DNA methylation and histone remodelling (H3K9 deacetylation and dimethylation) to consolidate its suppression [501]. In other cases, high abundance of p16^Ink4a^ can be found in cells with ongoing senescence or subjected to various stressors, as well as in some cell lines and some cancers when the RB negative feedback exerted on p16^INK4A^ is inhibited as in the case of human papillomavirus (HPV) infection [181,495,502]. The p16^Ink4a^-RB pathway can be deregulated by HPV infections that release the oncoproteins E6/E7 that bind pRB and prevent cell cycle arrest as well as the negative feedback mechanism regulating p16^Ink4a^ expression [181,503]. In this case, the measurement of an indicator of cell proliferation (e.g., Ki67) or inflammation should permit distinguishing between senescent and oncogenic dividing cells with both showing increased p16^Ink4a^ expression [181]. Finally, species differences are important to note in the investigation of the *Ink4b/Arf/Ink4a* locus; cell cycle progression and culture-induced growth arrest are more regulated by p14^Arf^ (p19^Arf^) in the mouse but by p16^Ink4a^ in human [504,505]. Further, ANRIL exons do not exist in the mouse [499]. Overall, despite its complexity, given the importance of the *Ink4b/Arf/Ink4a* locus and that it is found to be frequently epigenetically silenced during carcinogenesis, investigating the DNA methylation “hot spots” of the *Ink4b/Arf/Ink4a* locus [501] may generate informative biomarkers.

### 9.3. Inflammation/Immune Response Disruption

The immunosurveillance system eliminates abnormal cells to prevent cancer development, although some transformed cells can evade immune detection to create cancers [506]. An excellent review was recently published explaining how tumour cells can evade immune detection [507]. The impact of the multiple forms of cellular stress (proteotoxic, oxidative, endoplasmic reticulum, genotoxic, and metabolic stress, and hypoxia) on activation and inhibition of the components of the immunosurveillance system were recently reviewed [181]. Tissues exposed to chemicals can generate immune-stimulating signals due to cellular stress and toxicity, and cells that reach senescence produce the senescence associated secretory phenotype (SASP) stimulating the immune system to expedite their elimination, and extracellular matrix and growth factors to facilitate tissue regeneration. Persistent senescence contributes to chronic inflammation, diseases of aging, and tumour formation [508,509]. In contrast, tumorigenic cells emanating from this environment might generate immune-suppressive signals to avoid immune detection. The duration of treatment and timing of sampling might influence the transformation process and the abundance of cells sending immune-stimulating (stressed or senescent cells) or immuno-suppressive signals. The diverse signals emanating from such an environment complicates the identification of the relevant ones as predictive of adverse effects. Chemical hazard assessment considers mechanisms that reduce the efficiency of the immune system (mostly in animals), and those adopted by transforming cells to avoid or escape immune detection. While immune-stimulating signals are important in toxicological assessment, the adverse outcome in carcinogenesis is the detection of immunosuppressive signals from tumorigenic cells. The latter therefore guides the selection of in vitro epigenetic assays presented here. Important challenges in assay selection are on the one hand the consideration of the complexity of the immune system that involves numerous cell type interactions and differentiation in vivo, and on the other hand the international pressure to phase out animal testing and to develop in vitro chemical testing procedures [510]. While there are epigenetic mechanisms that can be targeted to suggest a reduction in the efficiency of the immune system under in vivo conditions [511,512,513], the current section discusses some epigenetic measurements that can support the acquisition of immune evasion properties during the in vitro transformation of tumorigenic cells.

Tumorigenic cells can escape immune detection through a variety of mechanisms such as the maintenance of an immunosuppressive tumour microenvironment by producing IL-4, -6, -10, -13, TGFβ, and VEGF, by the loss of the expression of a variety of tumour-associated antigens, or by loss of antigen-presenting machinery-related genes, either through genomic instability or epigenetic reprograming [514,515]. Seeding information suggesting a role for DNA methylation in contributing to immune evasion are provided by numerous observations following treatment with DNA methyltransferase inhibitors (DNMTi). DNMTi treatment can increase tumour cell immunogenicity by: (1) re-activating expression of major histocompatibility complex (MHC) components to increase neoantigen presentation, (2) re-activating expression of silenced immune checkpoint (IC) and IC ligands (ICL), (3) increasing the expression of natural killer group 2 D receptor ligands (NKG2DL), (4) or inducing expression of cancer testis antigens (CTAG). Although less related to the immune evasion strategy, it should be noted that treatment with DNMTi can contribute to the elimination of tumour cells by re-activation of hypermethylated tumor suppressor genes, by activating retrotransposons and retroviral elements leading to dsRNA and cytosolic DNA that activate interferon responses [50,368,516]. Regulators of immune evasion, like other genes are subjected to many transcriptional (transcription factors, epigenetic regulators), translational (e.g., miRNAs), and post-translational regulatory mechanisms, offering multiple complementary investigative approaches (e.g., genomic, epigenomic, immunohistochemistry (IHC)). IHC is being used extensively for clinical purposes, but antibody specificity and assay variability introduce difficulties in data interpretation. Considering that immune evasion must be prolonged from the initial stage of transformation until later cancer progression, combined with immune evasion and suppression assays (Corsini et al. in preparation), DNA methylation can be promising robust assays indicative of persistent and long-term changes. The development of multiplex assays for the measurement of DNA methylation changes at multiple loci would offer the advantage of deriving a better coverage of the multiple mechanisms of immune evasion. More details supporting the consideration of developing epigenetic assays related to MHC, CTAG, IC, ICL, and NKG2DL are provided in the following three sections. Note that such approaches are relevant to chemical hazard assessments only when studying target tumorigenic cells, and not tumor samples that include combination of infiltrating immune cells with tumorigenic cells that can express the same molecules, and thus would dilute and complicate data interpretation.

#### 9.3.1. Major Histocompatibility Complex-I and -II

Chemicals that reduce the abundance of cell surface antigens are of concern. To eliminate proteins from invading agents or non-functional endogenous proteins (mutated, misfolded, etc.), the proteasomes degrade proteins into peptides that can be recycled or brought to the cell surface and presented as antigens by components of the major histocompatibility complex (MHC). Components of the MHC-I complex (e.g., human leucocyte antigen HLA-A, -B, and -C) are expressed on nucleated cells and tumour cells, and components of the MHC-II complex (e.g., HLA-DR, -DP, and -DQ) are expressed on cells of the immune system (e.g., dendritic cells, macrophage, B cells) but can also be induced on some tumour cells or normal cell types in inflammatory conditions [517,518]. It is through these mechanisms that abnormal antigenic peptides originating from invading agents or abnormal proteins can be presented by MHC-I at the cell surface and elicit an immune response leading to the elimination of the affected cells by cytotoxic CD8^+^ T cells. The expression of the MHC-I complex can be regulated directly by cytokines such as interferon gamma (INFγ), or indirectly through the MHC-I regulating proteins NLRC5 (nucleotide-binding domain and leucine-rich repeats containing (NLR) family, caspase activation and recruitment domain (CARD) domain containing 5) and CIITA (Class II transactivator), whereas genes from the MHC-II complex are regulated through CIITA [517,519]. Genes from both MHC-I and -II antigen-presenting machinery can either be epigenetically modulated [515,519] or rendered defective by mutations [520]. In both cases, these mechanisms reduce abundance of antigens at cell surface and contribute to immune evasion of the tumorigenic cells.

NLRC5 is a major regulator of the expression of the MHC-I gene complex, including HLA-A, HLA-B, HLA-C, β2m, LMP2, LMP7 and TAP1, which are necessary for the presentation of neoantigens to CD^8+^ T cells resulting in the elimination of cancer cells [521]. The investigation of 21 tumor types [522] and a review by the same laboratory [515] revealed that NLRC5 is the main tumorigenic target of the MHC-I pathway displaying genetic and epigenetic alterations. The DNA methylation level of the *Nlrc5* promoter varies across cancers, but it is the most methylated among MHC-I genes, and its expression is responsive to DNMTi treatment. Consequently, DNA methylation represents the main mechanism of *Nlrc5* regulation, followed by copy number loss, and somatic mutations [515]. The gene *Nlrc5* deserves consideration in chemical hazard assessment.

Some of the NLRC5 target genes of the MHC-I complex are also modulated by epigenetic mechanisms. HLA-A expression is affected by methylation but not HLA-B and -C. Indeed, the investigation of peripheral blood mononuclear cells from healthy donors revealed allelic lineage-specific methylation patterns within the *Hla-a* promoter where increased DNA methylation levels correlated with reduced HLA-A expression, and increased expression following DNMTi exposure [523]. Additional antigen-processing and -presentation molecules that can be regulated by DNA methylation include B2M (beta-2 microglobulin), CALR (calreticulin, a reticulum endoplasmic protein), CD58 (alias LFA-3, lymphocyte function-associated antigen 3), PSMB8, and PSMB9 (Proteasome subunit beta type-8, -9), which were found upregulated following DNMTi exposure in colon and ovarian cancer cell lines [524].

In contrast to HLA expressions which are generally associated with immunogenicity, the HLA-G (isoform G3) expression in colorectal cancer is suggested as a mechanism to escape immune recognition and destruction [525]. The placenta avoids immune reaction through the production of HIF1α-mediated expression of HLA-G and PD-L1 (see Section 9.3.2) [526]. In human cancers the expression of HLA-G is also mediated through HIF1α [527,528]. While exposure to DNMTi increases the expression of HLA-G [528], the exact contribution of DNA methylation in the regulation of HLA-G expression remains controversial [525].

Drugs and environmental contaminants have been shown to contribute to immune deficiency. Substances inducing oxidative stress such as sodium arsenite (≥1 µM NaAs_2_O_3_), or the antioxidant dimethyl fumarate (≥25 µM DMF; autoimmune disease drug) and tert-butylhydroquinone (≥25 µM tBHQ) reduce INFγ-induced (100 ng/mL) MHC-II (HLA-DR) surface protein expression in HeLa and in U118 cell lines and consequently reduce the efficiency of the immune system [529]. The effects of arsenite are mediated by the inhibition of the oxidative stress sensor KEAP1 (which also mediates NRF2-dependent antioxidant gene expression), and inhibition of H4K16 histone acetyltransferase MYST1, both involved in INFγ pathway inducing MHC-II surface protein expression. The antioxidant DMF is a broad regulator of INFγ-induced MHC-II and chemokine expression acting through multiple pathways [529].

Overall, developing and validating multiplex epigenetic assays to monitor functions of the antigen presenting machinery, including epigenetic silencing of *Nlrc5* and MHC-I-related genes (e.g., *Hla-a*) and expression of HLA-G, can be useful to predict predisposition to immune evasion.

#### 9.3.2. T Cell Immune Checkpoints, NK Cell NKG2D Receptor, and Their Ligands

To avoid over stimulation of the immune system and the destruction of normal cells, cell surface proteins are expressed on normal cells to interact with T-cell surface proteins to reduce potential T-cell overactivity. As a mechanism of immune evasion, tumorigenic cells can produce cell surface proteins called immune checkpoint ligands (ICL) (e.g., B7/CD80/86, PD-L1/2, GALECTIN-9, LSECTIN, NECTIN-2, VSIG-3) that interact with proteins called immune checkpoints (IC) (e.g., CTLA-4, PD-1, TIM-3, LAG-3, TIGIT, VISTA, BTLA) located at the surface of T-cells to inactivate them [530]. In clinical practice, interaction between IC and ICL are targeted by immunotherapies to prevent immune evasion. To prevent side effects of unnecessary immunotherapy, immunohistochemistry (IHC) analysis of biopsies is performed to identify the presence of IC and ICL. However, tumor heterogeneity, antibody and assay variability affect the accuracy of IHC analyses [531]. The roles of DNA methylation and histone modification in immune checkpoint regulation (PD-L1, PD-1, CTLA-4, TIM-3, LAG3 and TIGIT) have been reviewed [530], and as expected, DNA hypomethylation and decreases in the abundance of repressive marks (H3K9me3, H3K27me3) are associated with increased expression of these ICL and IC.

PD-L1 (programmed death ligand 1, alias CD274) is an important ICL that activates the inhibitory PD-1 receptor on T cells compromising T-cell functions and contributing to immune evasion. Exposure to DNMT inhibitor increased PD-L1 expression in melanoma cell lines [532], in non-small cell lung cancer cell lines [533], in breast, colon, and ovarian carcinoma cell lines [534]. These data suggest that DNA methylation of the *Pd-l1* promoter prevents its expression, but there are contrasting levels of DNA methylation depending on cell lines and tissue types, which raise the need for careful selection of in vitro experimental strategies in the development of a *Pd-l1* assay that is suitable for the NGTxC IATA. The culture of human breast cancer cell lines (MCF7 and BT-549) for more than 7 days in cancer stem cell medium promotes the formation of tumourspheres associated with increased expression of mesenchymal markers (VIMENTIN, *N*-CADHERIN, SNAIL) as well as PD-L1 [535], supporting that mechanisms predisposing to immune evasion can be monitored in relatively short experiments. During this process, DNA in the promoter of *Pd-l1* gets hypomethylated in MCF7 cells, but the level of DNA methylation in BT-549 remains statistically unaffected, being already at a low level of methylation. Decreases in the abundance of repressive histone marks (H3K9me3, H3K27me3) have been observed in the promoter of *Pd-l1* in tumourspheres from both cell lines [535]. Similar to the BT-549 cell line, the promoter of *Pd-l1* is also unmethylated in pairs of normal tissues and in colorectal [536] and breast cancer samples [537]. Refinement in the analyses of melanomas suggests that the *Pd-l1* gene expression is regulated by two specific CpG sites in the promoter [538]. By comparing 12 melanoma cell lines showing either constitutive or inducible PD-L1 expression, Chatterjee et al. [532] demonstrated that global genome DNA hypomethylation, particularly in intergenic regions and repeated elements, promote constitutive PD-L1 expression, and hence immune evasion. Overall, these experiments suggest that DNA methylation is one of the mechanisms that regulates PD-L1 expression, and in some cases, the promoter is the influential locus but in others, distant regulatory intergenic and intronic loci needs further investigation. A DNA methylation biomarker based on 269 CpG sites was recently proposed to predict success of PD-1/PD-L1 inhibition therapy [539]. Other ICL genes for which promoter methylation regulates their expression include *Ctla4* (cytotoxic T lymphocyte-associated protein 4) [531]. Further investigation of the epigenetic mechanism leading to the expression of PD-L1 and CTLA4 and immune evasion is beneficial for both clinical and chemical safety.

While IC/ICL interactions attenuate T cell functions, in contrast, natural killer (NK) cells can be activated to kill cancer cells through the interaction of their receptor NKG2D (natural killer group 2, member D) with their family of ligands (NKG2DL) produced by cancer cells. The NKG2D receptor is also present in many T cell groups (e.g., γδT, natural killer T (NKT), CD8^+^ T cells) as costimulatory receptor but not directly mediating cytotoxicity [540]. In humans, the NKG2D ligands include the MHC class I-related chain A or B (MICA/B), and UL16-binding proteins 1 to 6 (ULBP1-6, alias retinoic acid early transcripts 1 (RAE-1)) that become expressed in infected or tumorigenic cells ([540], see also Esteban et al. in preparation). Human/murine species differences in the type of NKG2DL should be noted for species extrapolation [541]. Cellular stress (e.g., abnormal proliferation, heat shock, DNA damage, and infections) activates signaling pathways leading to the expression of NKG2DL at the cell surface. However, cells can evade immune detection by proteolytic shedding of the cell surface ligands MICA and MICB by the action of disulfide isomerase (ERp5) and several proteases of the matrix metalloproteinases (MMPs) and ADAMs (a disintegrin and metalloproteinases) enzyme families. It was recently demonstrated that the oncoprotein MITF (Melanocyte-Inducing Transcription Factor) regulates the expression of ADAM10 that cleaves MICA/B allowing cells to bypass NK cell surveillance [542]. Antibodies targeting MICA/B prevent proteolytic shedding and maintain immunoactivity [543]. Epigenetic mechanisms can also contribute to silencing of NKG2D ligand as a mechanism of immune evasion. The HDAC inhibitor valproic acid (24 h, ≥2.5 mM) increases the abundance of NKG2D ligand (MICA, MICB and ULBP-2) mRNAs, surface protein expression, and shedding from pancreatic carcinoma (Panc89) and prostate carcinoma (PC3) cell lines [544]. Others, through the investigation of 10 NKG2D ligands in 7 hepatocarcinoma cell lines found that many ligands were downregulated compared to normal liver samples, which could serve as an immune evasion mechanism [545]. The latter found that among the NKG2D ligands, ULBP1 is both necessary and sufficient to regulate NK cell-mediated cytotoxicity. The silencing mechanisms involved EZH2-induced recruitment of DNMT3A to methylate the *Ulbp1* promoter, consequently, treatment with an EZH2 inhibitor (GSK343) or DNMT inhibitor (5aCdR) increased the expression of several NKG2D ligands [545]. In isocitrate dehydrogenase (IDH) mutant glioma cells (a gain-of-function mutation catalyzing the production of 2-hydroxyglutarate (2HG)), 2HG inhibits α-ketoglutarate-dependent dioxygenases and consequently reduces TET demethylase activities and increases genomic DNA methylation. As a result, the level of promoter methylation for *Micb*, *Ulbp1* and *Ulbp3* is higher in gliomas with mutated IDH than with wild type IDH, and the two primary transcriptionally silenced NKG2D ligands ULBP1 and ULBP3 were responsive to DNMTi [546]. Collectively, these data suggest that histone acetylation and methylation, as well as DNA methylation, are mechanisms involved in NKG2D ligand silencing and immune evasion, and ULBP1/2/3, and MICA/B (in human but not mouse) can be priority markers for further development and validation.

#### 9.3.3. Cancer-Testis Antigen Gene Families as Epigenetic Markers of Carcinogenicity

This section describes how exposure to carcinogens that induce DNA hypomethylation can also induce the expression of cancer-testis antigen genes (CTAGs), supporting potential adverse effects of DNA hypomethylation and thus the use of CTAGs as marker of cancer predisposition. CTAGs belong to a large group of tumour-associated antigens (more than 100 gene families listed in http://www.cta.lncc.br, accessed on 6 October 2021) expressed in transformed cells and in numerous cancers but generally not in normal tissues except during spermatogenesis in the testis and in the placenta (some expressed at <1% of testis levels in pancreas, liver, spleen) [547]. A large number of these genes are located on the X and Y chromosomes, but others are distributed throughout the genome. DNMTi treatment upregulates many CTAGs common to both colon and ovarian cancer [524]. The most frequently expressed CTAGs are NY-ESO-1, SSX-2, SSX-4, MAGE-A1, and MAGE-A3 [547]. The melanoma antigen gene (*Mage*) family originates from more than 60 genes generating two families of MAGE proteins (Type I and II) among which the Type-I (MAGE-A, -B, -C subfamilies) are expressed in cancers through epigenetic deregulation [548]. Type-I MAGE promotes cancer cell survival in various ways: (1) by interacting with ubiquitin system to promote degradation of tumor suppressor proteins (e.g., p53, AMPKα1, ZNF382), (2) by inhibiting cyclin degradation, (3) by acting as transcription regulators [548], (4) by repressing, in the case of other CTAGs (PRAME), retinoic acid signaling thereby inhibiting differentiation [549]. It appears that the carcinogenic roles of these CTAGs outweigh their immunogenicity in the tumorigenic environment. The blood–testis barrier and the lack of HLA class I expression on the surface of germ cells may provide protection from attack by the immune system [547], but the growth of CTAG-expressing tumour cells suggests an immune-suppressive tumour environment perhaps involving mutations or silencing of MHC-I components [520,549]. Despite this, the presence of these antigens in cancer cells promotes the design of epigenetic cancer immunotherapies by which treatment with DNA hypomethylating drugs promote their expression and increases the antigenicity of the tumour [50,547].

DNA hypomethylation is an important mechanism regulating the expression of CTAGs with some events promoting carcinogenesis [50,548,550]. In addition to epigenetic drugs [50], exposure to some carcinogens (*Helicobacter pylori* [551], methylcholanthrene [552], smoking [553,554]) induces expression of CTAGs. Consequently, for adequate consideration of CTAGs epigenetics in chemical hazard testing strategies, time-course analyses are needed following exposure to carcinogenic substances for primary cell transformation, global genome DNA hypomethylation, and CTAGs DNA hypomethylation and expression.

### 9.4. The Cytoskeleton; Relevance to Global Genome Epigenetic Marks, E-Cadherin, MYO10

During carcinogenesis, normal cells undergo changes in morphology as they progress through stages of hyperplasia, metaplasia, anaplasia, dysplasia, neoplasia, epithelial-to-mesenchymal transition (EMT [555]), and metastasis. The dysplastic stage (change in morphology, abnormal mitosis, disorganised cell proliferation with loss of cell polarity, cellular and/or structural atypia) may represent the decisive carcinogenic progression step. Progression across these histological stages requires molecular adaptations, pathway signaling, and epigenetic mechanisms conducive to cytoskeleton modifications, changes in cell-to-cell adhesion, and interactions between the cells and the extracellular matrix [556,557]. These mechanisms involve various structures (e.g., intercellular desmosome, gap, tight, and adherens junctions; extracellular matrix hemidesmosomes and focal contact), interacting protein families (e.g., integrins, actins, connexins, claudins, occludin, catenins, cadherins, fibronectin), and signaling pathways (TGFβ-SMAD3, WNT-β CATENIN, and NOTCH) inducing the expression of transcription factors (e.g., ZEB1, ZEB2, SNAIL, SLUG, TWIST) to downregulate or upregulate the expression of epithelial (e.g., E-CADHERIN, CLAUDINS, OCCLUDIN), and mesenchymal markers (e.g., N-CADHERIN, VIMENTIN, FIBRONECTIN). There is an increasing number of studies on different cancers highlighting epigenetic modifications and changes in gene expression that have already occurred by the stage of dysplasia [558,559,560,561,562], and later during EMT [557]. Therefore, monitoring epigenetic deregulation may assist in better predicting the potential adversity of cellular transformation processes. Below we highlight three examples where pre-existing global genome changes in DNA methylation or histone modifications can create epigenetically permissive conditions for oncogenic activation of gene regulatory elements by transcription factors. Epigenetic mechanisms regulating E-CADHERIN expression are discussed. Finally, the epigenetic system (KMT3A, EZH2, WDR5, and PRMT2) can also directly methylate components of the cytoskeleton to maintain genomic stability [563,564]. Collectively, these mechanisms suggest that deregulation of the epigenetic system may impact cell morphology/cytoskeleton and cell function during early and late key events of carcinogenesis.

Neoplastic cells spontaneously develop an epigenetic state [565] to invade the normal surrounding stroma in a cohesive group of cells, with the “leader” cells being distinct from the cells behind referred to as the “follower” cells [565,566], particularly based on the expression of the filopodia protein Myosin-X (MYO10). Such patterns of distinct cell types participating in invasion supports the polyclonal nature of tumours, as opposed to the idea of expansion of a single clone to form a tumour [567]. Investigating breast [565] and lung cancer cells [567], invasive “leader” cells were shown to be phenotypically and epigenetically different from the non-invasive “follower” cell population. Summerbell et al. [567] demonstrate that leader cells activate the NOTCH pathway by increasing the expression of JAGGED1 (JAG1, a canonical ligand of the cell surface receptor NOTCH [568]), which then induces the expression of the filopodia protein MYO10. Filopodia are membrane protrusions involved in cell adhesion, migration, and invasion in normal and cancer cells. The induced expression of MYO10 by JAG1 is possible only when the promoter of *Myo10* is hypomethylated [567]. Moreover, as these phenotypically and epigenetically different cell types were adjacent to a larger cell population, only imaging systems and flow cytometry analyses could distinguish leader cells from other cell types, on the basis of MYO10 expression.

The DNA hypomethylation of the *Myo10* promoter is one of three examples suggesting that the pre-existing global genome epigenetic background provides plasticity to epithelial transformation by creating epigenetically permissive conditions for oncogenic transcription factor activities. A second example involves enzymes regulating the abundance of H3K36me2. Using a mouse model of pancreatic ductal adenocarcinoma and CRISPR/Cas9 sgRNA to screen epigenetic modifiers, Yuan et al. [569] identified the nuclear receptor binding SET domain protein 2 (NSD2) as the top gene significantly enriched in a subpopulation of cells showing the epithelial marker E-CADHERIN (ECAD+). NSD2 is a histone methyltransferase that dimethylates H3K36 to H3K36me2. In contrast, lysine-specific demethylase 2A (KDM2A) was overrepresented in the ECAD− subpopulation. KDM2A is a demethylase that preferentially targets H3K36me2. Monitoring activities of EMT-transcription factors (TF) and cytoskeleton markers, it was determined that mechanisms regulating the abundance of H3K36me2 underlie the capacity of TF to alter the EMT and the reverse process mesenchymal-to-epithelial transition (MET) [569]. The third example involves the SETD1A/KMT2F that methylates H3K4 (me1, me2, and me3; [570]) and promotes gastric cancer tumorigenesis by enhancing glycolysis [571], and by contributing to EMT regulation [572]. The transcription factor SNAIL controls EMT by downregulating ECAD expression and favours cancer cell’s EMT [573,574]. In gastric cancer cells, Wu et al. [572] found that SETD1A/KMT2F is necessary to reprogramme the *Snail* promoter by H3K4 methylation thereby regulating its expression and indirectly EMT [572].

ECAD, a gene product of *Cdh1*, is a growth and invasion suppressor in some cancers [575] but may also have bimodal oncogenic roles (reduction and increase in ECAD promote invasion, and metastasis, respectively) in other cancers [576,577]. ECAD is a member of one of the nine families of cadherins (calcium-dependent cell adhesion molecules) that are localized at intercellular adherens junctions. Cadherins possess extra- and intra-cellular domains that interact with catenins, providing a link to the actin cytoskeleton and with the WNT/β-CATENIN, PI3K/AKT, HIPPO, and NFκB signaling pathways [575]. Loss of ECAD with increased abundance of N-CADHERIN (*Cdh2*) occurs during EMT [578]. ECAD expression was reported to be silenced by DNA hypermethylation of its *Cdh1* promoter and by TF activation [575]. The transcription factors SNAIL, TWIST, and ZEB1/2 are known to bind the enhancer boxes (E-box) of *Cdh1*, attracting epigenetic complexes and silencing its expression [575], reaching 30% of hepatocellular carcinoma cases with silenced *Cdh1* [579]. Repression of *Cdh1* transcription preceded the subsequent acquisition of methylated CpG sites [580]. In addition to *Cdh1*, other cytoskeleton-related genes are silenced by DNA hypermethylation, e.g., Connexin26 in rat hepatocellular carcinomas is induced by choline-deficient diets [581], and *Cdh13* hypermethylation (CADHERIN-13) correlates negatively with hormone receptor status in breast cancers [582]. The role of ncRNAs in regulating ECAD expression was also previously reviewed [575].

The epigenetic system has “chromatocytoskeletal” activities, modifying the chromatin to alter the function of DNA loci, but also directly targeting the cytoskeleton to regulate the structure and function of microtubules and actin filaments [563,564]. SETD2/KMT3A establishes the H3K36me3 marks on the chromatin, and the α-TUBULIN K40me3 mark on microtubules required for proper chromosome segregation and genomic stability [563]. EZH2, WDR5, and PRMT2 as other chromatin HMT also methylates cytoskeleton components [563].

### 9.5. Senescence Bypass and Telomerase Reverse Transcriptase (TERT) Regulation

Cellular senescence is a state of “irreversible” growth arrest leading to quiescence and cell death. Normally, the prevalence of senescence increases with aging and protects against the development of cancer cells [583,584]. There are different forms and origins of senescence: replication-induced senescence observed with aging and associated with telomere shortening with increasing number of cell divisions; oncogene-induced senescence due to oncogene activation; and senescence induced by exposure to substances when sufficient cellular injury and DNA damage are created. However, senescence can be reversible as a rare event. Senescence bypass or the ability of a cell to evade senescence and to achieve replicative immortality usually by activation of the telomerase enzyme are essential steps toward cancer development [56] (Vaccari et al. in preparation).

Telomeres are terminal structures at the end of chromosomes including kilobases-long double-stranded DNA tandem repeats (TTAGGG) followed by a 3′ single-stranded-DNA that forms a telomere loop (t-loop) covered by SHELTERIN-protein complexes [585]. The telomeres and the SHELTERIN components permit DNA replication by preventing chromosomal ends from being mistaken as sites of DNA damage. However, each cell division is associated with telomere attrition that impose a limit in the number of cell divisions that a cell type can accommodate [586,587]. Telomeres that are eroded activate the p53 and RB tumor suppressor pathways (including activation of the *Ink4a/Arf* locus) inducing senescence and cell death, which prevent cancer development. If these pathways are defective when telomeres are eroding or if SHELTERIN components (a six-protein complex) are lost [585], this can lead to a telomere crisis by which DNA repair is activated. When proliferation continues this leads to telomere ligation among chromosomes, dicentric chromosome formation, genomic instability, aneuploidy, chymotrypsis, kataegis, and cancers [586,587,588,589].

The telomerase is a ribonucleoprotein multicomponent enzyme that maintains the telomeres. Activity of the telomerase is undetectable in most normal cells and tissues limiting the cell’s lifespan; however, telomerase activity is present in cancer cells providing replicative immortality. This telomerase enzymatic complex includes a range of associated proteins, RNAs, and a rate-limiting component which is the telomerase reverse transcriptase (TERT) [421,587], and TERT expression appeared early during tumorigenesis in vivo [590]. Dogan and Forsyth [421] report methylation and mutation of the *Tert* promoter in 53% and 31%, respectively, of TERT expressing cancer cell lines.

The epigenetic mechanisms (DNA methylation, histone modifications, and non-coding RNAs) regulating the expression of TERT were recently reviewed [421,591]. A role for DNA methylation in regulating TERT expression was initially controversial; however, it became clear that the TERT Hypermethylated Oncological Region (*Thor*) located from −649 to −217 nucleotides from the transcription start site can regulate the expression of TERT by DNA methylation [421,592]. In contrast to the usual relationship of hypermethylation that silences gene expression, hypermethylation of the *Tert* promoter increases TERT expression by preventing binding of the transcriptional repressors WT1 and CTCF [421]. As expected, abundance of H3K4me1/me3 and H3K27ac increase, and H3K27me3 and H3K9me3 decrease with augmentation and diminution in TERT expression, respectively. Additionally, a number of ncRNAs (let-7g-3p, and 17 miRNAs) are involved in regulating TERT mRNA abundance [421]. Telomere length can be regulated by the expression of the telomerase but also by the Alternative Lengthening of Telomeres (ALT) pathway under the influence of the long non-coding RNA TERRA (Telomeric Repeat-containing RNA) [593,594,595]. The subtelomeric regions represent transition regions between the telomere and the chromosome-specific region. Human subtelomeres are CpG rich and include promoters for TERRA which expression is repressed by DNA methylation. It has been recently proposed that TERRA expression contribute to DNA replication stress and DNA damage in the telomeres which activate their repair and elongation through the ALT and break-induced replication mechanisms [594]. In the context of chemical carcinogen testing strategy using DNA methylation, it would appear that an increase in telomere length favouring replicative immortality might by associated with hypermethylation of *Thor*, or hypomethylation of *Terra* promoters.

While these epigenetic mechanisms can contribute to telomere length and replicative immortality, a series of genetic mechanisms can lead to the overexpression of the telomerase gene to maintain telomere lengths [596]. This includes (1) point mutations in the promoter that lead to de novo transcription factor binding; (2) linking TERT to active regulatory elements elsewhere in the genome; (3) insertions of viral enhancers upstream of the gene; (4) increased dosage through chromosomal amplification, and (5) the ‘alternative lengthening of telomeres’ (ALT) pathway in which telomeres are lengthened through homologous recombination, mediated by loss-of-function mutations in the *Atrx* and *Daxx* genes. Overall, activation of the telomerase activity by genetic or epigenetic events can be used to predict chemical-induced carcinogenesis.

### 9.6. Angiogenesis and Thrombospondin-1

The further development of a tumour is dependent on angiogenesis, the development of blood capillaries to support tumour growth. This process is initiated when the tumour reaches 1–2 mm [597,598]. Thrombospondin-1 (THBS1) plays multiple roles in carcinogenesis, one being as an inhibitor of angiogenesis [599], therefore mechanisms that decrease the expression of THBS1 favour angiogenesis. DNA hypermethylation of the *Thbs1* promoter was shown in 50% of brain tumours and reported to regulate its expression in two cell lines (SW1783, T98G) derived from glial brain tumours [600]. The mechanisms leading to the repression of THBS1 appear to be cell type specific, involving the RAS-PI3 KINASE-RHO-ROCK-MYC cascade or the p53 and pRB pathways in transformed epithelial cells or fibroblasts, respectively [601]. Consequently, DNA methylation of the *Thbs1* promoter might provide an epigenetic approach to detect initiation of angiogenesis as a progressive step in carcinogenesis.

### 9.7. The Homeobox (HOX) Genes

The expressions of homeobox (*Hox*) genes are deregulated in cancers where they act as oncogenes by sustaining cell proliferation and controlling cell differentiation [602,603]. Le Boiteux et al. [604] recently shed light on the role of epigenetic marks in regulating the expression of *Hox* genes in brain cancer. The *Hox* genes are divided into four clusters (A, B, C, D) located on different chromosomes from which transcribed transcription factors and non-coding RNAs are normally involved in embryonic development. Le Boiteux et al. [604] report that the DNA sequence of *Hox* gene clusters are hypermethylated and silenced (except for *Hoxd1* and *Hoxd-As1*) in normal brain samples, but among the 57 HOX transcripts (39 sense and 18 antisense) a median of 37 transcripts was deregulated in the HOX clusters from the isocitrate dehydrogenase (IDH) wild type glioma samples. (Note the IDH mutation status is considered in the WHO classification of glioblastoma with the wild type form present in 95% of glioblastoma [605] (IDH in Section 2.3 and Section 9.3.2). They observed that a subset of *Hox* gene transcription start sites escapes DNA hypermethylation and was associated with loss of H3K27me3 but enrichment in H3K4me3 and H3K9ac. Among these epigenetic changes, the loss of H3K27me3 along the four HOX clusters best predicted the cluster transcriptional activities [604]. Epigenetic deregulation of *Hox* clusters can identify the mechanisms to explain changes in expression of numerous transcripts.

## 10. Transcriptomic Biomarkers and Genome-Wide Signatures

### 10.1. Transcriptomic Biomarkers

Transcriptomic analyses revealing differential expression of epigenetic driver genes might provide epigenetic markers of early carcinogenic events. This approach would be similar to the transcriptomic TGx-DDI [428,606,607] and TGx-HDACi [432] biomarker assays discussed in Section 5.3. In contrast, a transcriptomic response of epigenetic driver genes might be induced only when there is sufficient tissue-specific magnitude of “cellular stress” that limits the availability of cofactors and intermediate metabolites (SAM, acetate, phosphate) necessary for epigenetic machinery functioning. Consequently, duration of exposure, timing of measurement, and tissue/cell type, may differ from those of the TGx-DDI which is based on a unifying cell response. Not all exposed tissues develop cancers and the epigenetic response of a target tissue developing a cancer differs from a non-target tissue [608]. Exposure to chemicals (GTxC or NGTxC) can induce epigenetic effects either as adaptive or as toxic responses, or as early pro-carcinogenic steps [609]. Therefore, given this diversity of mechanisms [610], the discovery at a specific time-course of an epigenetic signature for NGTxC may require investigation in more than one cell type. Overall, a complementary data source of multigene biomarkers based on the epigenetic genes that are drivers of carcinogenic transformation in human cells [179] would be an important weight of evidence component, whether the molecular initial events are due to GTx or NGTx mechanisms, or a combination of both.

### 10.2. Transcription Factors, Enhancers, and Other List of Endpoints for Targeted NGS Assay Development

A large number of genes and their components can potentially be epigenetically reprogrammed and thereby contribute to cell transformation. DNA methylation changes can occur in lists of enhancers, super-enhancers (https://academic.oup.com/nar/article/44/D1/D164/2502575, accessed on 23 August 2021), promoters, associated with changes in gene expression of tumour suppressor genes, oncogenes, imprinted genes (http://geneimprint.com/site/genes-by-species, accessed on 16 July 2021). Consideration must also be given to the transcriptome of stem cell transcription factors (e.g., Yamanaka reprogramming factors OCT4, SOX2, KLF4, cMYC [611], and NANOG) that induce epigenetic remodelling and cellular differentiation. Epithelial-to-mesenchymal transition factors (http://dbemt.bioinfo-minzhao.org/tutorial.cgi, accessed on 23 August 2021), non-coding RNAs (http://rnacentral.org [612,613], accessed on 23 August 2021), and homeobox genes, can contribute to chemical-induced cell transformation and carcinogenesis. Note that in some cases, methylated cancer biomarkers demonstrate increased expression, not reduced expression (SHOX2, OTX1, and ONECUT2 [210]). Recent approaches to generate multiplex assays based on gene expression data [614] could be generated based on RASL-Seq methodology [615], or the TempO-Seq methodology [614,616].

## 11. Technical Considerations for Improving the Regulatory Value of Epigenetic Data

### 11.1. DNA Methylation Assay Robustness and Performance Comparisons

A simple chart, or algorithm, has been developed to facilitate the selection of methods for analyses of DNAm based on needs, costs, and assay robustness [617]. However, the diversity and numbers of techniques to measure epigenetic endpoints have been expanding. A caveat regarding available commercial assays is that when questionable performance is noted, it is often problematic to clearly investigate and correct given proprietary issues. For the purposes of the NGTxC IATA, it will be essential to show transferability and reproducibility of test methods selected.

Different methods targeting the same endpoint were demonstrated to generate different results, for example, effects of phenobarbital on *L1* DNAm could not be detected by methylation-specific PCR (MS-PCR) but were detected with pyrosequencing [201]. A group of 18 laboratories compared a series of 10 clinically relevant methods to measure absolute and relative DNAm abundance in 27 predefined genomic regions as well as global genome DNAm [48]. They found that amplicon bisulphite next-generation sequencing (ABS) and bisulphite pyrosequencing as the best methodologies (pyrosequencing technical correlation within lab (*r* = 0.996) and across lab (*r* = 0.98)) [48]. They observed that relative methods (MethyLight, MS-PCR) can report methylation in opposite direction, while methylation-specific melting curve analysis (MS-MCA), and methylation–sensitive high-resolution melting (MS-HRM), detected fewer expected differences in methylation. They also indicated that MS-MCA, MS-HRM, and MS-PCR should only be used for qualitative comparisons of fully methylated regions (not for heterogeneously methylated regions). Only ABS and pyrosequencing were able to cope with low amount or fragmented DNA. High-performance LC–MS found to be the best method for global DNAm, while immune-quantification was not reliable, repetitive DNA elements did not correlate well with expected differences in global DNAm. Others demonstrated the reliability of pyrosequencing and MALDI-MS analyses compared to other techniques (Methylight, MS-PCR, bisulphite-seq) in measuring DNA methylation of the *Gstp1* promoter [490]. NGS permitted to develop methods (e.g., simultaneous targeted methylation sequencing (sTM-Seq)) to measure multiple DNAm sites in multiple samples simultaneously [618] which can be instrumental in screening series of targeted genes for epigenetic disruption.

Beck [212] reviewed studies that compared the performance of 6 methods to measure genome-wide DNA methylation, five are sequencing (seq) based and one is array-based. MethylC-seq, reduced-representation bisulphite sequencing (RRBS), and the Infinium-27K bead-array, all use sodium bisulphite converted DNA, whereas methylated DNA immunoprecipitation sequencing (MeDIPseq), methylated DNA capture by affinity purification (MethylCap-seq) and methylated DNA binding domain sequencing (MBDseq), rely on capture of methylated DNA by a monoclonal antibody or by the recombinant methyl-binding domains of MECP2 or MBD2, respectively. The methods using sodium bisulphite conversion have high resolution (1 bp) while the capture methods have low resolution (≥100 bp). The overall concordance across methods was high (84–100%), and it was concluded that all evaluated methods are capable of producing accurate high-content data [212].

The performances of “rapid multiplexed” reduced-representation bisulphite sequencing (rmRRBS) performed on the Illumina HiSeq2500 sequencing platform were compared to analyses on the Illumina Infinium BeadChip Human Methylation450 (450K), and MethylationEPIC (850K) platform [211]. Preferences were expressed for NGS-based rmRRBS, but consideration should be given to experimental needs; (1) rmRRBS requires only 60–200 ng DNA, whereas the Infinium BeadChip arrays require 500 ng–1 µg and whole-genome bisulphite sequencing (WGBS) requires 3 µg of DNA; (2) rmRRBS allows for genotyping and the detection of single nucleotide polymorphisms (SNP), which incidentally were found to influence allele-specific methylation of the *H19* imprinted region [211]; (3) rmRRBS offers greater flexibility with the potential to investigate sites that are not interrogated by Infinium arrays; (4) rmRRBS covers more CpG loci and greater abundance of “CpG shores” and “open sea regions”. However, with abundant DNA samples, Infinium arrays were reported to provide consistency in both genomic coverage and methylation estimates. In contrast to array-based approaches, many methodological details can induce NGS inconsistencies in methylation analyses across experiments [211]. These include number of reads covering each CpG sites, consistency of the enzymatic digestion and fragment size, fragment ligation to adapters, bisulphite conversion, PCR amplification, successful sequence alignment, and DNA input quantity and quality. Finally, the bioinformatics step, including CpG site grouping of various size rather than investigating individual CpG sites will influence the results based on the heterogeneity of the methylation levels across sites and density of CpG sites. Examples of other methods exist, including EpiTYPER^®^ (a mass spectrometry-based bisulphite sequencing method that enables region-specific quantitative DNA methylation analysis; evaluated by [48]), Illumina’s VeraCode GoldenGate customizable assays, and newer techniques (PacBio, single-molecule real-time (SMRT) sequencing, nanopore sequencing), all of which are still to be thoroughly evaluated, together with recent bioinformatics applications [619].

Overall, pyrosequencing and NGS-based techniques offer versatile approaches for investigating DNA methylation, with due consideration of the technical differences and limitations within and across studies. Different parameters (such as “window” selection process, CpG density, methylation threshold cut-off; see for example the video from Dr. Simon Andrew at Babraham Institute: https://www.youtube.com/watch?v=MWZ8dBpOpkM, accessed on 19 July 2021) can be selected to identify differently methylated regions (DMR) that can influence the interpretation of methylation calls as biological observations. Of note is the supplementary Data 1 section in [48], which provides validated DNA methylation protocols.

### 11.2. Methodology to Distinguish 5mC from 5hmC

5mC, its oxidised derivative 5hmC, and histone post-translational modifications contribute to the regulation of chromatin structure and function [620,621]. The various methods that use sodium bisulphite to identify CpG methylation sites have been instrumental in revealing the importance of DNAm, but these methodologies cannot distinguish 5mC from 5hmC. The following discussion summarises the importance of 5hmC and the methodologies to separate both forms of cytosines.

As previously mentioned, the TET enzymes oxidize 5mC into 5hmC which is a demethylation intermediate and an epigenetic mark with functional roles [94]. As an intermediate, 5hmC can lead to active and passive demethylation. The active process involves further oxidation by the TET enzymes of 5hmC to 5-formylcytosine (5fC) and 5-carboxylcytosine (5caC) that are then actively removed by thymine-DNA glycosylase and the base excision repair mechanism. 5hmC also leads to passive demethylation because 5hmC is not recognized by UHRF1, which prevents the formation of the DNMT1/UHRF1 complex on the hemimethylated DNA, and therefore the complex cannot restore 5mC on the nascent strand at the replication fork. 5hmC has biological roles, despite the fact that it is approximately 10-fold less abundant than 5mC. It is more abundant in the brain and embryonic stem cells than in other tissues and is preferentially distributed in euchromatin at functional regions of the genome in enhancers, promoters and gene bodies [94]. Its roles are mediated by many binding proteins “readers”. These include, (1) the protein HMCES (5-hydroxymethylcytosine binding, embryonic stem cell-specific protein), which is a DNA lesion protein that senses abasic sites in single-stranded DNA at the replication fork and shields the lesion from error-prone DNA repair processing, thus preserving genome integrity [622]. (2) The methyl binding protein MBD3 (methyl binding domain-3) preferentially binds 5hmC and attracts the DNA demethylase TET1 required for the maintenance of 5hmC, or alternatively MBD3 binds the NuRD complex regulating gene expression in cell differentiation and cancers [623]. (3) UHRF2 (Ubiquitin-like, containing PHD and RING finger domains 2), which preferentially binds 5hmC, but unlike UHRF1, UHRF2 also binds 5mC and contributes to methylated H3K9. UHRF2 has ubiquitination but also oncogenic or tumour suppressive roles depending on cancer cell types [623]. (4) As a final example, 5-methylcyctosine binding protein 2 (MeCP2) is abundant in post-mitotic neurons where it contributes to gene repression and brain functions [624]. MeCP2 has a high affinity for 5mC but low affinity for 5hmC, consequently the accumulation of 5hmC in transcribed genes replaces the high-affinity 5mC binding sites for MeCP2, thus decreasing occupancy and repressive effects of MeCP2 [625]. Overall, 5hmC is an important epigenetic mark with roles in neuronal physiology, cell differentiation, and diseases of aging including cancer [625]. However, it is only relatively recent that techniques were developed to distinguish sites with 5mC from 5hmC [626], and the number of technical approaches is still increasing [627].

Considering (1) that 5mC and 5hmC have different biological roles, (2) that the activities of the dioxygenase TET enzymes are sensitive to numerous metabolic factors (Vit-C, pH, intermediate metabolites) possibly modified by chemical exposures, (3) that TET1 and TET2 are downregulated in many cancers, and (4) that there is generally a global loss of 5hmC in cancers [94], perhaps adequate measurements of both 5mC and 5hmC could generate early markers of chemically induced carcinogenesis. A number of techniques have been developed to examine the position of modified C within DNA sequences, and Liu and collaborators [627] present a summary of these approaches. All techniques involve the comparison of the known in silico DNA sequence to the sequences read in two different aliquots of the same samples; one to infer the 5mC position the other the 5hmC. One approach involves analyzing an aliquot treated with only sodium bisulphite (BS) and comparing the results from a second aliquot receiving two treatments: an oxidizing treatment with KRuO_4_ that transforms 5hmC to 5fC and then a SB treatment (referred to as oxBS technique [626]). In the first aliquot, the BS treatment converts cytosines (C) to uracil (U) but leaves 5mC and 5hmC intact, therefore following amplification of BS-treated samples C, 5mC and 5hmC become T, C, and C. In the second aliquot, following the KRuO_4_, BS, and amplification treatment, the original C, 5mC and 5hmC, become C, 5mC and 5fC, then U, 5mC, and U, and finally T, C, and T, respectively. The comparison of the final sequence in both aliquots permits to infer the position of all modified C. Another approach protects 5hmC by glucosylation prior to TET enzyme mediated oxidation and then followed by BS treatment (TAB-Seq; [628]). A combination of these techniques but replacing SB by a borane reduction treatment generates the technique called TET-assisted pyridine borane sequencing (TAPS), or TAPSβ when a β-glucosyltransferase step is included to protect the 5hmC, or finally, CAPS when the initial oxidation is performed chemically (KRuO_4_) [627]. The borane reduction, as a replacement to BS, does not affect C and avoids issues of a harsh BS reaction and variable conversion efficiencies of C. Tierling et al. [629] recently compared the performance of commercially available BS kits, and proposed a revised BS protocol to discriminate 5mC from 5hmC. A combination of the above techniques can infer the position of C, 5mC and 5hmC, but given the low amount of 5hmC and rates of false positive calls (although limited) among approaches, the NGS strategy requires a higher number of reads to assess abundance of 5hmC [626]. The increasing number of investigations of modified C using alternative techniques (LC/MS-MS [470], biological settings [469,470,471], and multiplex methodology [618], should collectively facilitate the reduction in ambiguity in the interpretation of the role of DNA methylation in carcinogenic processes.

### 11.3. Extrapolation of Experimental Models to Human

In the absence of clear human data, experimental animal and cell culture models are essential in toxicology but a perfect model does not exist, and a careful examination of species differences and in vitro confounding factors must be considered. The case of the rodent cancer bioassay highlights difficulties in extrapolating findings to humans [630], considering, for example PPARα and CAR-mediated carcinogenicity ([341,631,632], further explored in Esteban et al. (in preparation)). Animal experimental models offer several advantages; they can support suspected epidemiological adverse outcomes and can generate awareness of potential health issues. Despite improvement of in vitro models, and of in silico tools such as quantitative structure–activity relationship (QSAR) and toxicokinetic models [633,634], currently, chemical hazard assessment practices are still dependent on in vivo data to confirm adverse effects of mixtures, data-poor chemicals, and metabolites, to generate guiding kinetic parameters for dose–response differences on absorption, distribution, metabolism, and excretion of substances, and identify tissues where substances accumulate and become toxicity-targets. Moreover, developmental and long-term effects cannot be predicted by current in vitro or in silico methods [206,635]. On the toxico-epigenetic discovery side, models from bacteria to mammals have been instrumental in elucidating epigenetic mechanisms [31,636,637], such as the requirement of H3K9 methylation to direct *de novo* DNA methylation by DNMT3A/B [33,224]. Data derived from *C. elegans* and *Daphnia* [638], and a growing library on zebrafish (*Danio rerio*) [639] are informative and represent reductions and refinements in the uses of animals. However, interspecies epigenetic differences, including the distribution of epigenetic marks and repeated sequences that exert regulatory roles, differ across genomes of human, non-human primates [225,226], and other laboratory models [227], impeding direct extrapolation of findings [3]. Pending suitable alternatives, while in vivo models remain fundamental in characterizing chemical hazards for complex NGTxC endpoints, the relevance of in vivo and in vitro toxicology and epigenetic models to human health risk assessment requires careful species-specific mechanistic considerations.

### 11.4. Considerations in the Extrapolation from In Vitro Cultures, Cell Lines and Cell Types

The use of cell lines has many limitations in supporting early epigenetic events in carcinogenesis. Particularly as many commonly used cell lines are already at an advanced transformation stage, and additional chemical-induced transformations may be dependent on pre-existing genetic and epigenetic anomalies. Even when a cell line originates from a biopsy of “normal” tissues adjacent to tumours, these cells are epigenetically abnormal [640] (see Figure 6). Cell lines have overcome senescence and have proliferated in vitro as a result of induced or spontaneous transformation involving genetic and epigenetic changes [641]. Many cell lines undergo genetic drift that may induce interlaboratory variability [642], and reproducibility issues through time. Epigenetics and gene expression profiles differ depending on the gene initially mutated that drives the early carcinogenic process [232], hence these activated pathways may affect cellular responses when testing chemicals. Consequently, data related to effects of chemical exposure using cell lines may reflect anomalies in this cell type and may therefore not be relevant to chemical-induced mechanisms in normal cells. Despite these many limitations, cell lines, whether “normal” in origin, or transformed, remain useful tools for the investigation of specific questions associated with later stages of carcinogenesis, especially when they can be used to independently confirm results.

Horvath et al. [643] indicate that in vitro cultures are under artificial conditions frequently characterised by high glucose, abundant growth factors, high oxygen partial pressure (20% O_2_ in incubator air compared to physiological level of 1% to 14% dependent on tissues), mechanical resistance exerted by culture flasks (plastics, glass), inappropriate extracellular matrix, absence of multi-cell type interactions, large concentrations of serum frequently from a species different from the investigated cells, and with unknown constituents such as ascorbic acid (Vit-C) concentrations. Human cells are unable to synthesize Vit-C and dioxygenase enzymes are dependent on Vit-C as cofactor. Some of these enzymes include TET1-3 (oxidizing 5mC to 5hmC to 5fC to 5caC), JMJC domain-containing histone demethylases, prolyl and asparagine hydroxylases regulating HIF1 stability and normoxic condition [97]. A concentration–response study of Vit-C in culture medium with the human colon cancer HCT116 cell line showed re-expression of p21 (CDKN1A) at 50 µM, increased abundance of 5hmC at concentrations up to 100 µM with no effects on 5mC by LC/MS-MS, and cytotoxicity above 1 mM [644]. Vit-C median human plasma concentration of 53 µM (range of 16–89 µM) has been reported [645]. Amounts of Vit-C present in human and rat tissues can vary greatly (≈2 and 8-fold more abundant in rat plasma and testes, respectively, than in man [646]). The epigenetic system can change rapidly under in vitro conditions. For example, mouse embryonic fibroblasts (historically named “embryonic” but in fact derived from fetus) show erasure of global 5hmC within three days (partially rescued by addition of Vit-C), followed by gains of 5mC in specific gene promoters within seven days of culture initiation [100]. Whilst optimal culture conditions may differ between species, they need to be optimised for specific, reproducible epigenetic investigations, both for initial screening and subsequent mechanistic studies.

Human primary 3D culture model systems that are closer to the human tissue environment are encouraged [643], but the successful development of systems for regulatory purposes is more difficult than standard in vitro tests. The comparison of 2D and 3D in vitro cultures suggests that there is less of a gap between 3D data and in vivo conditions than with usual 2D systems [647]. Cell line data showed that the expression of the lncRNA HOTAIR in breast cancer cells (MDA-MB-231 and Hs578T cell lines) is lower in 2D than in 3D systems, and that the 3D culture method generates a different isoform [648]. The use of pooled human primary cells from multiple donors to create 3D cultures that are metabolically competent with normal karyotype and intact DNA repair system is gaining momentum to replace cell line analyses. For example, using such a hepatocyte 3D-culture system, 5-day exposure to valproic acid at a non-toxic but steatotic 15 mM concentration, followed by three-day withdrawal, led to persistent differentially methylated regions in 31 genes [649], with persistent disruption of energy metabolism [650]. The culture of pools from multiple donors of human primary cells might be a promising avenue for epigenetic testing, provided data reproducibility.

Finally, some epigenetic changes are associated with phases of the cell cycle, or with proliferation rate and seeding density, and may bias effects due to chemical exposure. The H3K27me3 mark (but with less impact on H3K9me3 or on H3K14ac) in breast cancer subtypes can be influenced by the proliferation rate, as determined by correlation with proliferation rate marker Ki67, or by the analyses of cell samples synchronized at the G2-M versus G1-S phase of the cell cycle [34]. Seeding HepG2 cells at low density (21,000 to 42,000 cells/cm^2^) compared to high density (63,000 cells/cm^2^) led to a transient 10% drop in methylation of *AluYb8* after 120–144 h of culture, but such an effect was gene and cell type dependent [176]. It is therefore important to document cell culture conditions and to remain vigilant in the interpretation of data.

## Figures and Tables

**Table 1:** Technical considerations for inclusion of epigenetic data in chemical hazard assessment.

**(1) Sufficient evidence to be considered as ca ontributor to a carcinogenic MIE or KE.**	
**(2) Assay robustness and reproducibility across users and laboratories.**	
**(3) Dose/concentration–response:**	
	Large dynamic range. Threshold concentration to categorize test chemicals as positive or negative. Multiple doses to identify point of departure (PoD): lowest and no observable adverse effect levels (LOAEL and NOAEL), or benchmark dose (BMD). Effective concentration relative to the apical endpoint.
**(4) Protocol:**	
	Exposure duration and chemical withdrawal, to distinguish between transient, adaptive, and adverse reprogramming. Sufficiently long duration of cell culture allowing the cells to proliferate to ensure that the epigenetic change has been inherited across cell generations. Dual in vivo and in vitro assays ability to confirm the relevance of in vitro findings for extrapolating to the in vivo situation. Highly detailed protocols, see template in [10].

**Table 2:** Comparison of epigenetic mechanisms altered by arsenic, nickel, phenobarbital, irradiation, acrylamide, 2-acetylaminofluorene, 1,3-butadiene, furan, and methapyrilene.

Arsenicals	References
(1) Oxidative stress and arsenic metabolism that limit the availability of SAM for DNMT and other MT.	[233,234]
(2) Reduction in DNMT mRNA expression and interference with DNMT activities.	[235]
(3) Global genome DNA hypomethylation and site-specific changes in DNAm including retrotransposons.	[236,237,238]
(4) Reduction in CTCF binding to the DNA. CTCF act as a repressor, insulator, or as a transcription factor.	[239]
(5) Binding of sulfhydryl groups of cysteine residues and displacement of zinc ions from the zinc finger DNA binding domains of numerous proteins (TET, histone acetyltransferase, etc.).	[240]
(6) Interference with TET enzyme expression and activities	[189,190]
(7) Change in DNAm inducing changes in ncRNAs (miRNAs and LncRNAs).	[241,242]
(8) Imbalance in expression of histone variants.	[243]
(9) Histone post-transcriptional modifications	[244]
(10) DNA repair mechanisms	[245,246]
(11) Mitochondrial biogenesis and mitochondrial DNA copy number.	[247,248]
**Nickel**	
(1) Reduction in core histones acetylation.	[249,250]
(2) Redistribution of the silencing mark H3K9me2 and 5mC (p16 silencing).	[251,252,253]
(3) Reduction in the histone methyltransferase activities of G9a (targeting H3K9 dimethylation) and Suv39h1 (targeting H3K9 trimethylation).	[254]
(4) Inhibition of the lysine demethylases; KDM3A/JMJD1A acting on H3K9me1 and me2, while KDM4A-D/JMJD2A-D on H3K9me2 and me3.Nickel ions inactivate 2-oxoglutarate-dependent dioxygenases by replacing the cation Fe^2+^ at the catalytic sites (effects of hypoxia and oxidative stress).	[255,256,257,258]
(5) Inhibition of other dioxygenases, including TET1, HIF prolyl hydroxylases and the DNA repair enzyme ABH2.	[71,255,259]
(6) Deregulation of the ubiquitination/deubiquitination machinery.	[260,261]
(7) Interference with the Zn^2+^ finger protein CTCF.	[262,263]
(8) Long-term effects on gene expression associated with abundance of H3K4me3 and H3K27me3 in promoters.	[188,263]
**Phenobarbital**	
(1) Induction of the stress-response protein GADD45, as mediator of DNA demethylation.	[90,91,264]
(2) 5hmC precedes global DNA hypomethylation, and global loss of 5hmC as an early marker of hepatocarcinogenesis and genome flexibility.	[264,265]
(3) DNAm-dependent expression of miRNAs and LncRNAs from the imprinted *Dlk1-Dio3* locus.	[266,267]
(4) Reduction in hepatic expression of epigenetic system components, and *L1* ORF1 hypomethylation in carcinogenic target tissue only (in the liver but not in the kidney).	[201]
**Irradiation**	
(1) Persistent GGDHo and in distant non-target cells.	[137,268]
(2) DNA hypomethylation of more recent *L1* retrotransposons	[137,268]
(3) Over expression of DNMT3B contributing to p53 and p21 silencing by DNA methylation.	[269]
**Other chemicals (acrylamide, 2-acetylaminofluorene, 1,3-butadiene, furan, and methapyrilene)**	
Carcinogen target tissues show decreases in abundance of H4K20 methylation and of its corresponding histone methyltransferase family (KMT5A/B/C), compared to non-target tissues.	[270]
**Estradiol-17β**	
DNA hypermethylation of the distal promoter of catechol-*o*-methyltransferase that reduces the expression of this protective enzyme against the formation of the mutagenic catecholestrogens.	[271]

**Table 3:** Epigenetic assay types and measurement/marker approaches proposed for adaptation for chemical hazard assessment.

**1. Screening assays for chemical interference with enzyme activities:**
Dioxygenases, methyltransferases, acetyltransferases, kinases, ubiquitinases. Cell-free and cellular biochemistry/enzymology assays from various companies. High-content image analyses in situ. In vitro reporter systems (locus repression/derepression and CRISPR-Cas9 guided).
**2. Absolute global genome changes:**
Robust measures of multiple epigenetic marks within the same sample by LC–MS/MS, CE–MS. Histone modifications. DNA covalent modifications.
**3. Index of global genome changes in DNAm:**
Proportion of cells differentially methylated by flow cytometry. Cytosine extension assays. DNA repeats. Activation of mutagenic retrotransposons (LINES, SINES). Structural DNA repeats and genetic stability (satellite-α, satellite-2).
**4. Markers of specific key events (tissue/species specific):**
Detoxification and DNA repair pathway: GSTP1, MGMT, and BRCA1. Cell cycle regulation: *INK4b/ARF/INK4a* locus (p15^Ink4b^, p16^Ink4a^, p19^Arf^). Inflammation/immune response: Major histocompatibility complex -I and –II (NLRC5 and HLA-A), T cell immune checkpoints, NK cell NKG2D receptor and their ligands (PD-L1, CTLA4, ULBP1/2/3, and MICA/B). Cancer-testis antigen gene families (Type-I MAGE and PRAME). Cell morphology and cytoskeleton: Links to global genome epigenetic marks, E-CADHERIN, and MYO10. Angiogenesis: Thrombospondin-1. Senescence bypass: Telomerase reverse transcriptase (TERT) regulation by TERT Hypermethylated Oncological Region (*Thor*). Other reprograming genes: Imprinted genes, oncogenes, homeobox (HOX) genes, Yamanaka reprogramming transcription factors OCT4 (also known as POU5F1), SOX2, cMYC, and KLF4.
**5. Next-generation sequencing epigenetic methodologies:**
Transcriptomic analyses of epigenetic driver genes. 25 epigenetic genes that were found to be drivers of carcinogenesis in human cells: SET1, MLL1, KDM5, G9A, SUV39H1, SETDB1, EZH2, JMJD3, CBX7, CBX8, BMI, SUZ12, HP1, MPP8, DNMT1, DNMT3A, DNMT3B, TET1, MeCP2, SETDB2, BAZ2A, UHRF1, CTCF, HOTAIR and ANRIL. DNAm signatures, multiplex assays. WGBS, RRBS, Dnase-Seq, ATAC-Seq, …

**Table 4:** Provisional selection of epigenetic assays for further validation in the context of chemical hazard assessment. Schematic mechanistic representation of assays for *Sat-α*, H3K9me2/3, and H4K20me2/3 requiring further validation/development are presented in Figure 4 and Figure 5.

	Screening	Potential to Predict Key Events and to Provide a Comprehensive Analysis
In vitro Short-term enzymology	Commercial enzyme biochemistry assay (cell free = metabolism free) High-content image analyses (effect of metabolism)	Limited under short-term experiment
In vitro Heritable epigenetic memory/reprograming (Long-term > 3 weeks)	*Sat-α* DNAm: a more targeted endpoint with a larger dynamic range than *L1* and *AluYb8* (genomic instability) H3K9me2/3 (heterochromatin to euchromatin) H4K20me3 (dysfunctional DNA repair)	NGS-based methodologies for DNA or HPTM followed by validation, pathway analyses, and demonstration of affected pathway
In vivo Heritable epigenetic memory/reprograming (Long-term > 3 weeks)	*Sat-α* DNAm: a more targeted endpoint with a larger dynamic range than *L1* and *AluYb8* (genomic instability) H3K9me2/3 (heterochromatin to euchromatin) H4K20me3 (dysfunctional DNA repair)	NGS-based methodologies for DNA or HPTM followed by validation, pathway analyses, and demonstration of affected pathway

## Supplementary Materials

The following are available online at https://www.mdpi.com/article/10.3390/ijms222010969/s1.

## Abbreviations

5aCdR	5-aza-2′-deoxycytidine.
5hmC	5-hydroxymethylcytosine.
5hmC	hydroxymethyl cytosine.
5mC	5-methylcytosine.
AE	adverse event.
ALT	alternative lengthening of telomeres pathway.
Alu	arthrobacter luteus DNA repeats.
AML	acute myeloid leukemia.
AOP	adverse outcome pathway.
APC	adenomatous polyposis coli protein.
B[a]P	benzo[a]pyrene.
BCAT1	branched-chain-amino-acid aminotransferase, cytosolic.
BEAS-2B	human bronchial epithelial SV-40 immortalised cell line.
BMD	benchmark-doses.
BMP3	bone morphogenic protein 3.
BPA	bisphenol-A.
CAN	copy number alterations.
CAR	constitutive androstane receptor.
cGAS	cyclic GMP-AMP synthase.
CTA	cell transformation assay.
CTAG	cancer-testis antigens.
CTCF	CCCTC-binding factor.
DNAm	DNA methylation.
DNMT1, 2, 3a, 3b, 3L	DNA methyltransferases.
Ecad	E-cadherin.
EMT	epithelial to mesenchymal transition.
GGD	global genome DNA.
GGDHo	global genome DNA hypomethylation.
GGDm	global genome DNA methylation.
GSH	glutathione.
GSTP1	glutathione S-transferase Pi1.
GTxC	genotoxic carcinogens.
HA	hazard assessment.
HaCaT	human keratinocyte immortalized cell line.
HBE	human bronchial epithelial cell line.
HCA	high-content analysis.
HDAC	histone deacetylase.
HDM	histone demethylase.
hESC	human embryonic stem cells.
HMEC	human mammary epithelial cells.
HMT	histone methyltransferase.
HPTM	histone post-translational modifications.
HTS	high-throughput screening.
HUC1	human urothelial immortalised cell line.
IARC	International Agency for the Research on Cancer.
IATA	integrated approach to the testing and assessment.
IDH	isocitrate dehydrogenase.
IKZF1	DNA-binding protein Ikaros.
KDM	lysine demethylase.
KE	key event.
KER	key event relationship.
KMT	lysine methyltransferase.
L-02	human hepatic cell line.
Line	long interspersed nuclear element.
LncRNA	long non-coding RNA.
LOAELs	lowest-observable-adverse-effect levels.
LTR	long terminal repeat.
MCF7	breast cancer cell line #7 from the Michigan Cancer Foundation.
mESC	mouse embryonic stem cells.
MGMT	methylated DNA-protein-cysteine methyltransferase.
MHC	major histocompatibility complex.
MIE	molecular initiating event.
MS	mass spectrometry.
NAM	novel approach methodologies.
ncRNA	non-coding RNA.
NDRG4	N-myc downregulated gene 4.
NGS	next-generation sequencing.
NGTxC	non-genotoxic carcinogens.
NOAELs	no-observable-adverse- effect levels.
OECD	Organization for Economic Cooperation and Development,
OGG1	8-oxoguanine glycosylase.
ONECUT2	one cut domain family member 2.
OTX1	homeobox protein OTX1.
PBMC	peripheral blood mononuclear cells.
PCB	polychlorinated biphenyls.
PCNA	proliferative cell nuclear antigen.
PHD	prolyl hydroxylase dioxygenase.
PND	postnatal day.
PoD	point of departure.
RA	risk assessment.
RASSF1	ras association domain-containing protein 1.
RCB	two-year rodent cancer bioassay.
RRBS	reduced-representation bisulphite sequencing.
SAHA	suberoylanilide hydroxamic acid.
SAM	s-adenosylmethionine.
09-Sep	septin-9.
SHOX2	short stature homeobox protein 2.
Sine	short interspersed nuclear element.
SUV39H1	suppressor of variegation 3–9 homolog 1; catalyzes H3K9me3.
TCA	tricarboxylic acid cycle.
TCDD	2,3,7,8-tetrachlorodibenzo-p-dioxin.
TDG	thymine-DNA glycosylase.
TET-1, -2, -3	ten eleven translocation enzymes.
TSG	tumour suppressor gene.
TSS	transcription start sites.
TWIST1	twist-related protein 1.
UHRF1	ubiquitin-like PHD and RING finger domain 1.
WGBS	whole-genome bisulphite sequencing.
WoE	weight of evidence.
