# Supplementary material for: Integration of Epigenetic Mechanisms into Non-Genotoxic Carcinogenicity Hazard Assessment: Focus on DNA Methylation and Histone Modifications
Source: Int J Mol Sci. 2021 Oct 11;22(20):10969. doi: 10.3390/ijms222010969 (PMC8535778; doi:10.3390/ijms222010969)
Supplement: Supplementary file 1 [file ijms-22-10969-s001.zip › ijms-1373717-supplementary.pdf]

International Journal of *Molecular Sciences*

MDPI

Supplementary Tables S1, S2, and S3

Table S1. Examples of studies demonstrating progressive changes in DNAm leading to cancers, or field effects.

**Progressive Changes**		
**Cancer Type**	**Progression**	Authors
Lung cancer	Global genome DNA hypomethylation in tumor-associated fibroblast and non-small cell lung cancer.	[1]
Melanoma	From benign nevi to malignant primary lesions to metastasis (also describe inactivation of cell-adhesion, then activation of inflammatory and immune system impairments).	[2]
Gastric cancer	From gastritis (*H. pylori*) to metaplasia and gastric cancer.	[3;4]
Oral squamous cell carcinoma (OSCC)	Within patient's progression from normal biopsies adjacent to dysplasia, to carcinoma *in situ* (CIS) and OSCC.	[5]
Oral squamous cell carcinoma (OSCC)	From chronic inflammatory disease (chronic periodontitis) to pre-neoplasia.	[6]
Field effects*		
Bladder cancer	L1 hypomethylation in normal urothelium from bladder containing cancers and in bladder cancers.	[7]
Pancreatic cancer	Sat-II RNA expression in normal tissue, in non-cancerous tissues adjacent to pancreatic ductal adenocarcinoma.	[8]
Prostate cancer		[9]
Colon cancer	Normal tissue surrounding colon cancer.	[10]
Ovarian cancer	L1 hypomethylation and expression in normal-appearing fallopian tube epithelium, in serous tubal intraepithelial carcinomas, and in ovarian carcinomas.	[11]
Breast cancer	Gene specific methylation in tissues surrounding cancer.	[12] [13]

*Field effects support epigenetic disruption as an early event in carcinogenesis, or as marker of the presence of a cancer in the surrounding tissues.

Table S2. Histone post translational modifications (HPTM) induced by arsenic in short- and long-term experiments.

**Model**	**Type**	**Treatment**		**Effects**	**Outcome**	**Authors**
		**Dose**	**Length**			
**CD-1 mice lungs and liver**	**In vivo**	**Dimethylarsinic acid (DMA), 200 ppm**	**Trans-placental gestation day 8-18**	84 weeks of age. In the lung: ↗H3K9me2, but no effect on H3K27me3	Increase incidences of total tumours, of lung adenocarcinomas, and of hepatocarcinomas in males.	[14]
Human blood lymphocytes	In vivo	Ranked in four groups from no signs to severe cases of arsenicosis	Chronic	↗H3K36me3 from mild to severe cases. ↘H3K9me2 from mild to severe cases. ↘H4K20me2 in severe cases	Arsenicosis	[15]
	In vivo	Low vs high inorganic arsenic (monomethylarsonous acid) exposure (10–30 µg/l vs > 150 µg/l in drinking water)	Chronic	↗H3K18ac ↘H4K8ac No significant changes for H3K23ac and H4K12ac.		[16]

HaCaT Human keratinocytes	Cell line	Sodium arsenite (NaAsO2) 0, 1.25, 2.5, 5, or 10 μM	24h	↘ in global genome H3K9me2, but ↗ H3K9me2 in promoter of MPG, XRCC1, and PARP1, reducing their expression.	Arsenic reduce expression of base excision repair genes (MPG, XRCC1, and PARP1) aggravating DNA damage.	[17]
	Cell line	Sodium arsenite (NaAsO2) 0.0, 2.5, 5, 10, and 20 μM	24h	↘ H3K18ac ↘ nucleotide excision repair related genes (XPA, XPD, and XPF).	Increase in DNA damage.	[18]
L-02 Normal human liver cells	Cell line	Sodium arsenite (NaAsO2) 0, 5, 10, 20 μM	24h	↗ KDM3A/JHDM2A ↘ global H3K9me2 ↗ H3K9me2 in promoters of base excision repair genes	Increase in DNA damage.	[19]
HepaRG human non-tumorigenic liver cells	Cell line	Sodium arsenite (NaAsO2) 1 μM	14 days	Global genome DNA hypomethylation. Site specific DNA hypermethylation (e.g. *CLDN14*). ↘ H3K36me3. ↗ H4K20me3. ↗ DNA damage.	Induction of carcinogenesis-related events, epithelial-to-mesenchymal transition based on gene expression, damage to DNA, inhibition of DNA repair genes. No effect on H3K4me3, H3K9me3, H3K27me3, H3K9ac, H3K27ac, H4K16ac	[20]
A/J mice lungs	In vivo	Sodium arsenite NaAsO2, 100 μl behind the tongue every other day for one week from 100 and 200 μg/L solutions		↗ H3.1 mRNA. ↘ SLBP mRNA and proteins. In nude mice, FH3.1poly(A)-expressing BEAS-2B cells were tumorigenic.	Confirmed *in vivo* effects of arsenic observed *in vitro*.	[21]
BEAS-2B human broncheal epithelial cell line	Cell line	Sodium arsenite NaAsO2, 0.5 and 1 μM	96h	↗ polyadenylation of H3.1 mRNA. ↗ H3.1 protein. ↘ H3.3 at promoters, enhancers, insulators. Effects of arsenic on polyadenylation counteracted by SLBP expression.	Transcriptional deregulation, cell cycle arrest, aneuploidy, chromosomal aberrations, colony formation in soft agar transformation.	[21]
	Cell line	20 μM As^{3}	2-24h	No effect on H3K27me3. As^{3+} induces EZH2* S21 phosphorylation in BEAS-2B through JNK and STAT3-dependent Akt activation. As^{3+} increase expression of miR21 which	This work reveal a signaling cascade induced by As^{3+} within less than 24h that may prevent normal methylation of H3K27. In response to As3+ treatment, JNK	[22]

				downregulate Spry2 (a negative regulator of Akt). As^{3+} induce cytosolic location of phosphorylated EZH2-S21.	phosphorylates STAT3 leading to increased expression of miR-21, which, in turn, downregulates the expression of Spry2 (a negative regulator of Akt signaling). The activated Akt phosphorylates EZH2 at S21 that mostly remain cytosolic. A reduction in EZH2 in the nuclei may reduce abundance of H3K27me3 and activate oncogenes.	
A549 Human lung carcinoma cells	Cell line	1-5 µM As^3	24h	↘H3K4me1. ↗H3K4me2, me3.		[23]
		0.1, 0.5, 1 µM	7d	↗H3K4me3 abundance.		
UROtsa Human urothelial cells (SV40 transformed, non tumorigenic)	Cell line	Sodium arsenite (NaAsO2) at 1,3,10 µM (As^3). Monomethylarsine oxide at 0.3,1 ,3 µM (MMA^3)	24h	↘H4K16ac at 3 µM MMA^3 and 10 µM As^3.	↘KAT8 (MYST1/hMOF) can sensitize cells to arsenic toxcicity	[24]
			7d	↘H4K16ac at 0.3 µM MMA^3 and 1 µM As^3		
	Cell line	50 nM MMA^{3+}	16 weeks	Specific study of H3K18ac over the genome.	Alteration of histone acetylation patterns in a time- and malignant stage-dependent aberrant gene-expression pattern.	[25]
	Cell line	50 nM MMA^{3+}	Time-series 4, 8, 10, 12, and 14weeks	↗ H3 ac and ↘H4 acetylation at the time of malignant transformation. Investigated series of of H3 and H4 lysine acetylation and H3 methylation.	Malignant cell transformation; colony formation in soft agar and nude mice xenograft assay	[16]
HEK293T human embryo kidney or HELAl	Cell line	0.2-0.8 µM arsenic trioxide As_2O_3	24h, 48h, 72h	↘H4K16ac. ↘ HAT act MYST1 via As-Zn finger domain direct interaction		[26]
NHEK cells, primary culture of normal human epithelial. Keratinocytes.	Primary	0.5 µM As^3	24h up to 10 weeks	↗H4K16ac from Day 1 to 48. DNA hyper and hypomethylation of SIRT1** and pri-miR-34a, from week 5 to 10.	Cumulative disruptions to epigenetic regulation of miR-34a expression, SIRT1, polycomb repressive group complex, and	[27]

			Downregulation of SIRT1 and miR-34a over the first 3 weeks.	p53 functional activities.

*EZH2 is the enzymatic subunit of the PRC2 complex responsible for the trimethylation H3K27me3. EZH2 S21 phosphorylation facilitates the dissociation of the PRC2 complex which may reduce the methyltransferase activity of EZH2 toward H3K27. **SIRT1 is recruited to methylation sites as part of polycomb repressive (PRG) complexes 2 and 4, where it associates with and regulates EZH2, EED, SUZ12 and DNMT1. SIRT1 coordinates chromatin remodeling during oxidative DNA damage by deacetylating H4 at Lys 16 (H4K16) and maintains cytosine methylation by recruiting and activating DNMT1. miR-34a is a regulatory component of p53 mediated signaling. mIr-34a whose expression is induced by p53-mediated transcription activation) regulates expression of SIRT1 by base pairing with its 3'UTR. p53/SIRT1/miR-34a form a coherent feed-forward loop (the p53/SIRT1/miR-34a axis).

Table S3. Arsenical exposures revealing altered DNA methylation mechanisms.

Substance	Treatment duration	Cell/tissue type	Endpoint	Authors
Arsenic trioxide (As_2O_3)	48 h 2 to 10 μM	HepG2	Decrease radiometric DNMT activity on artificial substrate & DNMT mRNA	[28]
Sodium arsenite ($NaAsO_2$)	24 h 25 μM	HaCaT	Decrease SAM	[29]
Sodium arsenite ($NaAsO_2$)	72 h 0.5, 1.5, and 5 μM	HaCaT	Decrease in DNMT1 and 3a mRNA	[29]
Sodium arsenite ($NaAsO_2$)	8 weeks	BEAS-2B	DNMT1, 3a, 3b, mRNA & protein	[30]
Sodium arsenite ($NaAsO_2$)	8 weeks 0.5 μM	BEAS-2B	Increases in TET enzyme expression. Redistribution and increase in global 5hmC. EMT based on mRNA.	[31]
Sodium arsenite ($NaAsO_2$)	24 h 1.0 μM	HaCaT	DNA methylation dependent expression of let-7 miR family member	[32]
Sodium arsenite ($NaAsO_2$)	16 weeks 2.5 μM	$p53^{low}$ HBEC	EMT, DNA methylation suppression of miR200	[33] [34]
As human blood contaminant	Chronic	Blood samples	Review of LINE-1, Alu, global genome demethylation	[35]

References

1. Vizoso, M.; Puig, M.; Carmona, F.J.; Maqueda, M.; Velasquez, A.; Gomez, A.; Labernadie, A.; Lugo, R.; Gabasa, M.; Rigat-Brugarolas, L.G.; Trepat, X.; Ramirez, J.; Moran, S.; Vidal, E.; Reguart, N.; Perera, A.; Esteller, M.; Alcaraz, J. Aberrant DNA methylation in non-small cell lung cancer-associated fibroblasts. *Carcinogenesis* **2015**,*36*:1453-1463.
2. Wouters, J.; Vizoso, M.; Martinez-Cardus, A.; Carmona, F.J.; Govaere, O.; Laguna, T.; Joseph, J.; Dynoodt, P.; Aura, C.; Foth, M.; Cloots, R.; van den Hurk, K.; Balint, B.; Murphy, I.G.; McDermott, E.W.; Sheahan, K.; Jirstrom, K.; Nodin, B.; Mallya-Udupi, G.; van den Oord, J.J.; Gallagher, W.M.; Esteller, M. Comprehensive DNA methylation study identifies novel progression-related and prognostic markers for cutaneous melanoma. *BMC Med.* **2017**,*15*:101.
3. Kurklu, B.; Whitehead, R.H.; Ong, E.K.; Minamoto, T.; Fox, J.G.; Mann, J.R.; Judd, L.M.; Giraud, A.S.; Menheniott, T.R. Lineage-specific RUNX3 hypomethylation marks the preneoplastic immune component of gastric cancer. *Oncogene* **2015**,*34*:2856-2866.
4. Pirini, F.; Noazin, S.; Jahuira-Arias, M.H.; Rodriguez-Torres, S.; Friess, L.; Michailidi, C.; Cok, J.; Combe, J.; Vargas, G.; Prado, W.; Soudry, E.; Perez, J.; Yudin, T.; Mancinelli, A.; Unger, H.; Ili-Gangas, C.; Brebi-Mieville, P.; Berg, D.E.; Hayashi, M.;

Sidransky, D.; Gilman, R.H.; Guerrero-Preston, R. Early detection of gastric cancer using global, genome-wide and IRF4, ELMO1, CLIP4 and MSC DNA methylation in endoscopic biopsies. *Oncotarget* **2017**,*8*:38501-38516.
5. Towle, R.; Truong, D.; Hogg, K.; Robinson, W.P.; Poh, C.F.; Garnis, C. Global analysis of DNA methylation changes during progression of oral cancer. *Oral Oncol* **2013**,*49*:1033-1042.
6. Planello, A.C.; Singhania, R.; Kron, K.J.; Bailey, S.D.; Roulois, D.; Lupien, M.; Line, S.R.; de Souza, A.P.; De Carvalho, D.D. Preneoplastic epigenetic disruption of transcriptional enhancers in chronic inflammation. *Oncotarget* **2016**,*7*:15772-15786.
7. Wolff, E.M.; Byun, H.M.; Han, H.F.; Sharma, S.; Nichols, P.W.; Siegmund, K.D.; Yang, A.S.; Jones, P.A.; Liang, G. Hypomethylation of a LINE-1 promoter activates an alternate transcript of the MET oncogene in bladders with cancer. *PLoS Genet.* **2010**,*6*:e1000917.
8. Kishikawa, T.; Otsuka, M.; Yoshikawa, T.; Ohno, M.; Yamamoto, K.; Yamamoto, N.; Kotani, A.; Koike, K. Quantitation of circulating satellite RNAs in pancreatic cancer patients. *JCI Insight* **2016**,*1*:e86646.
9. Van, N.L.; Groskopf, J.; Grizzle, W.E.; Adams, G.W.; DeGuenther, M.S.; Kolettis, P.N.; Bryant, J.E.; Kearney, G.P.; Kearney, M.C.; Van, C.W.; Gaston, S.M. Epigenetic risk score improves prostate cancer risk assessment. *Prostate* **2017**,*77*:1259-1264.
10. Sugai, T.; Yoshida, M.; Eizuka, M.; Uesugii, N.; Habano, W.; Otsuka, K.; Sasaki, A.; Yamamoto, E.; Matsumoto, T.; Suzuki, H. Analysis of the DNA methylation level of cancer-related genes in colorectal cancer and the surrounding normal mucosa. *Clin. Epigenetics* **2017**,*9*:55.
11. Pisanic, T.R.; Asaka, S.; Lin, S.F.; Yen, T.T.; Sun, H.; Bahadirli-Talbott, A.; Wang, T.H.; Burns, K.H.; Wang, T.L.; Shih, I.M. Long Interspersed Element 1 Retrotransposons Become Deregulated during the Development of Ovarian Cancer Precursor Lesions. *Am. J. Pathol* **2018**.
12. Fernandez, S.V.; Snider, K.E.; Wu, Y.Z.; Russo, I.H.; Plass, C.; Russo, J. DNA methylation changes in a human cell model of breast cancer progression. *Mutat Res.* **2010**,*688*:28-35.
13. Rauscher, G.H.; Kresovich, J.K.; Poulin, M.; Yan, L.; Macias, V.; Mahmoud, A.M.; Al-Alem, U.; Kajdacsy-Balla, A.; Wiley, E.L.; Tonetti, D.; Ehrlich, M. Exploring DNA methylation changes in promoter, intragenic, and intergenic regions as early and late events in breast cancer formation. *BMC Cancer* **2015**,*15*:816.
14. Fujioka, M.; Suzuki, S.; Gi, M.; Kakehashi, A.; Oishi, Y.; Okuno, T.; Yukimatsu, N.; Wanibuchi, H. Dimethylarsinic acid (DMA) enhanced lung carcinogenesis via histone H3K9 modification in a transplacental mouse model. *Arch. Toxicol* **2020**,*94*:927-937.
15. Li, J.; Ma, L.; Wang, X.; Li, D.; Zeng, Q.; Xing, X.; Li, C.; Xie, L.; Chen, L.; Chen, W.; Zhang, A. Modifications of H3K9me2, H3K36me3 and H4K20me2 may be involved in arsenic-induced genetic damage. *Toxicol Res. (Camb)* **2016**,*5*:1380-1387.
16. Ge, Y.; Zhu, J.; Wang, X.; Zheng, N.; Tu, C.; Qu, J.; Ren, X. Mapping dynamic histone modification patterns during arsenic-induced malignant transformation of human bladder cells. *Toxicol Appl Pharmacol* **2018**,*355*:164-173.
17. Ding, X.; Zhang, A.; Li, C.; Ma, L.; Tang, S.; Wang, Q.; Yang, G.; Li, J. The role of H3K9me2-regulated base excision repair genes in the repair of DNA damage induced by arsenic in HaCaT cells and the effects of Ginkgo biloba extract intervention. *Environ. Toxicol* **2021**,*36*:850-860.
18. Zhang, A.L.; Chen, L.; Ma, L.; Ding, X.J.; Tang, S.F.; Zhang, A.H.; Li, J. Role of H3K18ac-regulated nucleotide excision repair-related genes in arsenic-induced DNA damage and repair of HaCaT cells. *Hum. Exp. Toxicol* **2020**,*39*:1168-1177.
19. Zhang, A.L.; Tang, S.F.; Yang, Y.; Li, C.Z.; Ding, X.J.; Zhao, H.; Wang, J.H.; Yang, G.H.; Li, J. Histone demethylase JHDM2A regulates H3K9 dimethylation in response to arsenic-induced DNA damage and repair in normal human liver cells. *J. Appl Toxicol* **2020**,*40*:1661-1672.
20. Tryndyak, V.P.; Borowa-Mazgaj, B.; Steward, C.R.; Beland, F.A.; Pogribny, I.P. Epigenetic effects of low-level sodium arsenite exposure on human liver HepaRG cells. *Arch. Toxicol* **2020**.
21. Chen, D.; Chen, Q.Y.; Wang, Z.; Zhu, Y.; Kluz, T.; Tan, W.; Li, J.; Wu, F.; Fang, L.; Zhang, X.; He, R.; Shen, S.; Sun, H.; Zang, C.; Jin, C.; Costa, M. Polyadenylation of Histone H3.1 mRNA Promotes Cell Transformation by Displacing H3.3 from Gene Regulatory Elements. *iScience* **2020**,*23*:101518.
22. Chen, B.; Liu, J.; Chang, Q.; Beezhold, K.; Lu, Y.; Chen, F. JNK and STAT3 signaling pathways converge on Akt-mediated phosphorylation of EZH2 in bronchial epithelial cells induced by arsenic. *Cell Cycle* **2013**,*12*:112-121.
23. Zhou, X.; Li, Q.; Arita, A.; Sun, H.; Costa, M. Effects of nickel, chromate, and arsenite on histone 3 lysine methylation. *Toxicol Appl Pharmacol* **2009**,*236*:78-84.
24. Jo, W.J.; Ren, X.; Chu, F.; Aleshin, M.; Wintz, H.; Burlingame, A.; Smith, M.T.; Vulpe, C.D.; Zhang, L. Acetylated H4K16 by MYST1 protects UROtsa cells from arsenic toxicity and is decreased following chronic arsenic exposure. *Toxicol Appl Pharmacol* **2009**,*241*:294-302.
25. Zhu, J.; Wang, J.; Chen, X.; Tsompana, M.; Gaile, D.; Buck, M.; Ren, X. A time-series analysis of altered histone H3 acetylation and gene expression during the course of MMAIII-induced malignant transformation of urinary bladder cells. *Carcinogenesis* **2017**,*38*:378-390.
26. Liu, D.; Wu, D.; Zhao, L.; Yang, Y.; Ding, J.; Dong, L.; Hu, L.; Wang, F.; Zhao, X.; Cai, Y.; Jin, J. Arsenic Trioxide Reduces Global Histone H4 Acetylation at Lysine 16 through Direct Binding to Histone Acetyltransferase hMOF in Human Cells. *PLoS ONE* **2015**,*10*:e0141014.
27. Herbert, K.J.; Holloway, A.; Cook, A.L.; Chin, S.P.; Snow, E.T. Arsenic exposure disrupts epigenetic regulation of SIRT1 in human keratinocytes. *Toxicol Appl Pharmacol* **2014**,*281*:136-145.
28. Cui, X.; Wakai, T.; Shirai, Y.; Yokoyama, N.; Hatakeyama, K.; Hirano, S. Arsenic trioxide inhibits DNA methyltransferase and restores methylation-silenced genes in human liver cancer cells. *Hum. Pathol* **2006**,*37*:298-311.

29. Reichard, J.F.; Schnekenburger, M.; Puga, A. Long term low-dose arsenic exposure induces loss of DNA methylation. *Biochem Biophys Res. Commun* **2007**,*352*:188-192.
30. Rea, M.; Eckstein, M.; Eleazer, R.; Smith, C.; Fondufe-Mittendorf, Y.N. Genome-wide DNA methylation reprogramming in response to inorganic arsenic links inhibition of CTCF binding, DNMT expression and cellular transformation. *Sci Rep.* **2017**,*7*:41474.
31. Rea, M.; Gripshover, T.; Fondufe-Mittendorf, Y. Selective inhibition of CTCF binding by iAs directs TET-mediated reprogramming of 5-hydroxymethylation patterns in iAs-transformed cells. *Toxicol Appl Pharmacol* **2018**,*338*:124-133.
32. Jiang, R.; Li, Y.; Zhang, A.; Wang, B.; Xu, Y.; Xu, W.; Zhao, Y.; Luo, F.; Liu, Q. The acquisition of cancer stem cell-like properties and neoplastic transformation of human keratinocytes induced by arsenite involves epigenetic silencing of let-7c via Ras/NF-kappaB. *Toxicol Lett* **2014**,*227*:91-98.
33. Wang, Z.; Yang, C. Metal carcinogen exposure induces cancer stem cell-like property through epigenetic reprograming: A novel mechanism of metal carcinogenesis. *Semin Cancer Biol* **2019**.
34. Wang, Z.; Zhao, Y.; Smith, E.; Goodall, G.J.; Drew, P.A.; Brabletz, T.; Yang, C. Reversal and prevention of arsenic-induced human bronchial epithelial cell malignant transformation by microRNA-200b. *Toxicol Sci* **2011**,*121*:110-122.
35. Paul, S.; Bhattacharjee, P.; Giri, A.K.; Bhattacharjee, P. Arsenic toxicity and epimutagenecity: The new LINEage. *Biometals* **2017**,*30*:505-515.
